# Pyntacle: a parallel computing-enabled framework for large-scale network biology analysis

**DOI:** 10.1093/gigascience/giaa115

**Published:** 2020-10-21

**Authors:** Luca Parca, Mauro Truglio, Tommaso Biagini, Stefano Castellana, Francesco Petrizzelli, Daniele Capocefalo, Ferenc Jordán, Massimo Carella, Tommaso Mazza

**Affiliations:** IRCCS Casa Sollievo della Sofferenza, Laboratory of Bioinformatics, Viale Cappuccini 1, 71013, San Giovanni Rotondo (FG), Italy; IRCCS Casa Sollievo della Sofferenza, Laboratory of Bioinformatics, Viale Cappuccini 1, 71013, San Giovanni Rotondo (FG), Italy; IRCCS Casa Sollievo della Sofferenza, Laboratory of Bioinformatics, Viale Cappuccini 1, 71013, San Giovanni Rotondo (FG), Italy; IRCCS Casa Sollievo della Sofferenza, Laboratory of Bioinformatics, Viale Cappuccini 1, 71013, San Giovanni Rotondo (FG), Italy; IRCCS Casa Sollievo della Sofferenza, Laboratory of Bioinformatics, Viale Cappuccini 1, 71013, San Giovanni Rotondo (FG), Italy; Department of Experimental Medicine, Sapienza University of Rome, Piazzale Aldo Moro 5, 00185, Rome, Italy; IRCCS Casa Sollievo della Sofferenza, Laboratory of Bioinformatics, Viale Cappuccini 1, 71013, San Giovanni Rotondo (FG), Italy; Balaton Limnological Institute, Centre for Ecological Research Klebelsberg Kuno 3, 8237 Tihany, Hungary; IRCCS Casa Sollievo della Sofferenza, Laboratory of Medical Genetics, Viale Padre Pio 7d, 71013, San Giovanni Rotondo (FG), Italy; IRCCS Casa Sollievo della Sofferenza, Laboratory of Bioinformatics, Viale Cappuccini 1, 71013, San Giovanni Rotondo (FG), Italy

## Abstract

**Background:**

Some natural systems are big in size, complex, and often characterized by convoluted mechanisms of interaction, such as epistasis, pleiotropy, and trophism, which cannot be immediately ascribed to individual natural events or biological entities but that are often derived from group effects. However, the determination of important groups of entities, such as genes or proteins, in complex systems is considered a computationally hard task.

**Results:**

We present Pyntacle, a high-performance framework designed to exploit parallel computing and graph theory to efficiently identify critical groups in big networks and in scenarios that cannot be tackled with traditional network analysis approaches.

**Conclusions:**

We showcase potential applications of Pyntacle with transcriptomics and structural biology data, thereby highlighting the outstanding improvement in terms of computational resources over existing tools.

## Background

Interactive systems are commonly represented as graphs (or networks), which are mathematical representations of “elements" (nodes) and their relationships (edges). The semantics of relationships is specific for each graph and completely defines its expressiveness. Protein interaction networks, for example, represent physical interactions as edges and proteins as nodes; metabolic networks wire metabolites whenever these participate in the same biochemical reactions; regulatory networks are directed graphs, where the directionality of relationships matters. Thus, a link exists between 2 molecules if there is evidence either of regulatory activity by a transcription factor onto a gene or of post-translational modifications. These, together with several other kinds of networks, such as RNA, signaling, neuronal, trophic, and co-expression networks, are the concrete signs of an exceptional growth of molecular interaction data and, hence, of an intense research activity in the field of network medicine [1].

Network medicine is a relatively new discipline that exploits graph theory to identify key molecules in the human diseasome [2], together with their hidden molecular relationships. The general aim is that of reverse-engineering the mechanisms of pathogenesis of complex disorders and traits, whereby the etiology is notoriously convoluted. The diseasome is, in fact, a network where diseases are nodes and links represent relationships between the disease-associated cellular components. Determining such links would help identify the molecular relationships between phenotypes and the reasons for certain comorbidities and would positively affect diagnosis, treatment, and drug multi-purposing.

Certain kinds of biological networks share the feature of having a few relatively highly connected nodes, often called “hubs," suggesting that the molecules represented by hubs should play special biological roles. The first hypothesis of network medicine is that most known disease genes, which are non-essential, lie in the periphery of these networks and are far from hubs. On the contrary, at least in human cells, hub molecules are encoded by essential genes [3]. A database, Database of Essential Genes (DEG), exists that reports essential genes for some bacteria, archaea, and eukaryotes [4]. An interesting speculation is that, because of their many links, hubs are reasonably associated with disease genes [5–7], which in turn, by virtue of the local hypothesis of network medicine, exhibit increased tendency to interact with each other, all being involved in the same disease. Thus, molecular networks are not random but tightly organized on the basis of specific principles, according to which the effect of a central gene, which is eventually aberrant, reverberates on the gene products of neighboring genes in its network. Hence, the expression of a disease phenotype rarely results from an individual aberrant gene, rather from the harmonized effects of groups of related genes. This holds true also for other types of networks, ranging from ecological to evolutionary and chemical networks.

**Figure 1:**
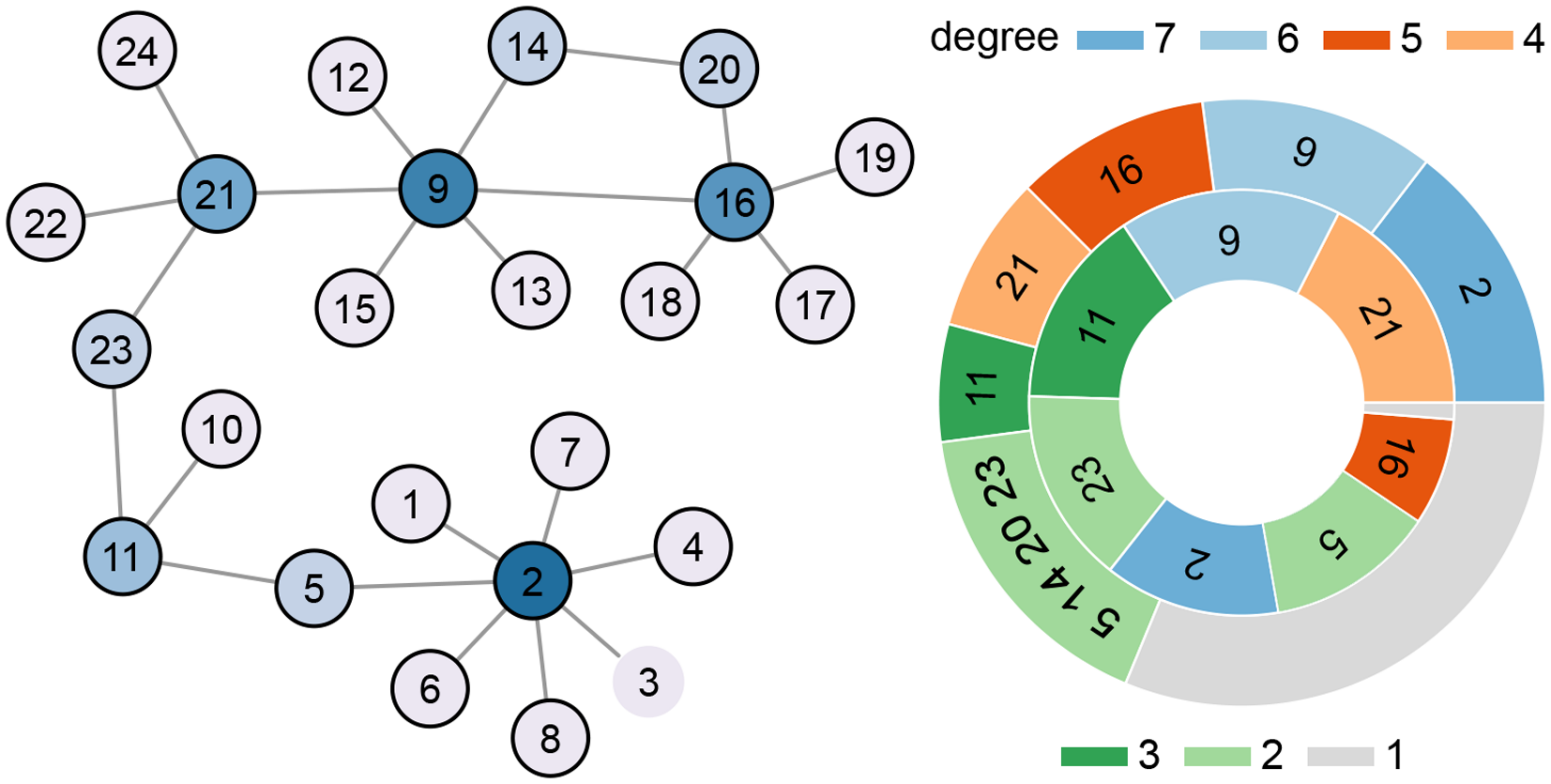
*Left:* Example network. The darker the blue color, the higher the degree of nodes. *Right:*Pie chart of the most central nodes. The outer circle reports the highest-degree nodes (counterclockwise, blue through gray). The inner circle represents the highest betweenness nodes, from light orange to gray, counterclockwise. Node names are reported within circle sectors. Sector width is proportional to the degree (outer) and betweenness (inner) values of nodes. Gray sectors contain unimportant nodes, i.e., nodes with unitary degree and negligible betweenness values.

Graph theory draws upon various tools to identify the most central elements, i.e., the key molecules, in a network. Here, the concept of centrality is synonymous with importance, even if the term has been declined differently in the literature. A topologically important node may be a hub, a “bottleneck," namely, a node that lies in many pathways, or one that is “close” to most other nodes. Despite the above description, local (i.e., regarding nodes or edges) and global (i.e., regarding the entire network) properties of networks are unlikely to completely explain the functioning of complex systems because they either fail to take into account or underestimate the effects that groups of important nodes may jointly exert on these systems. Node 2 in Fig. 1 has 7 ties, and it is the highest-degree node in this example network. It is connected with 7 unimportant nodes because these exhibit low degree values. Node 9 is the second-most connected node with only 1 fewer edges than Node 2, but 2 of its neighbors, i.e., Nodes 16 and 21, are the third- and fourth-ranked nodes by degree, with 5 and 4 ties, respectively. Thus, although Node 2 is top ranked by degree, it may not be the most functionally central node. Whether this assertion is true strictly depends on the purposes for which a network is being studied.

More interestingly, the “network parsimony" principle of network medicine, according to which causal molecular pathways often coincide with the shortest molecular paths between known disease-associated components, implies that it is fundamental to find the nodes that lie within the highest number of pathways in networks because these are more likely to be functionally critical [1]. The betweenness centrality index [8] is the most suitable for this task. Node 21 in Fig. 1 is the top-ranked node by betweenness. This was expected because it lies in the exact middle of the network, which, in turn, exhibits a quasi-tree topological node organization. But even if Node 21 belongs to almost all shortest paths of the network, it ranks only fourth by degree because it is not individually much connected. Node 9 is the second node by betweenness, with a score very close to that of Node 21, but, on the contrary, it ranks second by degree (Supplementary Data S1). Whether the most important node is 2, 21, or 9 depends on the aims and context of the study.

Whenever >1 node exhibits similar topological scores, as in this case, or when a co-responsibility for a phenotype is suspected, studying groups and their centrality might be a reasonable option. In 1999, Everett and Borgatti expanded the definition of degree, betweenness, and closeness to groups of nodes [9]. Calculating these indices for the following groups: {2, 21}, {9, 21}, {2, 9}, the latter achieved the highest scores. Moreover, considering the group made by all 3 nodes, only degree and closeness increased significantly in respect to {2, 9} (cf. Table 1).

**Table 1:** Group centrality metrics calculated for the example network.

Group	Degree	Betweenness	Closeness*
{2, 21}	0.5	0.39	0.58
{2, 9}	0.59	0.43	0.69
{21, 9}	0.36	0.35	0.38
{2, 9, 21}	0.71	0.45	0.75

Higher scores indicate higher centrality. * The “minimum" method was used to measure the distance from the group to an outside node.

In 2006, Borgatti introduced 2 other classes of metrics for groups that were meant to assess the ability of groups either to disrupt a network, when removed, or to efficiently spread information through a network. These were defined as “Key-Player Problem/Negative” (KPP-Neg) and “Key-Player Problem/Positive” (KPP-Pos), respectively [10]. Note that similar concepts were also covered in other research fields and scientific contexts [11, 12], where specific search strategies [13, 14] were implemented. KPP-Neg and KPP-Pos were calculated for the same groups and reported in Table 2. It is interesting to notice that {2, 9} is still the most important group in terms of disruption potential and connectivity. Their scores were slightly lower than those of group {2, 9, 11}, meaning that even here Node 11 does not contribute significantly to the centrality of {2, 9}.

**Table 2:** KPP-Neg and KPP-Pos metrics calculated for the example network

Group	DF (0.66)	m-reach*	DR
{2, 21}	0.87	$79.2\%$	0.65
{2, 9}	0.91	$95.8\%$	0.72
{21, 9}	0.84	$62.5\%$	0.53
{2, 9, 21}	0.93	$95.8\%$	0.74

DF (Neg) achieves its maximum value of 1.0 when the graph consists entirely of isolated nodes. m-reach (Pos) is a count of the number of unique nodes reached by any member of the group in *m* links or fewer. DR (Pos) achieves a maximum value of 1 when every non-group node is adjacent to ≥1 member of the group. * The *m* parameter of the algorithm was set to 2. The percentage of nodes reached by the group, including the group nodes, is reported.

**Figure 2:**
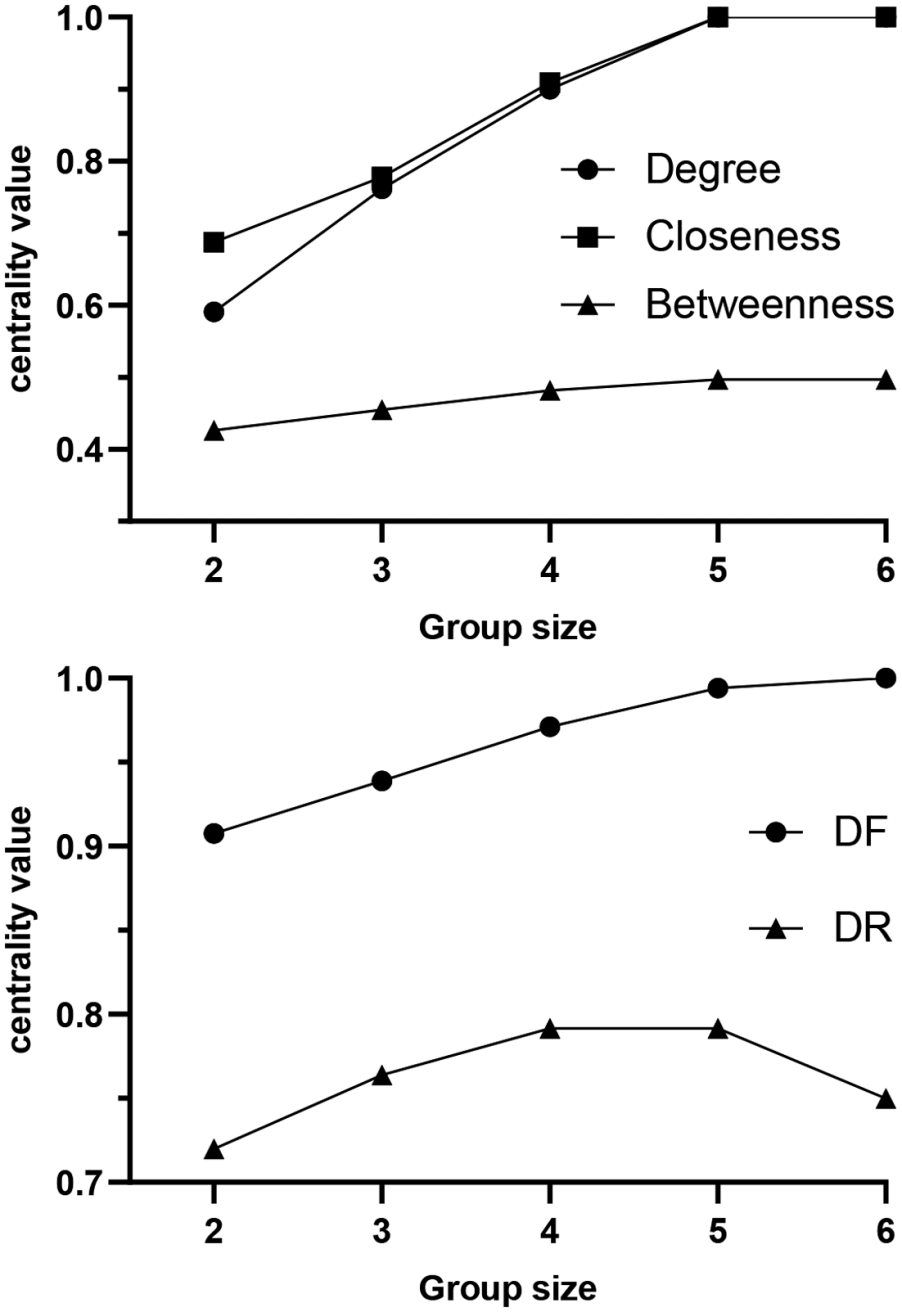
Brute-force search algorithm applied to all groups of sizes 2–6. For any size, the maximum scores obtained for (*top*) group degree, group closeness, and group betweenness and (*bottom*) DF and DR are plotted.

What remains to be verified is whether any other group exists that exhibits similar or higher centrality values. Considering the small network size, the option of running a “brute-force" algorithm to search the absolute best group(s) among all possible ones is computationally feasible, in place of a “greedy optimization" search, as suggested by Borgatti [10]. In this case, the best group of size 2 for all metrics is still {2, 9}, whereas {5, 9} reaches $100\%$ of non-group nodes and is ranked first by m-reach. However, because none of the centrality scores of {2, 9} equaled their maximum possible values, we again applied the brute-force search to groups of increasing sizes, 3–6. We thus found that degree and closeness reached their absolute maximum scores, i.e., 1, equally with 2 groups {2, 9, 11, 16, 21}, {2, 9, 10, 16, 21} of size 5, meaning that nodes 10 and 11 are interchangeable and equally important; betweenness obtained its maximum score (0.497) with the group {2, 9, 11, 16, 21} (Supplementary Data S2). The best group by DF is {2, 9, 11, 14, 16, 21}, which achieves the score of 1. The groups {2, 9, 10, 16, 21} and {2, 9, 11, 16, 21} equally obtained the best DR score (0.792). It is worth noticing that DR and betweenness do not reach their absolute maximum scores, which however are plausibly the highest possible for this network, because groups of bigger sizes exhibit lower scores (Fig. 2). It is also interesting to notice that Nodes 2, 9, and 21 are included in all groups described above, thereby highlighting their central roles in the network (Supplementary Data S3).

**Figure 3:**
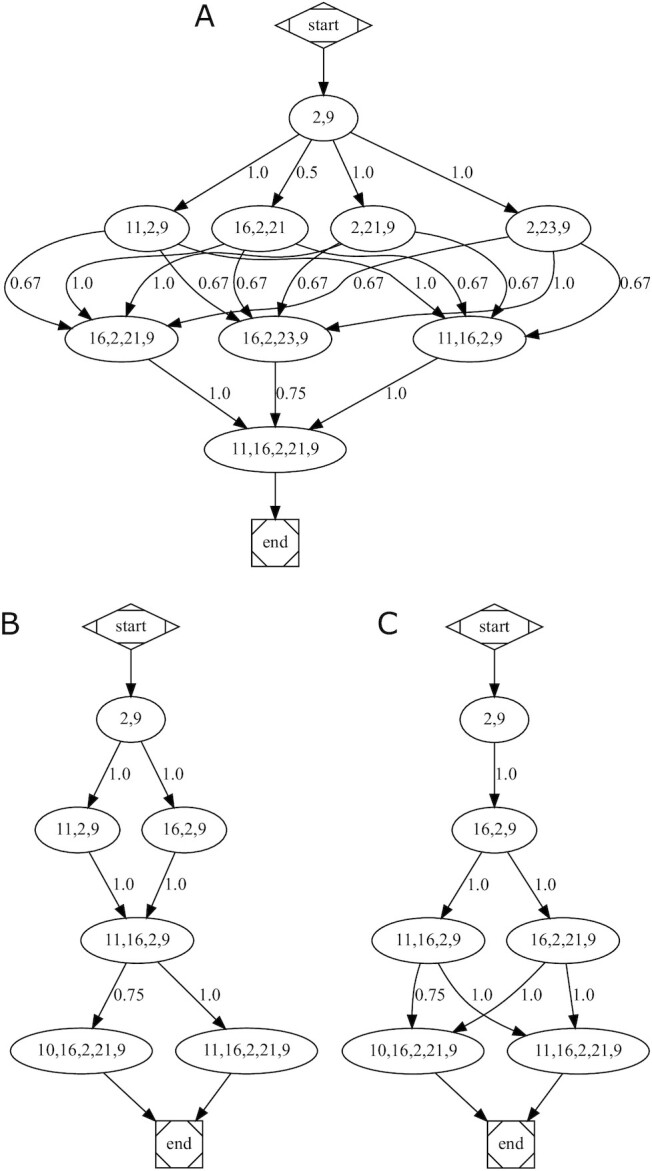
Nestedness graphs for group (A) betweenness, (B) closeness, and (C) degree centrality metrics. Nodes represent groups with top centrality values in respect to all other possible groups of nodes with same sizes. Edges connect groups when the bigger group contains ≥1 element of the smaller one. Edges are labeled with the overlap ratio between the elements of the connected groups.

Computing the nestedness, which consists in verifying whether sets of nodes recur in groups of increasing sizes, could confirm the importance of Nodes 2, 9, and 21. Hence, if larger sets contain smaller sets, higher values of nestedness may be a proxy for identifying upstream/master regulators through the key nodes of the smallest groups. One way to calculate the nestedness of the example network is by the Nrow metrics [15, 16]. Nrow is defined as the average percentage of nodes from smaller sets that are contained in larger sets, taking all possible pairs of sets. Thus, after computing all the best sets of increasing sizes, from 2 to 5, for each group centrality metric but m-reach, Nodes 2 and 9, and not 21, resulted in being nested in all sets, regardless of their size (Fig. 3 and Supplementary Data S2). The same evidence emerged with the key-player metrics (Supplementary Data S3). The nestedness scores were generally quite high, meaning that nodes are not interchangeable among groups, i.e., there are few equally important nodes. The group {2, 9} is definitely important from a topological point of view, and its discovery would not have been immediately hypothesized without this investigation because Nodes 2 and 9 are 5 links apart.

This “practical” introduction aims at introducing the theory underlying Pyntacle. A toy model was used to describe the main features, outline a possible analytical pathway, and highlight how Pyntacle may help extract valuable information from real-world networks. The rest of the article thus presents (i) the software and its main components; (ii) its design and implementation; (iii) details of how to use it; (iv) benchmarks, assessed on real and simulated networks of increasing sizes, in comparison with a similar software package; and (v) 2 real-world case studies.

## Pyntacle

Pyntacle is an open-source network analysis framework that was originally designed to tackle the Borgatti Key-Player Problem [10] efficiently through the identification of maximally reachable or disruptive groups of nodes. Contrary to similar software packages that either analyze networks with standard global and local topological metrics [17, 18] or provide the users with limited tools to detect important groups of nodes [19], Pyntacle adopts optimized heuristic algorithms and parallel computing strategies to make the task of identifying key-player nodes feasible. It has the following attributes: (i) is available for Windows, Mac, and Linux OS; (ii) is available as both command line tool and API, with an easy and user-friendly interface for both input commands and results visualization; and (iii) allows the management of real-world graphs in a computationally efficient way.

Pyntacle is implemented in modules, each designed to analyze a particular aspect of a network. These can calculate global and local topological metrics (metrics module), the importance of groups of nodes (groupcentrality and keyplayer modules), search and analyze clusters of nodes (communities module), perform set operations between networks (set module), generate networks with different topological organizations (e.g., random, scale-free, and small-world networks, generate module), and convert and load/save networks using different data formats (e.g., adjacency matrix, edge list, SIF and dot, convert module).

### Features

#### Centrality measures for groups

Pyntacle tackles the problem of identifying key-player nodes that, together, optimally diffuse something through a network or maximally disrupt or fragment a network when removed. It further extends the standard network centrality measures of degree, closeness, and betweenness (refer to [20] for a clear introduction and to [21] for further theoretical explanations) to groups rather than individual elements. To this regard, these methods are a direct generalization of the corresponding individual measures, in such a way that if, e.g., group degree and degree are applied to groups consisting of single elements, they yield identical results. The classes of algorithms are thus two: one that measures the importance of a set on the basis of its impact on the remaining nodes of a network and another that does it by considering the sole properties of the elements of a set.

The former class is composed by the DF (KPP-Neg; cf. Eq. 2 in Methods), DR (cf. Eq. 3), and m-reach (KPP-Pos; cf. Eq. 4) algorithms [10]. KPP-Neg measures the fragmentation of a network because of a set. KPP-Pos measures the overall cohesion that members of a set have with the remainder of the network. As described in the Methods section, DF measures the degree of reachability of a set of nodes, taking also into account the degree of cohesion of the set. m-reach counts the number of unique nodes reached by any member of a set in *m* links or fewer. DR is the weighted proportion of all nodes reached by the set, where nodes are inversely weighted by their minimum distance from the set.

The latter class is formed by the group-degree centrality measure, which accounts for the number of non-group nodes that are connected to group members (cf. Eq. 5 in Methods); the group-betweenness centrality measure, which measures the proportion of (shortest) paths connecting pairs of non-group members that pass through the group (cf. Eq. 6); and the group closeness, which sums the distances from the group to all vertices outside the group (cf. Eq. 7).

#### Search strategies for optimal sets

When the aim is not to quantify the centrality of a specific set of nodes but that of discovering which is/are the most central set(s) in a network, search heuristics might come in handy. In particular, Pyntacle implements a “greedy optimization" search heuristics presented in [10] and a brute-force combinatorial optimization search strategy (cf. Search algorithms section in Methods). The former progressively replaces the components of a starting random set with all other nodes of a graph, calculating one of the aforementioned centrality metrics for that group, and then stops when a suboptimal solution is obtained. The latter loops through all possible groups of a predefined size and returns only those exhibiting the best scores for any of the centrality measure. It is immediate that the computational complexity of the heuristic method is much lower than that of the exact method, at the cost of suboptimal solutions. The brute-force search yields exact solutions but is computationally impracticable for big networks. The choice of a heuristic approach is due to its scalability to large-scale networks, while exact solutions are provided for smaller biological networks, for which there is no significant computational burden. It has to be noted that more efficient search strategies for large networks exist: Integer Linear Programming for exact solutions or metaheuristic approaches, such as population-based incremental learning methods [13].

#### Exploration of cross-talk pathways of sparse real-world networks

Real-world biological networks exhibit hierarchical organizations, where subnetworks (e.g., signaling pathways) are bridged by cross-talk links [22]. A number of developmental processes rely on cross-talk, where their aberrant regulation has been found to be associated with inflammatory response defects, as well as cancer and neurodegeneration [23, 24]. Together with the observation that causal molecular pathways often coincide with the shortest molecular paths between known disease-associated components (cf. the network parsimony principle [1]), these render the study of cross-talk in networks fundamental. Pyntacle eases the exploration of cross-talk by set operations on graphs. Individual networks can thus be compared (union, intersection, and difference) or merged and then studied topologically.

These networks are typically sparse and can be analyzed using algorithms that work best with graphs with a few edges. Pyntacle is optimized to work with increasingly large and complex networks. It lets the user assess the extent of sparseness of a network though mathematical indices, including the compactness and completeness indices [25, 26]. In addition, it chooses the best implementation of computationally heavy algorithms at run-time (e.g., the search for all the shortest paths), according to the available hardware (i.e., single or multi-core processors and GPU-enabled graphics cards) and some network global metrics, including the sparseness.

#### Data format compatibility and reporting

Pyntacle is compliant with the Cytoscape SIF data format and with the dot network data format. It can input and output adjacency matrices and edge lists as textual files, as well as serialized binary Python objects. Graph, node, or edge attributes can be imported/exported from/to file.

Pyntacle can report any analysis result in 2 formats: as textual files and as rich HTML files. In particular, the PyntacleInk module outputs an interactive, automatically generated web page that displays the graph, its attributes, and all the results of the analyses that were performed on it.

### Implementation

Pyntacle is accessible via command line and exposes a Python API for fine-tuning its algorithms. It depends on iGraph [17] for handling the graph data structure and borrowing some basic local and global topological measures and network generators.

Heavy computations of new algorithms are just-in-time compiled to native machine instructions by Numba [27] and thus run on multi-process CPU or NVIDIA-compatible GPU hardware, if available in the hosting computing infrastructure (experimental feature only accessible through APIs in version 1.3). Differently from similar packages, this allows Pyntacle to process graphs with thousands of nodes, thus helping it manage, for example, the whole human transcriptome and other networks of comparable sizes. Moreover, GPU acceleration provides high-speed computing of Pyntacle’s algorithms, thereby making heavy and long-running tasks feasible.

The PyntacleInk visualizer exploits HTML5, Javascript, and Sigma to produce an interactive representation of the input graph, its base metrics, and a graphic rendering of the results of most of Pyntacle’s algorithms (KPP, group centrality, graph generation, set operations, community detection, Fig. 6B). A graph can be displayed using different layouts (i.e., Random,Circular,ForceAtlas,Fruchterman-Reingold), and the canvas renderer enables visualization and smooth interaction with graphs ≤5,000 nodes in size, using a web browser of a standard desktop PC. All the information about a graph and the analyses that were performed on it are stored in a JSON file; this dictionary is updated with new information whenever a new run of analysis is performed on the same graph, allowing the user to simultaneously explore the results of different algorithms and—through the use of timestamps—the results of the same algorithm run with different parameters over time. Any graphical representation can be exported as vector graphics (SVG) or PNG screenshots.

Finally, Pyntacle is fully compatible with Jupyter Notebook.

### Benchmarks

Compared with the keyplayer 1.0.3 R package [28] and KeyPlayer 1.44 [29], Pyntacle has the following attributes: (i) is available for Windows, Mac, and Linux OS; (ii) is available as both command line tool and API; and (iii) allows management of real-world graphs in a computationally efficient way.

**Figure 4:**
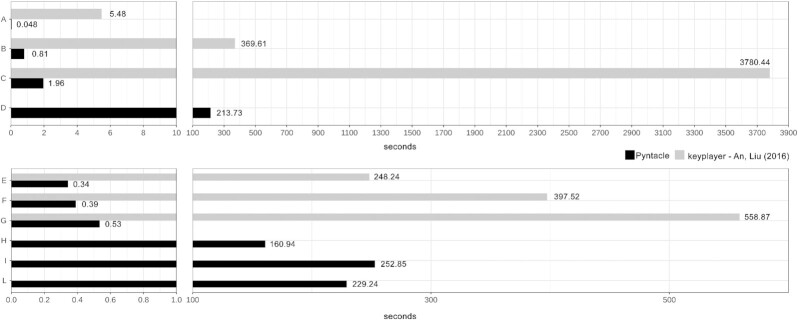
Greedy optimization search, metrics: DR. (A) Strong advice-seeking ties in global consulting company [10]; (B) parasite-host food web of the Carpinteria Salt Marsh Reserve [15]; (C) *C. elegans* connectome; (D) high-quality *C. elegans* protein-protein interaction network (APID); Erdős–Rényi random networks with (E–G) 100 nodes and rewiring probability *p* = 0.3, 0.5, and 0.7; (H–L) 1,000 nodes and *p* = 0.3, 0.5, and 0.7.

Wall-clock time comparisons of Pyntacle and keyplayer, when searching for optimal kp-sets of some real and simulated graphs, are shown in Fig. 4. Noteworthy is that KeyPlayer is not rigorously testable here because it is a Windows-only GUI-based application.

Random networks were generated according to the Erdős–Rényi model. Six random networks, 3 with 100 nodes and 3 with 1,000 nodes, were generated. These 2 groups of networks differed for their wiring probability, which varied as 0.3, 0.5, and 0.7. This probability is a kind of weighting function, which ranges from 0 to 1, with bigger numbers producing denser networks. Four other real networks were used: the network representing strong advice-seeking ties in a global consulting company [10] (32 vertices and 55 edges); the parasite-host food web of the Carpinteria Salt Marsh Reserve (128 vertices and 1,198 edges) [15]; the *Caenorhabditis elegans* connectome (a modified version of the network published in [30], 279 vertices and 1,960 edges); and a high-quality *C. elegans* protein-protein interaction network (3,303 vertices and 5,561 edges, downloaded from the Agile Protein Interactomes DataServer [APID] [31, 32]).

Wall-clock times were measured 3 times for each network and centrality algorithm. DR, m-reach, and DF were the only 3 algorithms in common between the 2 software packages. The suboptimal sets of size 2 were determined by both software packages using their own implementations of the greedy optimization search algorithm (cf. Search algorithms in the Methods section). Starting from the 100-node random networks, Pyntacle computed all indices in fractions of seconds (or a few seconds for DF), irrespective of the wiring probability. keyplayer computed the same indices of the same networks in 4–9 minutes. Considering the 1,000-node networks, keyplayer completed the computation of all indexes in >1 day, while Pyntacle took a few minutes to 5 hours (DF). Similarly, real networks were analyzed in fractions (or tenths for DF) of seconds by Pyntacle and in a few seconds to 1 hour by keyplayer, which took >1 day to analyze the APID network, as opposed to Pyntacle, which ran for a few minutes to 17 hours. Generally, Pyntacle was 40–3,900 times faster than keyplayer, depending on the test.

**Figure 5:**
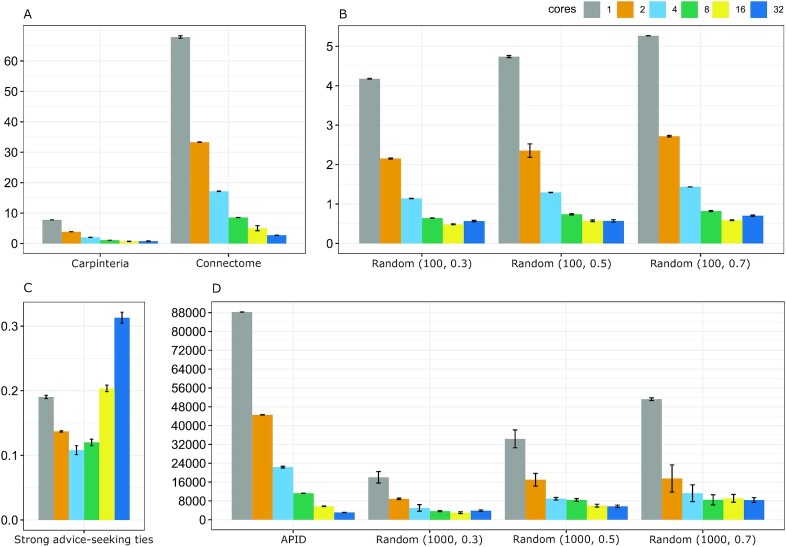
Improvement of execution times (in seconds) of Pyntacle using parallel computing on different networks using increasing numbers of computing cores. Bars represent mean and SD values.

The brute-force search algorithm yields exact solutions at the cost of an intrinsic combinatorial complexity. However, its computational load can be split into parallel processors. In Pyntacle, the best solutions are obtained after the enumeration of all possible groups of nodes and the calculation of their topological indices. Calculations are in fact independent from each other and hence suitable to being executed in parallel. When applied to our test networks with the aim of calculating the DR index, we verified that the smaller ones (≤100 nodes) have benefited from parallel execution only to a limited extent. While the strong advice-seeking ties in global consulting company network exhibited the best speedup with the use of 4 computing cores (1.76×), before decreasing its performance, the net execution time improvement consisted in fact of only 82 milliseconds, on average (Fig. 5C). Similarly, 100-node random networks, proportionally to the rewiring probability, achieved the best speedup values with 16 cores (~8×), with an improvement of just ~4 seconds (Fig. 5B). As expected, bigger networks benefited from parallel execution increasingly with the number of nodes. The Carpinteria network achieved the best speedup record (~11×) with 16 cores, although saving just 7 seconds of computation, while the connectome peaked at ~25× with 32 cores (Fig. 5A). The computations of 1,000-node random networks scaled well up to 16 nodes, exhibiting comparable speedups of ~7×, ~6×, and ~6× when the rewiring probability was varied from 0.3 to 0.5 and 0.7, respectively. The bigger APID network exhibited the best performance with 32 cores, achieving a speedup of ~29× and terminating the computation 23 hours earlier than the non-parallel run (Fig. 5D).

Although these are far from being linear speedups, the advantage and efficacy of parallel computing strategies is evident for big networks. These results can be reproduced using a Docker image available from the Pyntacle website.

## Analyses

### Case Study 1—protein-protein interaction interface

**Figure 6:**
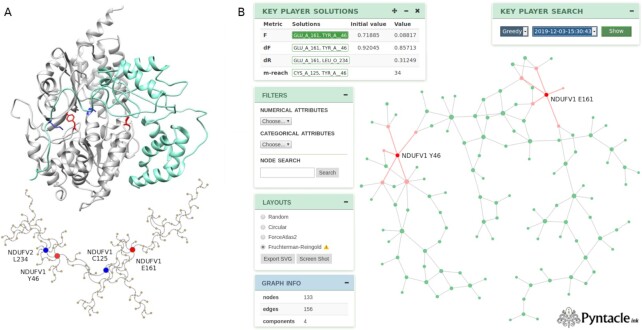
A: Representation of the interaction between NDUFV1 (white) and NDUFV2 (cyan) (PDB id 5xtd). The residues forming the interaction interface are represented as a graph (bottom) connecting residues close in space. Positive and negative key players are colored in blue and red, respectively, in both the interaction structure and interaction network. B: The PyntacleInk viewer. Different menus can be used to (i) visualize all the analyses that have been performed on a network, (ii) visualize the network general metrics, (iii) filter nodes by attributes, and (iv) change the overall layout of the network.

NADH dehydrogenase [ubiquinone] flavoproteins 1 and 2 (NDUFV1 and NDUFV2) are 2 core subunits of the mitochondrial respiratory Complex 1 [33]. Their interaction is mediated by 138 interface residues (Fig. 6A).

We have built a network whose edges linked interacting residues of the 2 proteins with the aim of identifying key residues at the interface between the 2 proteins and whose mutations might significantly affect their interaction (Fig. 6B). Thus, we computed several local topological metrics for these residues, e.g., degree, betweenness, closeness, radiality, and a few others, but none of them were shown to correlate appreciably with the contribution provided by each residue (Supplementary Fig. S1) on the NDUFV1-NDUFV2 interaction energy (ΔΔ*G*, expressed in kcal/mol and calculated with FoldX [34], see Methods): a maximum Pearson correlation of 0.32 was observed between ΔΔ*G* and betweenness.

We then applied Pyntacle to the network, searching for the best positive and negative key-player sets of size 2 (colored in blue and red, respectively, in Fig. 6, Supplementary Data S4). The residues Glu161 and Tyr46 of NDUFV1 were identified as the best negative key players in the network (according to both F and DF metrics), namely, their removal was estimated to maximally fragment the network and thus potentially hamper the interaction between the 2 proteins. This was further confirmed by their energetic contributions to the interaction when mutated to alanine (ΔΔ*G* +0.9 and +3.7 kcal/mol, respectively, for Glu161 and Tyr46). Moreover, Glu161 of NDUFV1, paired with Leu234 of NDUFV2, and Cys125 of NDUFV1, paired with Tyr46, were identified as the best positive key-player pairs (respectively calculated with the DF and m-reach metrics), namely, they resulted in being immediately reachable from the remaining network by direct links or indirect links joining close neighbor residues.

Contrary to Glu161 and Tyr46, Leu234 and Cys125 neither exhibited a significant ΔΔ*G* when mutated to alanine nor were characterized by high values of local topological metrics. For these reasons, they would have been overlooked by all other techniques, even the more accurate and computationally intensive, such as alanine scanning [35]. Other than being computationally demanding and impracticable for large-scale analyses, alanine scanning is known to be blind to residues that are chemically similar to alanine, thereby ignoring their epistatic features, which are critical in some regions of the interaction interface.

All these issues are overcome with Pyntacle, which makes it possible to look for topologically important groups of residues between proteins efficiently and regardless of their chemical structure.

### Case Study 2—miRNA-miRNA interaction network

MicroRNAs (miRNAs) are small RNA molecules (18–25 nucleotides) able to regulate gene expression levels through different cellular mechanisms, the most important of which is that a miRNA can recognize different messenger RNAs as targets and, at the same time, one of those targets can be recognized by multiple miRNAs. Owing to the renowned role played by miRNAs in tumorigenesis and cancer progression [36], we have analyzed a miRNA interaction network of patients affected by breast cancer.

Expression data of healthy and tumor tissue samples of 87 patients affected by breast cancer were retrieved from The Cancer Genome Atlas (TCGA) and were used to wire 487 miRNAs in 2 correlation networks, built on healthy and tumor samples, respectively. A functional association between any 2 miRNAs was assumed to exist if the absolute values of the Pearson correlation coefficients of their expression levels exceeded 0.5 (cf. Case Study 2 in Methods).

The healthy network was very dense and highly connected (average degree of 40.4), as opposed to the tumor network, which was mostly disconnected (average degree of 17.4). Both networks were analyzed with Pyntacle, which identified miR-1307-3p and miR-140-3p as negative key players and miR-136-5p, miR-484, and miR-127-5p as positive key players of the healthy tissue network. These are all associated with the onset and development of breast cancer and some are even used as markers for prognosis [37–42] (Supplementary Data S5).

In the tumor network, Pyntacle identified miR-192-5p, miR-483-5p, and miR-577 as negative key players and miR-324-5p and miR-337-3p as positive key players, all involved in proliferation, cell migration, and metastasis of breast cancer [43–47] (Supplementary Data S6). It has to be noted that while these miRNAs individually have high betweenness values, it would not have been possible to infer a possible synergistic interaction between them without the key-player analysis of Pyntacle.

## Discussion and Concluding Remarks

The motivations behind Pyntacle come from the constant growth of experimental data sets and from the increasing need to represent and analyze them with computationally efficient methods. This first release of Pyntacle has been designed with these aims and thus with the intent to help researchers from different scientific fields and with different levels of computing skills to approach network biology and benefit from its analytical tools.

We showed the attributes of Pyntacle and its versatility in dealing with different problems, all of which are traceable to possibly big networks of interactive elements. In one case study, Pyntacle was thus able to identify key amino acids that were greatly contributing to the formation of a protein-protein interaction interface. This otherwise time-consuming task was accomplished very efficiently by translating the problem into a network analysis task, compared with current approaches that take tens of minutes to handle even small interaction interfaces. Another similar and detailed case study can be found in [48]. In a second case study, Pyntacle was used to analyze the TCGA data set of miRNA expression in breast cancer to build miRNA-miRNA networks. These were analyzed in search of miRNAs that were occupying key positions in the network, which were later recognized as already known biomarkers or responsible for the onset and progression of breast cancer.

In conclusion, Pyntacle represents a starting point for large-scale network biology studies. Being a modular framework, it will be expanded to handle weighted networks, in the near future, and directed networks, immediately after. These features, which are shared with some other key-player detection methods and tools [28, 49, 50], are relevant to make Pyntacle fully capable of analyzing all kinds of biological networks. It will also be enriched with new optimization search algorithms and with a new algorithm to compute the set nestedness. New applications and use-cases are envisaged, the currently most concrete being one concerning the analysis of trajectories of molecular dynamics simulation of proteins.

## Potential Implications

The main problem concerning most of the currently available network analysis tools, which is also the main reason why we made Pyntacle, is that these do not handle networks of medium to big sizes efficiently. This issue not only relates to the big networks and the suitability and effectiveness of the currently available algorithms to analyze them but also their practicability over a reasonable time axis and in terms of required computing resources. This point is critical for most fields of research, from biology to medical and social sciences, where systems are naturally big and complex (e.g., the whole proteome, the diseasome, and socialnomics, to mention a few). In particular, a field that at the time of this writing is hitting the headlines, i.e., epidemiology, is greatly developing in terms of the capability to draw contagion maps and predict infection growth over time. These maps are actually networks where nodes are people and edges are relationships that occurred in recent and short periods of time. There are many ways these networks could be studied. One way could be determining the leading front of the infection, namely, a group composed of healthy people that are close to the affected people and highly social and to administer a vaccine to curb the infection. Another possibility would be that of determining the minimum possible number of communication routes to be closed at a national level to implement a clever lockdown. These and several other options may be implemented in Pyntacle using ad hoc algorithms and computing protocols tailored for big networks.

## Methods

### Key-player and group-centrality metrics

Pyntacle tackles the problem of identifying key-player nodes that, together, optimally diffuse something through a network or maximally disrupt or fragment a network when removed. The classes of algorithms are thus two: one that measures the importance of a set on the basis of its impact on the remaining nodes of a network and another that does it by considering the sole properties of the elements of a set. The former class, also known as KPP-Neg, measures the fragmentation of a network because of the removal of a set of nodes. It is composed by the *F* metrics:

(1) \begin{equation*} F=1 - \frac{\sum _{k} s_{k}(s_{k}-1)}{n(n-1)} , \end{equation*}

which are based on the size *s_k_* of its components *k*; and by the DF metrics:

(2) \begin{equation*} \mathrm{DF}=1 - \frac{2\sum _{i> j} (1/d_{ij})}{n(n-1)} , \end{equation*}

where *d_ij_* denotes the distance between the *i*th and the *j*th node. It ranges from 1, when all nodes are adjacent, to 0, when all nodes are isolates.

The latter class, also known as KPP-Pos, measures the overall cohesion that members of a set have with the remainder of the network and is made up of:

(3) \begin{equation*} \mathrm{DR}=\frac{\sum _j (1/d_{Kj})}{n} , \end{equation*}

where *n* is the size of the graph and *d_Kj_* denotes the minimum distance (shortest path) between any member *i* of the set *K* and the remaining nodes *j* in the graph. Similarly, the m*-*reach metric counts how many unique nodes can be reached from *K* in *m* steps or less. The formulation is:

(4) \begin{equation*} C_K = \sum\nolimits _{j\in V\setminus K} \cup \frac{1}{d_{ij}}, \end{equation*}

where *C_K_* ranges from 0 to *n* − *k, n* representing the size of the graph and *k* that of the considered set of nodes. It is important to notice that this index assumes that all paths of length *m* or less are equally important and that all paths longer than *m* are wholly irrelevant [10].

Pyntacle further extends the standard network centrality measures of degree, closeness, and betweenness to groups of nodes, in a way that if, e.g., group-degree and degree centrality measures are applied to groups consisting of single elements, they yield identical results. This class of metrics is formed by the group-degree centrality measure, which is intended as the number of non-group nodes that are connected to group members. Multiple ties to the same node are counted only once. It is defined as follows:

(5) \begin{equation*} \mathrm{GD}_K=\frac{\sum \nolimits _{i\in K, j\in {V\setminus K}} a_{ij}}{\Vert V\setminus K\Vert } , \end{equation*}

where *a_ij_* = 1 when *i* and *j* are adjacent nodes, if *i* ∈ *K* and *j* ∈ {*V*∖*K*}, and counting *a_i, j_* and *a_v, j_* only once ∀*v* ∈ *K* when *a_i, j_* = 1 and *a_v, j_* = 1. Hence, the group-degree centrality ranges from 0 to 1 if the group *K* is completely isolated or fully connected to all other nodes. Group-betweenness centrality of a set *K* is defined as the number of shortest paths connecting any 2 nodes *u* and *v* passing through *K* over the number of all paths between the two.

(6) \begin{equation*} \mathrm{GB}_K=\frac{p_{u,v}(K)}{p_{u,v}} , \end{equation*}

where *u* and *v* are any pair of nodes not belonging to the group *K, p_u, v_*(*K*) represents the number of shortest paths connecting *u* and *v* and that traverses *K*, while *p_u, v_* is the total number of shortest paths between *u* and *v*. Group closeness of a group *K* is defined as the sum of the minimum,maximum,and average distances from the nodes belonging to the group to all other nodes outside the group.

(7) \begin{equation*} \mathrm{GC}_K = \frac{\sum _{j\in {V\setminus K}} \bar{d}_{Kj}}{\Vert V\setminus K\Vert } , \end{equation*}

where $\bar{d}_{ij}$ is the minimum, maximum, or average distance between nodes in *K* and all other nodes.

### Search algorithms

When the aim is not to quantify the centrality of a specific set of nodes but to discover which is/are the most central set/s of nodes in a network, search heuristics might come in handy. In particular, Pyntacle implements a greedy optimization search heuristics presented in [10], and a brute-force combinatorial optimization search strategy. The former follows this naive algorithm:

**Algorithm 1:**
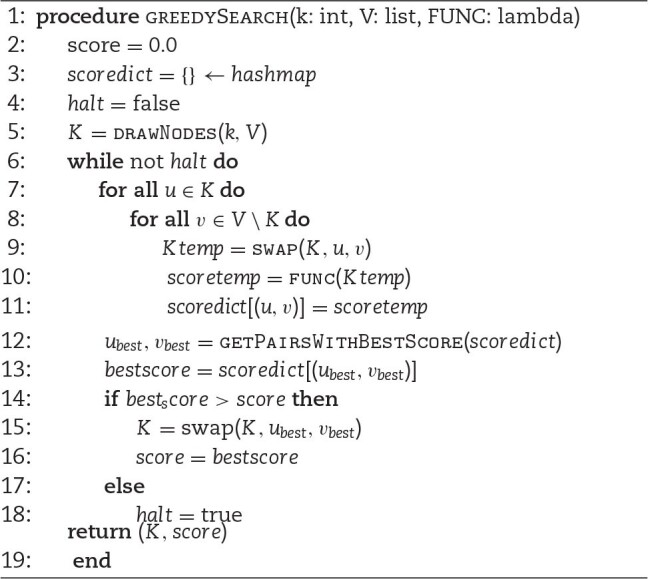
Greedy optimization search

FUNC is an appropriate key-player metrics; “drawnodes" is a function that randomly picks *k* nodes from *V*, which contains all nodes of the network; “swap(*K,u,v*)" substitutes the element *u* in *K* with *v*; “getPairsWithBestScore" is a function that returns the pairs that yielded the best centrality score. This method progressively replaces the components of a starting random set *K* with all other nodes of a graph, calculating one of the aforementioned centrality metrics for that group and then stopping when a suboptimal solution is obtained.

The brute-force combinatorial optimization search strategy implemented in Pyntacle loops through all possible groups of a predefined size (*k*) and returns only those exhibiting the best scores for any of the previous centrality measure (*F*). Even if the computation can be performed in parallel (the “for all" loop below), it is obvious that the computational complexity of the heuristic method is much lower than that of this method, at the cost of yielding suboptimal solutions. The algorithm below returns exact solutions but is computationally impracticable with big networks.

**Algorithm 2:**
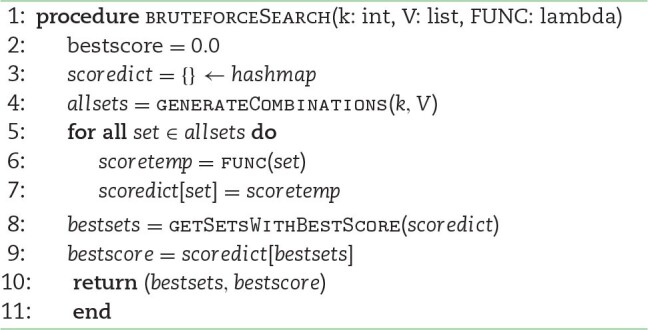
Brute-force search

generateCombinations is a function that generates all possible sets of nodes of size *k* picking nodes from *V*.

### Set operations on graphs

Graph union, *G*1∪*G*2, is implemented as (*V*1∪*V*2, *E*1∪*E*2), namely, as the union of nodes (*V*) and edges (*E*). Graph intersection is defined as *G*1∩*G*2 = (*V*1∩*V*2, *E*1∩*E*2), where only common nodes and edges are reported in the resulting graph. The difference between *G*1 and *G*2 results in a graph with nodes and edges only present in *G*1 and not in *G*2. Because the difference between graphs is not reciprocal, *G*1 − *G*2 ≠ *G*2 − *G*1.

### Case Study 1

Chains *A* and *O* of the Protein Data Bank (PDB) structure 5xtd were considered for the analysis of NDUFV1 and NDUFV2, respectively. First, the structure has been repaired (RepairPDB module of FoldX) and thus allows more relaxed residue side-chain rotamers and the solution of clashes. Then, residues located at the interaction interface and the interaction energy were determined with the AnalyseComplex module of FoldX. Alanine scanning of the interface residues was performed by substituting each amino acid with an alanine residue (BuildModel module). The interaction energy of each mutant has been calculated with the AnalyseComplex module to determine the ΔΔ*G* of the mutant compared to the wild-type interaction structure. A residue-residue interaction network was built, which connected residues belonging to different chains in the complex if any of their non-hydrogen atoms (both backbone and side-chain) were within a 4.5 Å radius from each other. The network was then analyzed with the keyplayer module of Pyntacle and a greedy search algorithm was used to find the optimal key-player sets of size 2, considering all available metrics (F and DF, as KPP-Neg; and DR and m-reach, with *m* set to 2, as KPP-Pos).

### Case Study 2

Expression levels of 547 miRNAs in 87 healthy and 87 tumor breast samples were retrieved from TCGA. Separately for healthy and tumor individuals, correlations of expression between any possible pairs of miRNAs (149,331 total pairs) were calculated by Pearson correlation coefficient. Only significant values that exceeded ±0.5 were considered to represent edges connecting miRNAs in the healthy and tumor networks. In both networks, the best KPP-Pos and KPP-Neg sets of size 2 were sought using Pyntacle’s greedy optimization search and calculating F and DF, as KPP-Neg; and DR and m-reach, with *m* set to 1, as KPP-Pos.

## Availability of Source Code and Requirements

- Project name: Pyntacle
- Project home page:  http://pyntacle.css-mendel.it
- Operating systems: Linux,Mac, and Windows
- Programming language: Python 3.7+
- Other requirements: CUDA toolkit (optional)
- License: GNU GPL 3.0
- RRID:SCR_019030
- bio.tools ID: biotools:pyntacle

Source code is stored in GitHub. Installation procedures, tutorials, case studies, and a Docker container are all available from the Pyntacle website.

## Availability of Supporting Data and Materials

An archival copy of the supporting data, for the reproduction of the case studies, is also available via the GigaScience repository, GigaDB [51].

## Additional Files

Supplementary Figure S1.

Supplementary Data S1. Centrality metrics calculated for the network in Figure 1

Supplementary Data S2. Nestedness of the best sets by group-centrality metrics for the network in Figure 1

Supplementary Data S3. Nestedness of the best KP sets for the network in Figure 1

Supplementary Data S4. Local topological metrics and ∆∆G values of the residues represented in Figure 6A

Supplementary Data S5. Local topological metrics for the “healthy" network described in Case Study 2

Supplementary Data S6. Local topological metrics for the “tumor" network described in Case Study 2

## Abbreviations

API: Application Programming Interface; APID: Agile Protein Interactomes; CPU: central processing unit; GPU: graphics processing unit; GUI: graphical user interface; KPP-Neg: Key-Player Problem/Negative; KPP-Pos: Key-Player Problem/Positive; miRNA: microRNA; NADH: nicotinamide adenine dinucleotide–hydrogen (reduced); PDB: Protein Data Bank; TCGA: The Cancer Genome Atlas.

## Competing Interests

The authors declare that they have no competing interests.

## Funding

This study was supported by the Italian Ministry of Health (Ricerca Corrente 2018-2020) and by the “5 × 1000” voluntary contribution.

## Authors' Contributions

L.P. designed and performed the experiments; M.T. implemented PyntacleInk and took care of the package maintenance and deployment; D.C. wrapped the iGraph modules and performed the benchmarks; T.B., S.C., and F.P. contributed to the case study analysis; M.C. contributed to the definition of the case studies; F.J. oversaw the implementation of the topology metrics; T.M. designed and implemented the software and oversaw the project.

## Supplementary Material

giaa115_GIGA-D-20-00087_Original_Submission

giaa115_GIGA-D-20-00087_Revision_1

giaa115_GIGA-D-20-00087_Revision_2

giaa115_GIGA-D-20-00087_Revision_3

giaa115_GIGA-D-20-00087_Revision_4

giaa115_Response_to_Reviewer_Comments_Original_Submission

giaa115_Response_to_Reviewer_Comments_Revision_1

giaa115_Response_to_Reviewer_Comments_Revision_2

giaa115_Response_to_Reviewer_Comments_Revision_3

giaa115_Reviewer_1_Report_Original_Submission Alexander Stivala, PhD -- 4/12/2020 Reviewed

giaa115_Reviewer_1_Report_Revision_1 Alexander Stivala, PhD -- 7/14/2020 Reviewed

giaa115_Reviewer_2_Report_Original_Submission Karthik Raman -- 4/28/2020 Reviewed

giaa115_Reviewer_2_Report_Revision_1 Karthik Raman -- 7/17/2020 Reviewed

giaa115_Supplemental_Files
