# Supplementary material for: Pyntacle: a parallel computing-enabled framework for large-scale network biology analysis
Source: Gigascience. 2020 Oct 21;9(10):giaa115. doi: 10.1093/gigascience/giaa115 (PMC7576925; doi:10.1093/gigascience/giaa115)
Supplement: giaa115_GIGA-D-20-00087_Original_Submission [file giaa115_giga-d-20-00087_original_submission.pdf]

GigaScience

Pyntacle: a parallel computing-enabled framework for large-scale network biology analysis

--Manuscript Draft--

**Manuscript Number:**	GIGA-D-20-00087
**Full Title:**	Pyntacle: a parallel computing-enabled framework for large-scale network biology analysis
**Article Type:**	Technical Note
**Funding Information:**	
**Abstract:**	Some natural systems are big in size, complex and often characterized by convoluted mechanisms of interaction, such as epistasis, pleiotropy, and trophism, which cannot be immediately ascribed to individual natural events or biological entities, but that are often derived from group-effects. However, the determination of important groups of entities, like genes or proteins, in complex systems is considered a computationally hard task. Here, we present Pyntacle, a high-performance framework designed to exploit parallel computing and Graph Theory to efficiently identify critical groups in big networks and in scenarios that cannot be tackled with traditional network analysis approaches. We showcase potential applications of Pyntacle with transcriptomics and structural biology data, thereby highlighting the outstanding improvement in terms of computational resources over existing tools.
**Corresponding Author:**	Tommaso Mazza, Ph.D.<br>IRCCS Casa Sollievo della Sofferenza<br>Roma, Please Select ITALY
**Corresponding Author Secondary Information:**	
**Corresponding Author's Institution:**	IRCCS Casa Sollievo della Sofferenza
**Corresponding Author's Secondary Institution:**	
**First Author:**	Luca Parca, Ph.D.
**First Author Secondary Information:**	
**Order of Authors:**	Luca Parca, Ph.D.
	Mauro Truglio
	Tommaso Biagini
	Stefano Castellana
	Francesco Petrizzelli
	Daniele Capocefalo
	Ferenc Jordán
	Massimo Carella
	Tommaso Mazza, Ph.D.
**Order of Authors Secondary Information:**	
**Additional Information:**	
**Question**	**Response**
Are you submitting this manuscript to a special series or article collection?	No
**Experimental design and statistics**	Yes

Powered by Editorial Manager® and ProduXion Manager® from Aries Systems Corporation

Full details of the experimental design and statistical methods used should be given in the Methods section, as detailed in our Minimum Standards Reporting Checklist. Information essential to interpreting the data presented should be made available in the figure legends.<br><br>Have you included all the information requested in your manuscript?	
**Resources**<br><br>A description of all resources used, including antibodies, cell lines, animals and software tools, with enough information to allow them to be uniquely identified, should be included in the Methods section. Authors are strongly encouraged to cite Research Resource Identifiers (RRIDs) for antibodies, model organisms and tools, where possible.<br><br>Have you included the information requested as detailed in our Minimum Standards Reporting Checklist?	Yes
**Availability of data and materials**<br><br>All datasets and code on which the conclusions of the paper rely must be either included in your submission or deposited in publicly available repositories (where available and ethically appropriate), referencing such data using a unique identifier in the references and in the "Availability of Data and Materials" section of your manuscript.<br><br>Have you have met the above requirement as detailed in our Minimum Standards Reporting Checklist?	Yes

The main manuscript file

Click here to download Manuscript Pyntacle_GIgaScience_2020.pdf

OXFORD (GIGA)^n SCIENCE

GigaScience, 2020, 1–11

doi: xx.xxxx/xxxx
Manuscript in Preparation
Technical Notes

TECHNICAL NOTES

Pyntacle: a parallel computing-enabled framework for large-scale network biology analysis

Luca Parca[1], Mauro Truglio[1], Tommaso Biagini[1], Stefano Castellana[1], Francesco Petrizzelli[1], Daniele Capocefalo[1], Ferenc Jordán[2], Massimo Carella[3] and Tommaso Mazza[1,*]

[1]IRCCS Casa Sollievo della Sofferenza, Laboratory of Bioinformatics, San Giovanni Rotondo (FG), Italy and [2]Danube Research Institute, MTA Centre for Ecological Research, Budapest, Hungary and [3]IRCCS Casa Sollievo della Sofferenza, Laboratory of Medical Genetics, San Giovanni Rotondo (FG), Italy

[*]t.mazza@css-mendel.it

Abstract

Some natural systems are big in size, complex and often characterized by convoluted mechanisms of interaction, such as *epistasis*, *pleiotropy*, and *trophism*, which cannot be immediately ascribed to individual natural events or biological entities, but that are often derived from group-effects. However, the determination of important groups of entities, like genes or proteins, in complex systems is considered a computationally hard task. Here, we present Pyntacle, a high-performance framework designed to exploit parallel computing and Graph Theory to efficiently identify critical groups in big networks and in scenarios that cannot be tackled with traditional network analysis approaches. We showcase potential applications of Pyntacle with transcriptomics and structural biology data, thereby highlighting the outstanding improvement in terms of computational resources over existing tools.

Key words: network; topology; group centrality; systems biology; parallel computing; python

Background

Interactive systems are commonly represented as graphs (or networks), which are mathematical representations of *elements* (nodes) and their relationships (edges). The semantics of relationships is specific for each graph and completely defines its expressiveness. Protein interaction networks, for example, represents physical interactions as edges and proteins as nodes; metabolic networks wire metabolites if these participate in the same biochemical reaction; regulatory networks are directed graphs, where the directionality of relationships matters. Thus, a link exists between two molecules if there is evidence either of regulatory activity by a transcription factor onto a gene or of post-translational modifications. These, together with several other kinds of networks, like RNA, signaling, neuronal, trophic, and co-expression networks, are the concrete signs of an exceptional growth of molecular interaction data and, hence, of an intense research activity in the field of *Network medicine* [1].

Network medicine is a relatively new discipline that exploits graph theory to identify key-molecules in the human *diseasome* [2] together with their hidden molecular relationships. The general aim is that of reverse-engineering the mechanisms of pathogenesis of complex disorders and traits, whereby the etiology is notoriously convoluted. The diseasome is, in fact, a network where diseases are nodes and links represent relationships between the disease-associated cellular components. Determining such links would help identify the molecular relationships between phenotypes, the reasons of certain comorbidities, and would positively affect diagnosis, treatment and drug multi-purposing.

Certain kinds of biological networks share the feature of having relatively few highly connected nodes, often called *hubs*, suggesting that the molecules represented by hubs should play special biological roles. The first hypothesis of Network

Compiled on: March 24, 2020.
Draft manuscript prepared by the author.

Key Points

- Understanding the architecture of networks as well as the key-roles of their components is a process that historically relies on the calculation of sets of global and local topological indices.
- The information provided by local topological indices is little or meaningless for natural networks of big sizes or for those that model complex events, like *epistasis*, *pleiotropy*, and *trophism*, which in fact result from the interleaving actions of multiple natural actors.
- Pyntacle provides the user with an array of computationally efficient algorithms to manage networks and to search and find *important* groups of nodes.

medicine is that a vast majority of known diseases genes, which are *non-essential*, lie in the periphery of these networks and are far from hubs. On the contrary, at least in human cells, hub molecules are encoded by essential genes [3]. A database, DEG, exists that reports essential genes for some bacteria, archaea and eukaryotes [4]. An interesting speculation is that, because of their many links, hubs are reasonably associated with *disease genes* [5, 6, 7], which in turn, by virtue of the *local hypothesis* of Network medicine, exhibit increased tendency to interact with each other, being them all involved in the same disease. Thus, molecular networks are not random, but tightly organized based on specific principles, according to which the effect of a *central* gene, which is eventually aberrant, reverberates on the gene products of neighboring genes in its network. Hence, the expression of a disease phenotype does rarely result from an individual aberrant gene, rather from the harmonized effects of groups of related genes. This holds true also for other types of networks, ranging from ecological, to evolutionary and chemical networks.

Graph theory draws upon various tools to identify the most central elements, i.e. the key molecules, in a network. Here, the concept of centrality is synonymous with importance, even if has been seen to decline differently in literature. A topologically important node may be a hub, a *bottleneck*, namely a node that lies in many pathways, or one that is "close" to most other nodes. But for what said above, local (i.e., regarding nodes or edges) and global (i.e., regarding the entire network) properties of networks are unlikely to completely explain the functioning of complex systems, since they do not take into account or underestimate the effects that groups of important nodes may jointly exert on these systems. Node 2 in Figure 1 has 7 ties and it is the highest *degree* node in this example network. It is connected with 7 unimportant nodes, since these exhibit low degree values. Node 9 is the second more connected node with only one less edge than node 2, but two of its neighbors, i.e., node 16 and 21, are the third and fourth ranked nodes by degree with 5 and 4 ties, respectively. Thus, although node 2 is top ranked by degree, it may not be the most functionally central node. Whether this assertion is true or not strictly depends on the purposes by which a network is being studied.

More interestingly, the *network parsimony* principle of Network medicine, according to which *causal molecular pathways often coincide with the shortest molecular paths between known disease-associated components*, implies that it is fundamental to find the nodes that lie within the highest number of pathways in networks, since these are more likely to be functionally critical [1]. The *betweenness* centrality index [8] is the most suitable for this task. Node 21 in Figure 1 is the top ranked node by betweenness. This was expected since it lies in the exact middle of the network, that in turn, exhibits a quasi-tree topological node organization. But even if node 21 belongs to almost all shortest paths of the network, it ranks only fourth by degree, since it is not individually much connected. Node 9 is the second node by betweenness with a score very close to that of node

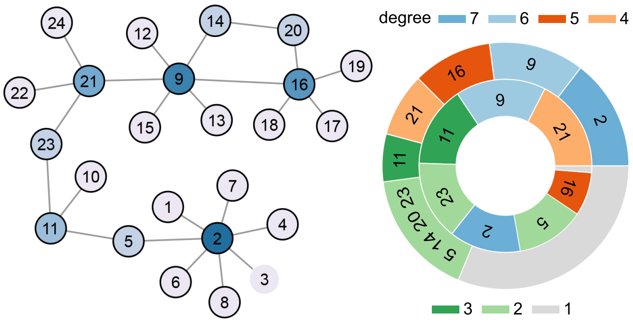

Figure 1. (left) Example network. The darker the blue color, the higher the degree of nodes. (right) Pie chart of the most central nodes. The outer circle reports the highest degree nodes (counterclockwise, blue through gray). The inner circle represents the highest betweenness nodes, from light orange to gray, counterclockwise. Node names are reported within circle sectors. Sector width is proportional to the degree (outer) and betweenness (inner) values of nodes. Gray sectors contain unimportant nodes, i.e. nodes with unitary degree and negligible betweenness values.

Table 1. Group centrality metrics calculated for the example network. Higher scores indicate higher centrality.

Group	Degree	Betweenness	Closeness*
{2, 21}	0.5	0.39	0.58
{2, 9}	0.59	0.43	0.69
{21, 9}	0.36	0.35	0.38
{2, 9, 21}	0.71	0.45	0.75

* The *minimum* method was used to measure the distance from the group to an outside node.

21 but, on the contrary, it ranks second by degree (Supplementary data S1). Whether the most important node is 2, 21 or node 9 depends on the aims and context of the study.

Whenever more than one node exhibits similar topological scores, as in this case or when a co-responsibility for a phenotype is suspected, studying groups and their centrality might be a reasonable option. In 1999, Everett and Borgatti expanded the definition of degree, betweenness and *closeness* to groups of nodes [9]. Calculating these indices for the following groups: {2, 21}, {9, 21}, {2, 9}, the latter achieved the highest scores. Moreover, considering the group made by all three nodes, only degree and closeness increased significantly in respect to {2, 9} (cf. Table 1).

In 2006, Borgatti introduced other two classes of metrics for groups that were meant to assess the ability of groups either to disrupt a network, when removed, or to efficiently spread *information* through a network. These were defined as “Key-Player Problem/Negative” (KPP-Neg) and “Key-Player Problem/Positive” (KPP-Pos), respectively [10]. KPP-Neg and KPP-Pos were calculated for the same groups and reported in Table 2. It is interesting to notice that {2, 9} is still the most important

group in terms of disruption potential and connectivity. Their scores were slightly lower than those of group {2, 9, 11}, meaning that even here node 11 does not contribute significantly to the centrality of {2, 9}.

Table 2. KPP-Neg and KPP-Pos metrics calculated for the example network. DF (Neg) achieves its maximum value of 1.0 when the graph consists entirely of isolated nodes. M-reach (Pos) is a count of the number of unique nodes reached by any member of the group in *m* links or less. DR (Pos) achieves a maximum value of 1 when every non-group node is adjacent to at least one member of the group.

Group	DF (0.66)	m-reach*	DR
{2, 21}	0.87	79.2%	0.65
{2, 9}	0.91	95.8%	0.72
{21, 9}	0.84	62.5%	0.53
{2, 9, 21}	0.93	95.8%	0.74

* The *m* parameter of the algorithm was set to 2. The percentage of nodes reached by the group, including the group nodes, is reported.

What remains to be verified is whether any other group exists that exhibits similar or higher centrality values. Considering the small network size, the option of running a *brute-force* algorithm to search the absolute best group(s) among all possible ones is computationally feasible, in place of a *greedy-optimization* search, as suggested by Borgatti in [10]. In this case, the best group of size 2 for all metrics is still {2, 9}, whereas {5, 9} reaches 100% of non-group nodes and ranked first by m-reach. However, since none of the centrality scores of {2, 9} equaled their maximum possible values, we applied again the brute-force search to groups of increasing sizes, 3 through 6. We thus found that degree and closeness reached their absolute maximum scores, i.e. 1, equally with two groups {2, 9, 11, 16, 21}, {2, 9, 10, 16, 21} of size 5, meaning that nodes 10 and 11 are interchangeable and equally important; betweenness obtained its maximum score (0.497) with the group {2, 9, 11, 16, 21} (Supplementary data S2). The best group by DF is {2, 9, 11, 14, 16, 21}, which achieves the score of 1. The groups {2, 9, 10, 16, 21} and {2, 9, 11, 16, 21} equally obtained the best DR score (0.792). It is worth noticing that DR and betweenness do not reach their absolute maximum scores, which however are plausibly the highest possible for this network, since groups of bigger sizes exhibit lower scores (Figure 2). It is also interesting to notice that nodes 2, 9 and 21 are included in all groups found above, thereby highlighting their central roles in the network (Supplementary data S3).

Computing the *nestedness*, which consists in verifying whether sets of nodes recur in groups of increasing sizes, could confirm the importance of nodes 2, 9 and 21. Hence, if larger sets contain smaller sets, higher values of nestedness may be a proxy for identifying upstream/master regulators through the key-nodes of the smallest groups. One way to calculate the nestedness of the example network is by the *Nrow* metrics [11, 12]. Nrow is defined as the average percentage of nodes from smaller sets that are contained in larger sets, taking all possible pairs of sets. Thus, after computing all the best sets of increasing sizes, from 2 to 5, for each group centrality metrics but *m-reach*, nodes 2 and 9, and not 21, resulted to be nested in all sets, regardless of their size (Figure 3 and Supplementary data S2). The same evidence emerged with the key-player metrics (Supplementary data S3). The nestedness scores were generally quite high, meaning that nodes are not interchangeable among groups, i.e., there are few equally important nodes. The group {2, 9} is definitely important from a topological point of view and its discovery would have not been immediately hypothesized without this investigation, since the nodes 2 and 9 are five links apart.

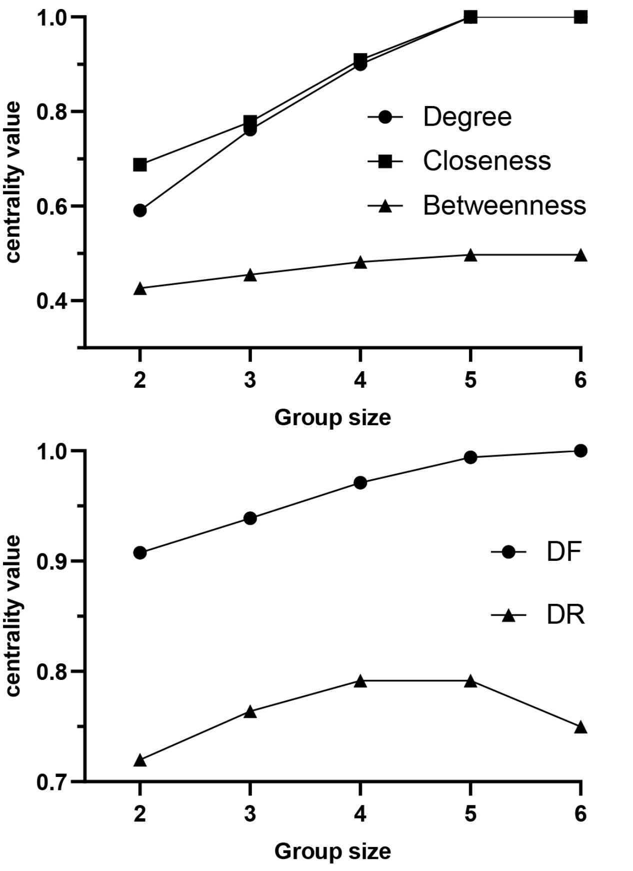

Figure 2. Brute-force search algorithm applied to all groups of sizes 2 through 6. For any size, the maximum score obtained for (up) group-degree, group-closeness, group-betweenness, (down) DF and DR are plotted.

This "practical" introduction aims at introducing the theory underlying Pyntacle. A toy-model was used to describe the main features, outline a possible analytical pathway and highlight how Pyntacle may help extract valuable information from real-world networks. The rest of the article will thus present i) the software and its main components, ii) its design and implementation, iii) how to finely use it; iv) benchmarks, assessed on real and simulated networks of increasing sizes, in comparison with a similar software package; v) two real-world case studies.

Pyntacle

Pyntacle is an open-source network analysis framework that was originally designed to tackle the Borgatti's Key-Player Problem [10] efficiently through the identification of maximally reachable or disruptive groups of nodes. Pyntacle adopts optimized heuristic algorithms and parallel computing strategies to make these tasks feasible. It has the following attributes: i) available for Windows, Mac, and Linux OS; ii) available as both command line tool and API, with an easy and user-friendly interface for both input commands and results visualization; iii) it allows the management of real-world graphs in a computationally efficient way.

Pyntacle is implemented in modules, each designed to analyze a particular aspect of a network. These can calculate global and local topological metrics (*metrics* module), the importance of groups of nodes (*groupcentrality* and *keyplayer* modules), search and analyze clusters of nodes (*communities* module), per-

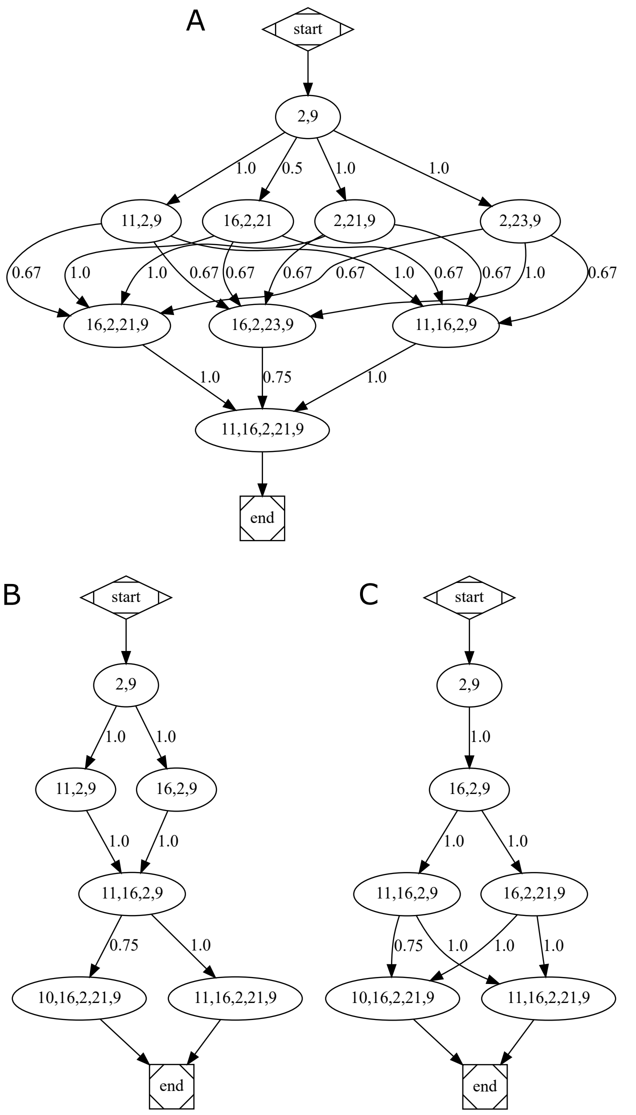

Figure 3. Nestedness graphs for group- (A) *betweenness*, (B) *closeness*, (C) *degree* centrality metrics. Nodes represent groups with top centrality values in respect to all other possible groups of nodes with same sizes. Edges connect groups when the bigger group contains at least one element of the smaller one. Edges are labeled with the overlap ratio between the elements of the connected groups.

form set operations between networks (*set* module), generate networks with different topological organizations (e.g. random, scale-free and small-world networks, *generate* module), convert and load/save networks using different data formats (e.g. adjacency matrix, edge list, SIF and dot, *convert* module).

Features

Centrality measures for groups

Pyntacle tackles the problem of identifying key-player nodes that, together, optimally diffuse *something* through a network or maximally disrupt or fragment a network when removed. It further extends the standard network centrality measures of *degree*, *closeness*, and *betweenness* (refer to [13] for a clear introduction and to [14] for further theoretical explanations) to groups rather than individual elements. To this regard, these methods are a direct generalization of the corresponding individual measures, in a way that if, e.g., *group-degree* and *degree* are applied to groups consisting of single elements, they yield identical results. The class of algorithms are thus two: one that measures the importance of a set on the basis of its impact on the remaining nodes of a network, and another that does it by considering the sole properties of the elements of a set.

The former class is composed by the DF (KPP-Neg; cf. Eq. 1 in Methods), DR (cf. Eq. 2) and m-reach (KPP-Pos; cf. Eg. 3) algorithms [10]. KPP-Neg measures the fragmentation of a network because of a set. KPP-Pos measures the overall cohesion that members of a set have with the remainder of the network. As described in the Methods section, DF measures the degree of *reachability* of a set of nodes, taking also into account the degree of cohesion of the set. M-reach counts the number of unique nodes reached by any member of a set in *m* links or less. DR is the weighted proportion of all nodes reached by the set, where nodes are inversely weighted by their minimum distance from the set.

The latter class is made by the *group-degree* centrality measure that accounts for the number of non-group nodes that are connected to group members (cf. Eq. 4 in Methods); the *group-betweenness* centrality measure that measures the proportion of (shortest) paths connecting pairs of non-group members that pass through the group (cf. Eq. 5); the *group-closeness*, which sums the distances from the group to all vertices outside the group (cf. Eq. 6).

Search strategies for optimal sets

When the aim is not to quantify the centrality of a specific set of nodes, but that of discovering which is/are the most central set(s) in a network, the search heuristics might come in handy. In particular, Pyntacle implements a *greedy optimization* search heuristics presented in [10] and a *brute-force* combinatorial optimization search strategy (cf. *Search algorithms* section in Methods). The former progressively replaces the components of a starting random set with all other nodes of a graph, calculating one of the formerly mentioned centrality metrics for that group, and then stops when a sub-optimal solution is obtained. The latter loops through all possible groups of a predefined size and returns only those exhibiting the best scores for any of the centrality measure. It is immediate that the computational complexity of the heuristic method is much lower than that of the exact method, at the cost of sub-optimal solutions. The brute-force search yields exact solutions, but is computationally impracticable for big networks.

Exploration of crosstalk pathways of sparse real-world networks

Real-world biological networks exhibit hierarchical organizations, where subnetworks (e.g., signaling pathways) are bridged by *crosstalk* links [15]. A number of developmental processes rely on crosstalks, where their aberrant regulation was found to be associated with inflammatory response defects as well as cancer and neurodegeneration [16, 17]. Together with the observation that causal molecular pathways often coincide with the shortest molecular paths between known disease-associated components (cf. the *network parsimony* principle [1]), these render the study of crosstalks in networks fundamental. Pyntacle eases the exploration of crosstalks by *set operations* on graphs. Individual networks can thus be compared (union, intersection and difference) or merged and then studied topologically.

These networks are typically sparse and can be analyzed employing algorithms that work best with graphs with a few edges. Pyntacle is optimized to work with increasingly large and complex networks. It provides the user with the possibility to assess the extent of sparseness of a network though mathematical indices, including the *compactness* and *completeness* indices [18, 19]. In addition, it uses these indices to choose the best implementation of computationally heavy algorithms

at run-time (e.g., the search for all the shortest paths), according to the available hardware (i.e., single or multi-core processors and GPU-enabled graphics cards) and the network topology.

Data format compatibility and reporting

Pyntacle is compliant with the Cytoscape's *SIF* data format and with the *dot* network data format. It can input and output *adjacency matrices*, *edge lists* as textual files as well as serialized binary Python objects. Graph, node, or edge attributes can be imported/exported from/to file.

Pyntacle can report any analysis result in two formats: as textual files and as rich HTML files. In particular, the *Pyntacle-Ink* module outputs an interactive, automatically generated web page that displays the graph, its attributes, and all the results of the analyses that were performed on it.

Implementation

Pyntacle is accessible via command line and exposes a Python API for fine-tuning its algorithms. It depends on iGraph for handling the graph data structure and borrowing some basic local and global topological measures and network generators.

Heavy computations of new algorithms are just-in-time compiled to native machine instructions by Numba [20] and thus run on multi-process CPU or NVIDIA-compatible GPU hardware, if available in the hosting computing infrastructure. Differently from similar packages, this allows Pyntacle to process graphs with thousands of nodes, thus helping it manage, for example, the whole human transcriptome and other networks of comparable sizes. Moreover, GPU-acceleration provides high-speed computing of the Pyntacle's algorithms, thereby making heavy and long-running tasks feasible.

The *Pyntacle-Ink* visualizer exploits HTML5, Javascript and Sigma to produce an interactive representation of the input graph, its base metrics, and a graphic rendering of the results of most of Pyntacle's algorithms (KPP, group centrality, graph generation, set operations, community detection, Figure 6B). A graph can be displayed using different layouts (i.e., *Random, Circular, ForceAtlas, Fuchterman-Reingold*), and the canvas renderer allows the visualization and smooth interaction with graphs up to 5000 nodes in size, using a web browser of a standard desktop PC. All the information about a graph and the analyses that were performed on it are stored in a JSON file; this dictionary is updated with new information whenever a new run of analysis is performed on the same graph, allowing the user to simultaneously explore the results of different algorithms and - through the use of timestamps - the results of the same algorithm run with different parameters over time. Any graphical representation can be exported as vector graphics (SVG) or PNG screenshots.

Finally, Pyntacle is fully compatible with the Jupyter Notebook.

Benchmarks

Compared with the keyplayer 1.0.3 R package [21] and Key-Player 1.44 [22], Pyntacle has the following attributes: i) available for Windows, Mac, and Linux OS; ii) available as both command line tool and API; iii) it allows management of real-world graphs in a computationally efficient way.

Wall-clock-time (WCT) comparisons of Pyntacle and keyplayer, when searching for optimal kp-sets of some real and simulated graphs, are shown in Figure 4. Noteworthy is that keyplayer is not rigorously testable here since it is a Windows-only GUI-based application.

Random networks were generated according to the Erdős–Rényi model. Six random networks, three with 100 nodes and three with 1000 nodes, were generated. These two groups of networks differed for their wiring probability, which varied as 0.3, 0.5 and 0.7. This probability is a kind of weighting function, which ranges from 0 to 1, with bigger numbers producing denser networks. Other four real networks were used: the network representing strong advice-seeking ties in global consulting company from [10] (32 vertices and 55 edges); the parasite-host food web of the Carpinteria Salt Marsh Reserve (128 vertices and 1198 edges) [11]; the *C. elegans* connectome (a modified version of the network published in [23], 279 vertices and 1960 edges) and a high-quality *C. elegans* PPI network (3303 vertices and 5561 edges, downloaded from APID [24], the Agile Protein Interactomes DataServer [25]).

WCT were measured three times for each network and centrality algorithm. DR, m-reach and DF were the only three algorithms in common between the two software packages. The sub-optimal sets of size 2 were determined by both software using their own implementations of the greedy optimization search algorithm (cf. *Search algorithms* in the Methods section). Starting from the 100-nodes random networks, Pyntacle computed all indices in fractions of seconds (or a few seconds for DF), irrespective of the wiring probability. keyplayer computed the same indices of the same networks in 4 to 9 minutes. Considering the 1000-nodes networks, keyplayer completed the computation of all indexes in more than one day, while Pyntacle took a few minutes to 5 hours (DF). Similarly, real networks were analyzed in fractions (or tenths for DF) of seconds by Pyntacle and in a few seconds to 1 hour by keyplayer, which took more than 1 day to analyze the APID network, as opposed to Pyntacle that ran for a few minutes to 17 hours. Generally, Pyntacle was 40 to 3,900 times faster than keyplayer, depending on the test.

The brute-force search algorithm yields exact solutions at the cost of an intrinsic combinatorial complexity. However, its computational load can be split into parallel processors. In Pyntacle, the best solutions are obtained after the enumeration of all possible groups of nodes and the calculation of their topological indices. Calculations are in fact independent from each other and hence suitable to be executed in parallel. When applied to our test networks with the aim to calculate the DR index, we verified that the smaller ones (≤ 100 nodes) have benefited from parallel execution only limitedly. While the *strong advice-seeking ties in global consulting company* network exhibited the best *speed-up* with the employment of 4 computing cores (1.76X), before decreasing its performance, the net execution time improvement consisted in fact only of 82 msec, on average (Fig. 5C). Similarly, 100-nodes random networks, proportionally to the rewiring probability, achieved the best speedup values with 16 cores (~8X) with an improvement of just ~4 sec (Fig. 5B). As expected, bigger networks benefited from parallel execution increasingly with the number of nodes. The Carpinteria network achieved the best speedup record (~11X) with 16 cores, although saving just 7 sec. of computation, while the Connectome peaked at ~25X with 32 cores. (Fig. 5A). The computations of 1000-nodes random networks scaled well up to 16 nodes, exhibiting comparable speedups of ~7X, ~6X,~6X, when varying the rewiring probability from 0.3 to 0.5 and 0.7, respectively. The bigger APID network exhibited the best performance with 32 cores, achieving a speedup of ~29X and terminating the computation 23 hours earlier than the non-parallel run (Fig. 5D).

Although these are far from being linear speedups, the advantage and efficacy of parallel computing strategies is evident for networks of big sizes. These results can be reproduced using a Docker image available from the Pyntacle website.

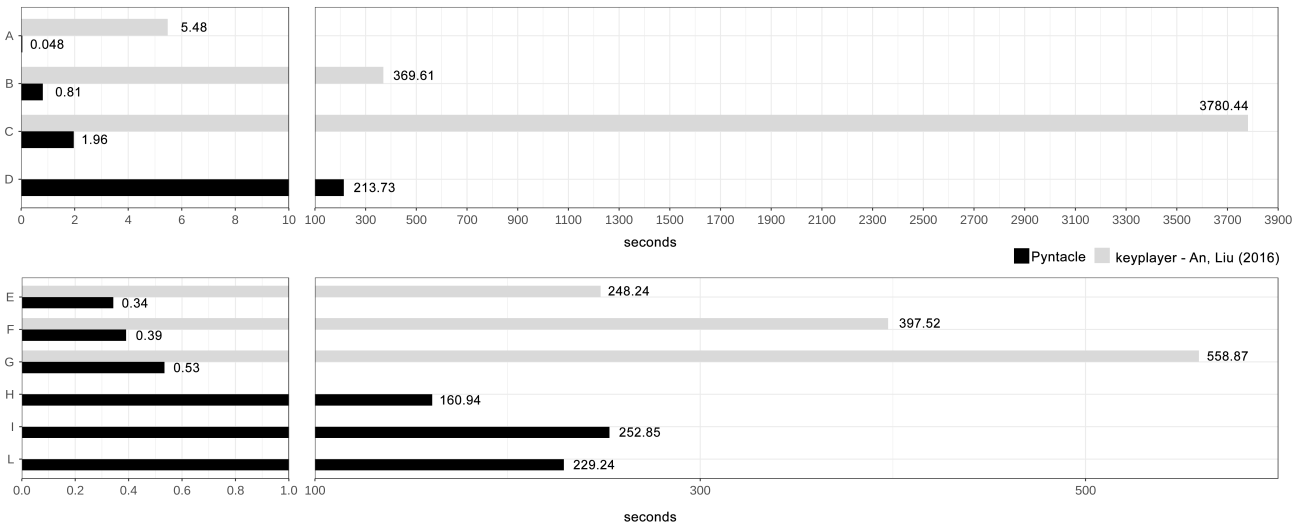

Figure 4. Greedy-optimization search, metrics: DR. (A) Strong advice-seeking ties in global consulting company (Borgatti, 2006); (B) Parasite-host food web of the Carpinteria Salt Marsh Reserve (Capocefalo, et al., 2018); (C) *C. elegans* connectome; (D) High quality *C. elegans* PPI network (APID); Erdős–Rényi random networks with (E-G) 100 nodes and rewiring probability p = 0.3, 0.5 and 0.7; (H-L) 1000 nodes and p = 0.3, 0.5 and 0.7.

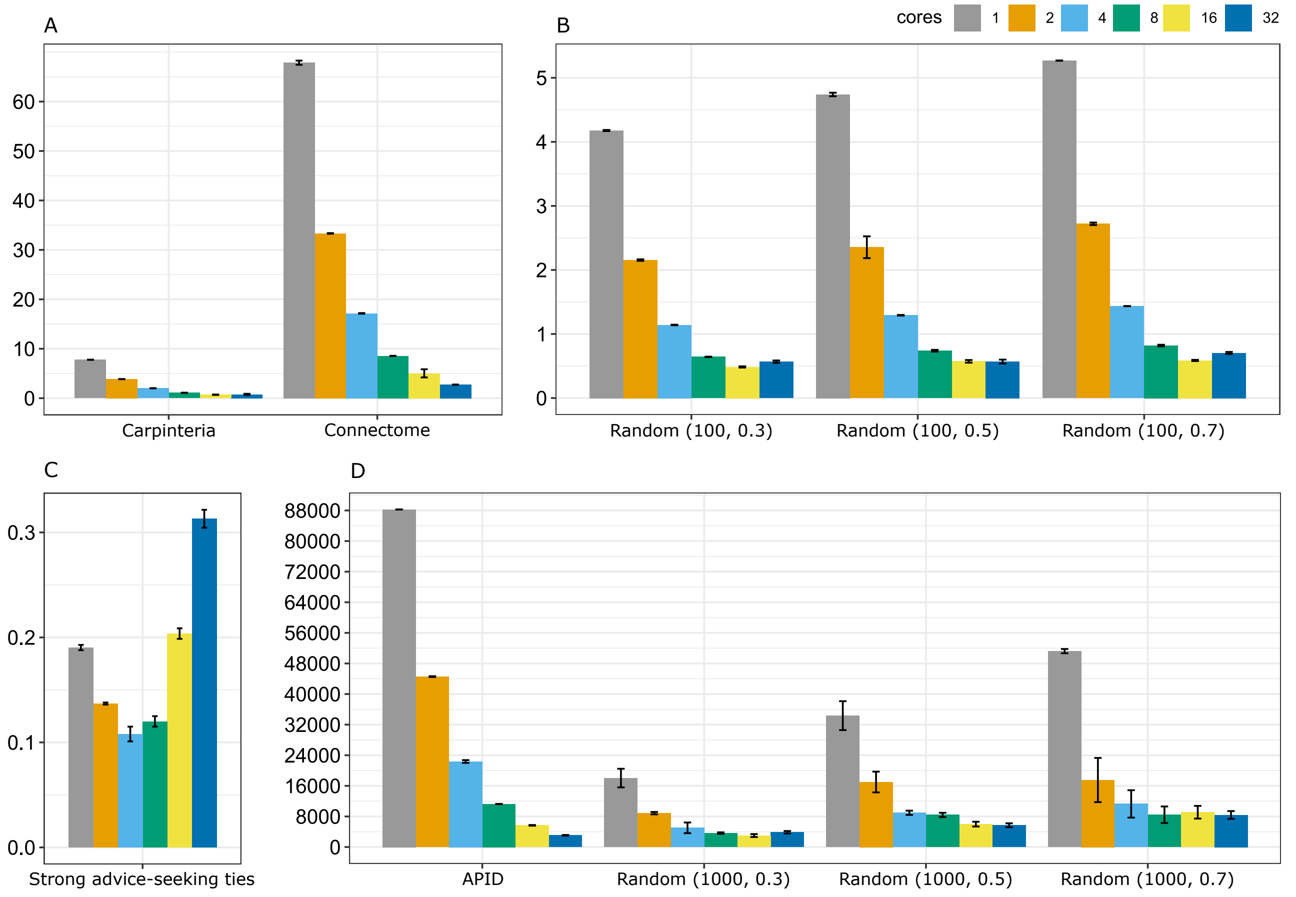

Figure 5. Improvement of execution times (in seconds) of Pyntacle using parallel computing on different networks using increasing numbers of computing cores.

Analyses

Case Study 1 – protein-protein interaction interface

NADH dehydrogenase [ubiquinone] flavoproteins 1 and 2 (NDUFV1 and NDUFV2) are two core subunits of the mitochondrial respiratory Complex 1 [26]. Their interaction is mediated by 138 interface residues (Figure 6A).

We have built a network whose edges linked interacting residues of the two proteins with the aim to identify key residues at the interface between the two proteins and whose mutations might significantly affect their interaction (Figure 6B). Thus, we computed several local topological metrics for these residues, e.g. *degree*, *betweenness*, *closeness*, *radiality* and

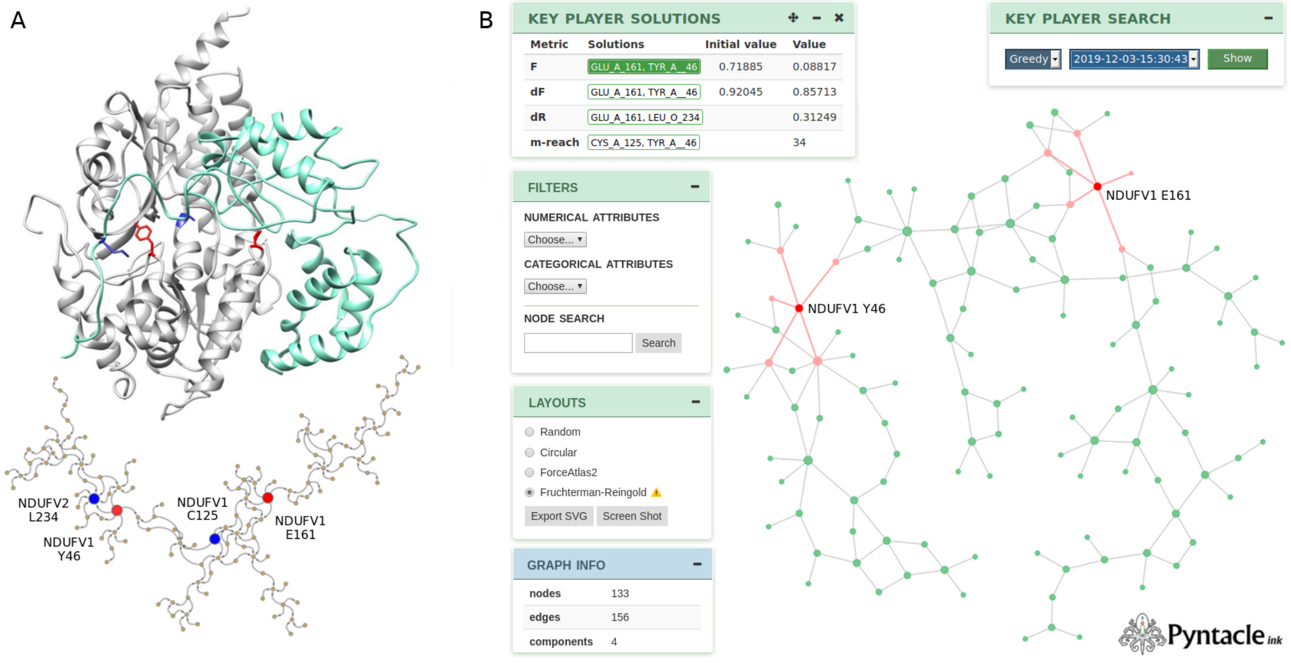

Figure 6. A: Representation of the interaction between NDUFV1 and NDUFV2, colored in white and cyan respectively (PDB id 5xtd). The 138 residues forming the interaction interface are represented as a graph (bottom) connecting residues close in space. Positive and negative key-players are colored in blue and red respectively in both the interaction structure and interaction network. B: The Pyntacle-Ink viewer. Different menus can be used to i) visualize all the analyses that have been performed on a network, ii) visualize the network general metrics, iii) filter nodes by attributes and iv) change the overall layout of the network.

a few others, but none of them showed to correlate appreciably with the contribution provided by each residue (Supplementary Figure S1) on the NDUFV1-NDUFV2 interaction energy ($\Delta\Delta G$, expressed in Kcal/mol and calculated with FoldX [27], see Methods): a maximum Pearson correlation of 0.32 was observed between $\Delta\Delta G$ and *betweenness*.

We then applied Pyntacle to the network, searching for the best positive and negative key-player sets of size 2 (colored in blue and red, respectively, in Figure 6, Supplementary data S4). The residues Glu161 and Tyr46 of NDUFV1 were identified as the best negative key-players in the network (according to both F and DF metrics), namely their removal was estimated to maximally fragment the network and thus potentially hamper the interaction between the two proteins. This was further confirmed by their energetic contributions to the interaction when mutated to Alanine ($\Delta\Delta G$ +0.9 and +3.7 Kcal/mol, respectively for Glu161 and Tyr46). Moreover, Glu161 of NDUFV1, paired with Leu234 of NDUFV2, and Cys125 of NDUFV1, paired with Tyr46, were identified as the best positive key-players pairs (respectively calculated with the DF and m-reach metrics), namely they resulted to be immediately reachable from the remaining network by direct links or indirect links joining close neighbor residues.

Contrary to Glu161 and Tyr46, Leu234 and Cys125 neither exhibited a significant $\Delta\Delta G$ when mutated to Alanine, nor were characterized by high values of local topological metrics. For these reasons, they would have been overlooked by all other techniques, even the more accurate and computationally intensive, as the Alanine-scanning [28]. Other than being computationally demanding and impracticable for large-scale analyses, Alanine-scanning is known to be blind to residues which are chemically similar to Alanine, thereby ignoring their epistatic features which are critical in some regions of the interaction interface.

All these issues are overcome with Pyntacle, which allows to look for topologically important groups of residues between proteins efficiently and regardless of their chemical structure.

Case Study 2 - miRNA-miRNA interaction network

microRNAs (miRNAs) are small RNA molecules (18-25 nucleotides) able to regulate gene expression levels through different cellular mechanisms, the most important of which is that a miRNA can recognize different messenger RNAs (mRNAs) as targets and, at the same time, one of those targets can be recognized by multiple miRNAs. Due to the renowned role played by miRNAs in tumorigenesis and cancer progression [29], we have analyzed a miRNA interaction network of patients affected by breast cancer.

Expression data of healthy and tumor tissue samples of 87 patients affected by breast cancer were retrieved from The Cancer Genome Atlas (TCGA) and were used to wire 487 miRNAs in two correlation networks, built on healthy and tumor samples, respectively. A functional association between any two miRNAs was assumed if the Pearson's correlation coefficients of their expression levels exceeded 0.5 (cf. *Case Study* 2 in Methods).

The healthy network was very dense and highly connected (average degree of 40.4), as opposed to the tumor network which was mostly disconnected (average degree of 17.4). Both networks were analyzed with Pyntacle, which identified miR-1307-3p and miR-140-3p, as negative key-players and miR-136-5p, miR-484 and miR-127-5p, as positive key-players of the healthy tissue network. These are all associated with the onset, development of breast cancer and some are even used as markers for prognosis [30, 31, 32, 33, 34, 35] (Supplementary data S5).

In the tumor network, Pyntacle identified miR-192-5p, miR-483-5p and miR-577 as negative key-players and miR-324-5p and miR-337-3p as positive key-players, all involved in *proliferation*, *cell migration* and *metastasis* of breast cancer [36, 37, 38, 39, 40] (Supplementary data S6).

It has to be noted that while these miRNAs have individually high betweenness values, it would not have been possible to infer a possible synergistic interaction between them without the key-player analysis of Pyntacle.

Discussion & concluding remarks

The motivations behind Pyntacles come from the constant growth of experimental data sets and from the increasing need to represent and analyze them with computational efficient methods. This first release of Pyntacle has been designed with these aims and thus to help researchers from different scientific fields and with different levels of computing skills to approach Network Biology and benefit from its analytical tools.

We showed the attributes of Pyntacle and its versatility in dealing with different problems, but all traceable to possibly big networks of interactive elements. In a former case study, Pyntacle was thus able to identify key-amino acids that were greatly contributing to the formation of a protein-protein interaction interface. This otherwise time-consuming task was accomplished very efficiently by translating the problem into a network analysis task, compared to current approaches that take tens of minutes to handle even small interaction interfaces. In a second case study, Pyntacle was used to analyze the TCGA data set of miRNA expression in breast cancer to build miRNA-miRNA networks. These were analyzed in search of microRNAs that were occupying key positions in the network, which were later discovered to be already known biomarkers or responsible for the onset and progression of breast cancer.

Concluding, Pyntacle represents a starting point for large-scale Network Biology studies. Being a modular framework, it will be expanded to handle *weighted* networks, in the near future, and *directed* networks, immediately after. It will also be enriched with new optimization search algorithms and with a new algorithm to compute the set *nestedness*. New applications and use-cases are envisaged, the currently most concrete is the one concerning the analysis of trajectories of Molecular Dynamics simulation of proteins.

Potential implications

The main problem concerning most of the currently available network analysis tools, which is also the main reason why we made Pyntacle, is that these do not handle networks of medium to big sizes efficiently. This issue not only regards the big networks and the suitability and effectiveness of the currently available algorithms to analyze them, but also their practicability over a reasonable time axis and in terms of required computing resources. This point is critical for most fields of research, from Biology to Medical and Social sciences, where systems are naturally big and complex (e.g., the whole *Proteome*, the *Diseasome* and the *Socialnomics* to mention a few). In particular, a field that at the time of this writing is hitting the headlines, i.e. Epidemiology, is greatly developing in terms of the capability to draw contagion maps and predict infection growth over time. These maps are actually networks where nodes are people and edges are relationships occurred in recent and short periods of time. The way these networks could be studied are many. One could be that of determining the front of the infection, namely a group made by healthy people that are *close* to the affected people and *highly social* and to administer a vaccine, in order to curb the infection. Another possibility would be that of determining the minimum possible number of communication routes to be closed at a national level in order to implement a clever lockdown. These and several other options may be implemented in Pyntacle, using ad-hoc algorithms and computing protocols tailored for big networks.

Methods

Key-Player and group-centrality metrics

Pyntacle tackles the problem of identifying key-player nodes that, together, optimally diffuse *something* through a network or maximally disrupt or fragment a network when removed. The classes of algorithms are thus two: one that measures the importance of a set on the basis of its impact on the remaining nodes of a network, and another that does it by considering the sole properties of the elements of a set. The former class, also known as KPP-Neg, measures the fragmentation of a network because of the removal of a set of nodes. It is composed by the DF metrics:

$$DF = 1 - \frac{2\sum_{i>j}\frac{1}{d_{ij}}}{n(n-1)} \quad (1)$$

where d_{ij} denotes the distance between the i_{th} and the j_{th} node. It ranges from 1, when all nodes are adjacent to 0, when all nodes are *isolates*.

The latter class, also known as KPP-Pos, measures the overall cohesion that members of a set have with the remainder of the network and is made by:

$$DR = \frac{\sum_j \frac{1}{d_{Kj}}}{n} \quad (2)$$

where n is the size of the graph and d_{Kj} denotes the minimum distance (shortest path) between any member i of the set K and the remaining nodes j in the graph. Similarly, the *m-reach* metrics counts how many unique nodes can be reached from K in m steps or less. The formulation is:

$$C_K = \sum_{j \in V \setminus K} \cup \frac{1}{d_{ij}} \quad (3)$$

where C_K ranges from 0 to $n - k$, n representing the size of the graph and k that of the considered set of nodes. It is important to notice that this index assumes that all paths of length m or less are equally important and that all paths longer than m are wholly irrelevant [10].

Pyntacle further extends the standard network centrality measures of *degree*, *closeness*, and *betweenness* to groups of nodes, in a way that if, e.g., *group-degree* and *degree* centrality measures are applied to groups consisting of single elements, they yield identical results. This class of metrics is made by the *group-degree* centrality measure, which is intended as the number of non-group nodes that are connected to group members. Multiple ties to the same node are counted only once. It is defined as:

$$GD_K = \frac{\sum_{i \in K, j \in V \setminus K} a_{ij}}{\|V \setminus K\|} \quad (4)$$

where a_{ij} equals 1 when i and j are adjacent nodes, if $i \in K$ and $j \in \{V \setminus K\}$, and counting $a_{i,j}$ and $a_{v,j}$ only once $\forall v \in K$ when $a_{i,j} = 1$ and $a_{v,j} = 1$. Hence, the group-degree centrality ranges from 0 to 1, if the group K is completely isolated or fully connected to all other nodes. *Group-betweenness* centrality of a set K is defined as the number of shortest paths connecting any two nodes u and v passing through K over the number of all paths between the two.

$$GB_K = \frac{p_{u,v}(K)}{p_{u,v}} \quad (5)$$

where u and v are any pair of nodes not belonging to the group K, $p_{u,v}(K)$ represents the number of shortest paths connecting u and v and that traverses K, while $p_{u,v}$ is the total number of shortest paths between u and v. *Group-closeness* of a group K is defined as the sum of the *minimum*, *maximum*, *average* distances from the nodes belonging to the group to all other nodes outside the group.

$$GC_K = \frac{\sum_{j \in V \setminus K} \bar{d}_{Kj}}{\|V \setminus K\|} \tag{6}$$

where $\bar{d}_{ij}$ is the minimum, maximum or average distance between nodes in K and all other nodes.

Search algorithms

When the aim is not to quantify the centrality of a specific set of nodes, but to discover which is/are the most central set/s of nodes in a network, search heuristics might come in handy. In particular, Pyntacle implements a greedy optimization search heuristics presented in [10], and a brute-force combinatorial optimization search strategy. The former follows this naïve algorithm:

Algorithm 1 Greedy optimization search

```
procedure greedySearch(k: int, V: list, F: lambda)
    score = 0.0
    scoredict = {} ← hashmap
    halt = false
    K = drawNodes(k, V)
    while not halt do
        foreach u ∈ K do
            foreach v ∈ V \ K do
                Ktemp = swap(K, u, v)
                scoretemp = F(Ktemp)
                scoredict[(u, v)] = scoretemp
        u_best, v_best = getPairsWithBestScore(scoredict)
        bestscore = scoredict[(u_best, v_best)]
        if best_score > score then
            K = swap(K, u_best, v_best)
            score = bestscore
        else
            halt = true
    return (K, score)
    end
```

F is an appropriate key-player metrics; `drawnodes` is a function that randomly picks k nodes from V, which contains all nodes of the network; `swap(K,u,v)` substitutes the element u in K with v; `getPairsWithBestScore` is a function that returns the pairs which yielded the best centrality score. This method progressively replaces the components of a starting random set K with all other nodes of a graph, calculating one of the formerly mentioned centrality metrics for that group and then stops when a sub-optimal solution is obtained.

The brute-force combinatorial optimization search strategy implemented in Pyntacle loops through all possible groups of a predefined size (k) and returns only those exhibiting the best scores for any of the previous centrality measure (F). Even if the computation can be performed in parallel (the **foreach** loop below), it is immediate that the computational complexity of the heuristic method is much lower than that of this method, at the cost of yielding sub-optimal solutions. The algorithm below returns exact solutions, but is computationally impracticable with big networks.

Algorithm 2 Brute-force search

```
procedure bruteforceSearch(k: int, V: list, F: lambda)
    bestscore = 0.0
    scoredict = {} ← hashmap
    allsets = generateCombinations(k, V)
    foreach set ∈ allsets do
        scoretemp = F(set)
        scoredict[set] = scoretemp
    bestsets = getSetsWithBestScore(scoredict)
    bestscore = scoredict[bestsets]
    return (bestsets, bestscore)
    end
```

Set operations on graphs

Graph *union*, $G1 \cup G2$, is implemented as $(V1 \cup V2, E1 \cup E2)$, namely as the union of nodes (V) and edges (E). Graph *intersection* is defined as $G1 \cap G2 = (V1 \cap V2, E1 \cap E2)$, where only common nodes and edges are reported in the resulting graph. The *difference* between $G1$ and $G2$ results in a graph with nodes and edges only present in $G1$ and not in $G2$. Since the difference between graphs is not reciprocal, $G1 - G2 \neq G2 - G1$.

Case study 1

Chains A and O of the PDB structure `5xtd` were considered for the analysis of NDUFV1 and NDUFV2, respectively. First, the structure has been repaired (*RepairPDB* module of FoldX) and thus allowing more relaxed residue side-chain rotamers and solving clashes. Then, residues located at the interaction interface and the interaction energy were determined with the *AnalyseComplex* module of FoldX. Alanine-scanning of the interface residues was performed by substituting each amino acid with an Alanine residue (*BuildModel* module). The interaction energy of each mutant has been calculated with the *AnalyseComplex* module in order to determine the $\Delta\Delta G$ of the mutant compared to the wild-type interaction structure. A residue-residue interaction network has been built connecting residues if any of their non-hydrogen atoms (both backbone and side-chain) were in a 4.5Å radius from each other. The network has been then analyzed with the *keyplayer* module of Pyntacle and a greedy-search algorithm was used to find the optimal key-player sets of size 2, considering all available metrics (F and DF, as KPP-Neg. and DR and m-reach, with m set to 2, as KPP-Pos).

Case study 2

Expression levels of 547 miRNAs in 87 healthy and 87 tumor breast samples were retrieved from TCGA. Separately for healthy and tumor individuals, correlations of expression between any possible pairs of miRNAs (149, 331 total pairs) were calculated by Pearson correlation coefficient. Only significant values that exceeded ± 0.5 were considered to represent edges connecting miRNAs in the *healthy* and *tumor* networks. In both networks, the best KPP-Pos and KPP-Neg sets of size 2 were sought using the Pyntacle's greedy optimization search and calculating F and DF, as KPP-Neg, and DR and m-reach, with m set to 2, as KPP-Pos.

Availability of source code and requirements

- Project name: **Pyntacle**
- Project home page: http://pyntacle.css-mendel.it
- Operating systems: **Linux, Mac and Windows**
- Programming language: **Python 3.5+**
- Other requirements: **CUDA toolkit** (optional)
- License: e.g. **GNU GPL 3.0**

Source code is stored in GitHub. Installation procedures, tutorials, case studies, a Docker container for benchmark reproducibility and APIs are all available from the Pyntacle's website.

Declarations

List of abbreviations

- **KPP-Neg**: Key-Player Problem/Negative
- **KPP-Pos**: Key-Player Problem/Positive
- **APID**: Agile Protein Interactomes
- **TCGA**: The Cancer Genome Atlas
- **RNA**: Ribonucleic Acid
- **mRNA**: messenger RNA
- **miRNA**: microRNA
- **NADH**: Nicotinamide Adenine Dinucleotide - Hydrogen (reduced)
- **API**: Application Programming Interface
- **WCT**: Wall-clock-time

Competing Interests

The authors declare that they have no competing interests.

Funding

This study was supported by the Italian Ministry of Health (Ricerca Corrente 2020 RC20DS-COMU). Conflict of Interest: none declared.

Author's Contributions

LP designed and performed the experiments; MT implemented Pyntacle-Ink and took care of the package maintenance and deployment; DC implemented the command line interface, the set operations, wrapped the iGraph modules and performed the benchmarks; TB, SC and FP contributed to the case study analysis; MC contributed to the definition of the case studies; FJ oversaw the implementation of the topology metrics; TM designed and implemented the software and oversaw the project.

Acknowledgements

We are grateful to Christoph Gohlke, from the Laboratory for Fluorescence Dynamics, University of California, for providing Windows unofficial binaries of the iGraph Python extension package; Juliana Pereira for testing and constructing discussions and to NVIDIA Corporation for supporting this research. The results of case study 2 shown here are in whole or part based upon data generated by the TCGA Research Network.

References

1. Barabasi AL, Gulbahce N, Loscalzo J. Network medicine: a network-based approach to human disease. Nat Rev Genet 2011;12:56–68.
2. Goh KI, Cusick ME, Valle D, Childs B, Vidal AL M Barabási. The human disease network. Proc Natl Acad Sci USA 2007;104:8685–8690.
3. Jeong H, Mason SP, Barabási AL, Oltvai ZN. Lethality and centrality in protein networks. Nature 2001;411:41–42.
4. Luo H, Lin Y, Gao F, Zhang CT, Zhang R. DEG 10, an update of the Database of Essential Genes that includes both protein-coding genes and non-coding genomic elements. Nucleic Acids Research 2014;42:D574–D580.
5. Wachi YK S, Wu R. Interactome-transcriptome analysis reveals the high centrality of genes differentially expressed in lung cancer tissues. Bioinformatics 2005;21:4205–4208.
6. Jonsson PF, Bates PA. Global topological features of cancer proteins in the human interactome. Bioinformatics 2006;22:2291–2297.
7. Xu J, Li Y. Discovering disease-genes by topological features in human protein–protein interaction network. Bioinformatics 2006;22:2800–2805.
8. Freeman LC. A Set of Measures of Centrality Based on Betweenness. Sociometry 1977;40:35–41.
9. Everett MG, Borgatti SP. The centrality of groups and classes. The Journal of Mathematical Sociology 1999;23(3):181–201. https://doi.org/10.1080/0022250X.1999.9990219.
10. Borgatti SP. Identifying sets of key players in a social network. Computational & Mathematical Organization Theory 2006;12:21–34.
11. Capocefalo D, Pereira J, Mazza T, Jordán F. Food Web Topology and Nested Keystone Species Complexes. Complexity 2018;2018.
12. Almeida-Neto M, Guimarães P, Guimarães Jr PR, Loyola RD, Ulrich W. A consistent metric for nestedness analysis in ecological systems: reconciling concept and measurement. Oikos 2008;117.
13. Borgatti SP, Everett MG. A Graph-theoretic perspective on centrality. Social Networks 2006;28:466–484.
14. Freeman LC. Centrality in networks: I. Conceptual clarification. Social Networks 1979;1:215–239.
15. Vert G, Chory J. Crosstalk in Cellular Signaling: Background Noise or the Real Thing? Dev Cell 2011;21(6):985–991.
16. Zolezzi JM, Inestrosa NC. Wnt/TLR Dialog in Neuroinflammation, Relevance in Alzheimer's Disease. Front Immunol 2017;24(8):187.
17. Qu X, Tang Y, Hua S. Immunological Approaches Towards Cancer and Inflammation: A Cross Talk. Front Immunol 2018;20(9):563.
18. Menniti S, Castagna E, Mazza T. Estimating the global density of graphs by a sparseness index. Applied Mathematics and Computation 2013;224:346–357.
19. Mazza T, Romanel A, Jordán F. Estimating the divisibility of complex biological networks by sparseness indices. Briefings in Bioinformatics 2010;11(3):364–374.
20. Crist J. Dask & Numba: Simple libraries for optimizing scientific python code. In: 2016 IEEE International Conference on Big Data (Big Data); 2016. p. 2342–2343.
21. An WH, Liu YH. An R Package for Locating Key Players in Social Networks. The R Journal 2016;8(1):257–268.
22. Borgatti SP, KeyPlayer 1.44; 2019. [Online; accessed 06-August-2019]. http://www.analytictech.com/keyplayer/keyplayer.htm.
23. Towlson EK, Vértes PE, Ahnert SE, Schafer WR, Bullmore ET. The Rich Club of the C. elegans Neuronal Connectome.

Journal of Neuroscience 2013;33(15):6380–6387. https://www.jneurosci.org/content/33/15/6380.
24. Alonso-López D, Campos-Laborie FJ, Gutiérrez MA, Lambourne L, Calderwood MA, Vidal M, et al., Agile Protein Interactomes DataServer; 2019. [Online; accessed 07-August-2019]. http://apid.dep.usal.es/.
25. Alonso-López D, Campos-Laborie FJ, Gutiérrez MA, Lambourne L, Calderwood MA, Vidal M, et al. APID database: redefining protein-protein interaction experimental evidences and binary interactomes. Database 2019 01;2019. https://doi.org/10.1093/database/baz005.
26. Guo R, Zong S, Wu M, Gu J, Yang M. Architecture of Human Mitochondrial Respiratory Megacomplex I2III2IV2. Cell 2017;170:1247–1257.
27. Schymkowitz J, Borg J, Stricher F, Nys R, Rousseau F, Serrano L. The FoldX web server: an online force field. Nucleic Acids Res 2005;33:W382–W388.
28. Weiss GA, Watanabe CK, Zhong A, Goddard A, Sidhu SS. Rapid mapping of protein functional epitopes by combinatorial alanine scanning. Proc Natl Acad Sci U S A 2000;97:8950–8954.
29. Reddy KB. MicroRNA (miRNA) in cancer. Cancer Cell International 2015;15(38).
30. McGuire A, Brown JAL, Kerin J. Metastatic breast cancer: the potential of miRNA for diagnosis and treatment monitoring. Cancer Metastasis Rev 2015;34:145–155.
31. Wang DY, Gendoo DMA, Ben-David Y, Woodgett JR, Zacksenhaus E. A subgroup of microRNAs defines PTEN-deficient, triple-negative breast cancer patients with poorest prognosis and alterations in RB1, MYC, and Wnt signaling. Breast Cancer Res 2019;21(18).
32. Pronina IV, Loginov VI, Burdennyy AM, Fridman MV, Senchenko VN, Kazubskaya TP, et al. DNA methylation contributes to deregulation of 12 cancer-associated microRNAs and breast cancer progression. Gene 2017;604:1–8.
33. Li Q, Yao Y, Eades G, Liu Z, Zhang Y, Zhou Q. Downregulation of miR-140 promotes cancer stem cell formation in basal-like early stage breast cancer. Oncogene 2014;33:2589–2600.
34. Vos S, Vesuna F, Raman V, van Diest PJ, van der Groep P. miRNA expression patterns in normal breast tissue and invasive breast cancers of BRCA1 and BRCA2 germ-line mutation carriers. Oncotarget 2015;6:32115–32137.
35. Wang X, Zhu J. Mir-1307 regulates cisplatin resistance by targeting Mdm4 in breast cancer expressing wild type P53. Thorac Cancer 2018;9:676–683.
36. Wang Z, Wang J, Yang Y, Hao B, Wang R, Li Y, et al. Loss of has-miR-337-3p expression is associated with lymph node metastasis of human gastric cancer. Journal of Experimental & Clinical Cancer Research 2013;32(76).
37. Zuo XL, Chen ZQ, Wang JF, Wang JG, Liang LH, Cai J. miR-337-3p suppresses the proliferation and invasion of hepatocellular carcinoma cells through targeting JAK2. Am J Cancer Res 2018;8:662–674.
38. Yin C, Mou Q, Pan X, Zhang G, Li H, Sun Y. MiR-577 suppresses epithelial-mesenchymal transition and metastasis of breast cancer by targeting Rab25. Thoracic Cancer 2018;9:472–479.
39. Cioce M, Valerio M, Casadei L, Pulito C, Sacconi A, Mori F, et al. Metformin-induced metabolic reprogramming of chemoresistant ALDHbright breast cancer cells. Oncotarget 2014;5:4129–4143.
40. Li JY, Jia S, Zhang WH, Zhang Y, Kang Y, Li PS. Differential Distribution of microRNAs in Breast Cancer Grouped by Clinicopathological Subtypes. Asian Pacific Journal of Cancer Prevention 2013;14:3197–3203.

Supplementary Material (6 xls sheets)

Click here to access/download
Supplementary Material
Supplementary Data S1-S6.xls

Supplementary Figure S1

Click here to access/download
Supplementary Material
Supplementary Figure S1.pdf

Rome, March 24th 2020

To the **GigaScience** editorial office,

My colleagues and I are pleased to attach our original paper entitled **"Pyntacle: a parallel computing-enabled framework for large-scale network biology analysis"** for consideration for publication in **GigaScience** as a Technical Note.

Exploiting Graph Theory to study properties of biological networks and to identify biomarkers or molecules of compelling importance is today a key-step and of broad interdisciplinary interest in Computational and Systems Biology. However most natural systems are **big** in size, **complex** and often characterized by **convoluted** mechanisms of interaction, such as *epistasis*, *pleiotropy*, and *trophism.* These factors make traditional network analysis methods often ineffective, both because their algorithmic complexity makes them computationally intractable with big/real networks and since they were designed to look for the reason of a particular cellular mechanism into individual components of networks.

Pathogenic phenotypes, most natural events as well as social behaviors are not caused, in fact, by individual molecules/species/actors, rather these originate from their orchestration. In particular, a field that at the time of this writing is hitting the headlines is the Epidemiology. At the boundaries of Statistics, Medicine, and Social sciences, this discipline is not only greatly developing in terms of the capability to draw huge contagion networks and predict infection growth over time, but in the way of managing the infections. Identifying quickly the front of the infection, namely the smallest group of people to be monitored and quarantined, is the only way to curb infection.

For this and other fields of research, we designed and developed **Pyntacle**, a computing framework that eases the search for important groups of "network components" (e.g., molecules or biomarkers) in real-life networks. The concept of *importance* is here declined in terms of *reachability*, *fragmentation* and topological *centrality*, which are keywords in network biology, social sciences, and several other affine scientific disciplines.

Pyntacle reshapes complex analysis methods for fast execution on multi-core and GPU computing infrastructures. It leverages on machine learning algorithms to quickly search and find optimal network sets of components. It renders analytical results off-the-shelf through *Pyntacle-Ink*, an automatically generated viewer that exploits the features of HTML5, Javascript and Sigma.js to draw an interactive and user-friendly representation of a network, its metrics and the results of most of Pyntacle's algorithms.

Pyntacle is freely available from http://pyntacle.css-mendel.it. It runs on Linux, Mac and Windows OS. Case studies, tutorials, APIs, benchmarks are available throughout the website to ease the approach of the user to the software and topic.

We confirm that this article is an original unpublished contribution and is not under consideration for publication elsewhere. All authors have participated in the work and take full public responsibility for the content of the paper.

Yours sincerely

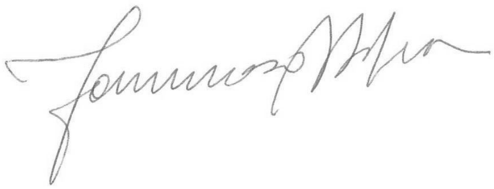

Tommaso Mazza, PhD
Bioinformatics Laboratory, head
Casa Sollievo della Sofferenza - Mendel
Viale Regina Margherita 261 - 00198 Roma IT

Tel: +39 06 44160526 - Fax: +39 06 44160548
E-mail: t.mazza@css-mendel.it
Web page: http://www.css-mendel.it/
Web page: http://bioinformatics.css-mendel.it
