# Supplementary material for: Pyntacle: a parallel computing-enabled framework for large-scale network biology analysis
Source: Gigascience. 2020 Oct 21;9(10):giaa115. doi: 10.1093/gigascience/giaa115 (PMC7576925; doi:10.1093/gigascience/giaa115)
Supplement: giaa115_GIGA-D-20-00087_Revision_1 [file giaa115_giga-d-20-00087_revision_1.pdf]

# GigaScience

## Pyntacle: a parallel computing-enabled framework for large-scale network biology analysis

--Manuscript Draft--

| | |
|---|---|
| **Manuscript Number:** | GIGA-D-20-00087R1 |
| **Full Title:** | Pyntacle: a parallel computing-enabled framework for large-scale network biology analysis |
| **Article Type:** | Technical Note |
| **Funding Information:** | |
| **Abstract:** | Some natural systems are big in size, complex and often characterized by convoluted mechanisms of interaction, such as epistasis , pleiotropy , and trophism , which cannot be immediately ascribed to individual natural events or biological entities, but that are often derived from group-effects. However, the determination of important groups of entities, like genes or proteins, in complex systems is considered a computationally hard task. Here, we present Pyntacle, a high-performance framework designed to exploit parallel computing and Graph Theory to efficiently identify critical groups in big networks and in scenarios that cannot be tackled with traditional network analysis approaches. We showcase potential applications of Pyntacle with transcriptomics and structural biology data, thereby highlighting the outstanding improvement in terms of computational resources over existing tools. |
| **Corresponding Author:** | Tommaso Mazza, Ph.D.<br>IRCCS Casa Sollievo della Sofferenza<br>Roma, Please Select ITALY |
| **Corresponding Author Secondary Information:** | |
| **Corresponding Author's Institution:** | IRCCS Casa Sollievo della Sofferenza |
| **Corresponding Author's Secondary Institution:** | |
| **First Author:** | Luca Parca, Ph.D. |
| **First Author Secondary Information:** | |
| **Order of Authors:** | Luca Parca, Ph.D. |
| | Mauro Truglio |
| | Tommaso Biagini |
| | Stefano Castellana |
| | Francesco Petrizzelli |
| | Daniele Capocefalo |
| | Ferenc Jordán |
| | Massimo Carella |
| | Tommaso Mazza, Ph.D. |
| **Order of Authors Secondary Information:** | |
| **Response to Reviewers:** | << please note that following the cover letter, in the same PDF file, the editor and reviewers can find a better formatted rebuttal letter, with images attached, which can better support our answers>><br><br>Reviewer #1: "Pyntacle: a parallel computing-enabled framework for large-scale network biology analysis" GigaScience manuscript (Technical Note) GIGA-D-20-00087<br><br>This manuscript describes a software library for graph computations usable as both a |

Powered by Editorial Manager® and ProduXion Manager® from Aries Systems Corporation

Python library and command line interface. In addition, it describes an interactive viewer output (Pyntacle-Ink) module for visualizing the results. Although Pyntacle is a general-purpose graph library, it has a particular focus (as does the manuscript) on group centrality metrics and the "key player problem" (KPP), which is the computationally intractable problem of finding sets of (rather than individual) nodes in a network which maximize (or minimize) a group centrality metric.

This distinguishes it from other general-purpose network packages, which usually do not include algorithms for the KPP, while those packages that do address it tend to be specific to that problem, and not general purpose libraries (see section C below).

Another distinguishing feature of Pyntacle is that it is targeted particularly at large-scale network biology analysis, and emphasizes parallel computing. Although the KPP is well-known in social network analysis (from which it originated), it is not so well-known in biology (although not unknown, see A.1.1 below) and so this manuscript makes a contribution in introducing it (and making available usable multi-platform software in a platform widely used in bioinformatics, i.e. Python) to the field.

A. Notes on the manuscript
--------------------------
The manuscript is generally clear, giving a (quite lengthy) introduction and exposition of the KPP, description of the software and algorithms implemented, benchmark results, and two case studies.
The software (both source code and installation packages), documentation, and data sets required for reproducibility are all referenced and freely available from the project website (although see A.1.3 below for an exception).

A.1. Major issues
These are points I think must be addressed before the manuscript can be accepted.

A.1.1. Insufficient references to existing research literature.
The KPP originated in social network research with the "key player problem" so named by Borgatti (2006). However in other fields, and specifically computer science and applied mathematics (operations research for example), the KPP and variants are known as "critical element detection" or the "critical node problem" (CNP) (Walteros & Pardalos, 2012), or the "critical node detection problem" (CNDP), of which the CNP is a variant (Lalou et al. (2018)).

This is an active research field, as evidenced by the review paper Lalou et al. (2018), and although a review of this field is clearly outside the scope of this manuscript (technical note), at least a brief mention of the relationship of the KPP to the CNDP, and a citation of (for example) the Lalou et al. (2018) review paper would be of value to readers and not leave them with the misleading impression that the KPP has only been studied in a couple of papers and only the brute-force and greedy heuristic algorithms have been studied to solve it.

As well as this general issue, I think some references to the prior use of the KPP (or related CNDP variants) in biology would be welcome, for example in protein-protein interaction (PPI) networks (Boginsnki & Commander, 2009; Tomaino et al., 2012), and genetic regulatory networks (Celestini et al., 2019). For a more general perspective on network methods in drug discovery, the extensive review of Csermely et al. (2013) could be informative.

In the concluding remarks (p. 8) where extension to weighted and directed networks is mentioned, it might be noted that some work exists on the CNDP in directed graphs (Paudel et al., 2018) and KPP on weighted graphs (McGuire et al., 2015; Yang, 2015; Jiang & Liu, 2019). Indeed the R keyplayer package is claimed to work with both directed and weighted networks (An & Liu, 2016).

Although reproducing results from the many algorithms for solving the KPP or CNDP variants (either exactly i.e. to optimality, or to potentially suboptimal solutions with heuristics) is clearly impractical, rough comparisons to other methods could be facilitated by running the benchmarks on the Critical Node Detection Problem

Powered by Editorial Manager® and ProduXion Manager® from Aries Systems Corporation

Benchmark Instances (Ventresca 2012), available from http://individual.utoronto.ca/mventresca/cnd.html, which are used in many CNDP papers.

> We thank the reviewer for the suggested reads, we integrated them in the revised manuscript. As far as the proposed benchmark is concerned, we think that it would be very informative and useful. However, we prefer to postpone this to the next future for two reasons:
1- as the reviewer has noticed, Pyntacle is equipped with just one heuristic method. We think that the suggested effort would be much more effective to take when Pyntacle will run several more algorithms. This will happen with the next major release, scheduled to come out sometimes in the second half of the next year.
2- we are aware that those presented are preliminary benchmarks and we were struggling with whether to present them or not. We would prefer to keep them at this preliminary level or, eventually, completely removing them, if suggested by the reviewers.

A.1.2. Insufficient references to existing software.
Although a review of existing network libraries is beyond the scope of the manuscript, it could be useful for readers to at least mention some other general purpose graph libraries usable from Python such as graph-tool (Peixoto, 2014), igraph (Csardi & Nepusz, 2006), NetworkX (Hagberg et al., 2008), and SNAP (Leskovec & Sosič, 2016). (See Section C below for more).

Most particularly, it is noted that Pyntacle uses igraph modules, yet the igraph paper (Csardi & Nepusz, 2006) is not cited.

In addition, although the Analytic Technologies KeyPlayer program and the R keyplayer package (An & Liu, 2016) are mentioned, the influenceR R package (Jacobs et al., 2015) is not mentioned. Although this package only solves KPP-Pos with the DF metric, as I show in Section B below (on a single example) it does so much faster than Pyntacle.

> We integrated the references in the revised manuscript.

A.1.3. Reproducibility (data availability) of case studies.

For Case Study 1, I was easily able to reproduce the 3D protein cartoon in Fig. 6 by downloading the 5XTD structure from the RCSB PDB into PyMOL (Schrödinger, LLC), selecting the appropriate two chains, and locating the key player residues as shown. However to obtain the network of "The 138 residues forming the interaction interface" I would (according to the Methods) have to conduct several operations in FoldX and then extract the network according to a distance threshold described in the Methods. Although it seems there is sufficient detail to reproduce this, it requires a registration to use FoldX and some further work construct the network.

I think it would assist reproducibility (an important consideration in the GigaScience journal) to supply the network as constructed by the authors as a Supplementary Information file (as for example the results of the Pyntacle analysis are already supplied in an Excel file).

Similarly, the network constructed for Case Study 2 could also be made available as Supplementary Information.

> We have stored the adjacency matrices of the two case studies in GigaDB. N.b., A member of the GigaScience editorial team confirmed that a link to these files will be made available eventually after the publication of this piece of research. Moreover, we have explained how we made them in order to improve replicability.
In fact, in Case Study 1, the 138 residues identified via Foldx are those considered at the interface and for which energetic contributions were calculated. The network we built has been obtained by directly analyzing the PDB file of the complex, connecting residues of different chains, and relying only on distance constraints (therefore avoiding external software like FoldX). As we demonstrate in the case study, valuable information is gained nonetheless, without using external software (FoldX) and in much

Powered by Editorial Manager® and ProduXion Manager® from Aries Systems Corporation

less time.

A.1.4. Use of "brute-force" algorithm and "large-scale".

The manuscript is described (including in the title) as being for "large-scale" network analysis, and parallelism is emphasized. However (related to point A.1.1 above) it seems to me that the emphasis is perhaps misplaced. To be specific, two algorithms are implemented and benchmarked: the "brute-force" algorithm, and the greedy heuristic algorithm of Borgatti (2006). Parallelism then mainly seems to be discussed in relation to the brute-force algorithm.

However, as the KPP is known to be computationally intractable and indeed NP-complete (Arulselvan et al., 2009), a brute-force algorithm is never going to be practical, and certainly not for large-scale instances. For this reason there are two practical approaches: (a) exact solution by more sophisticated methods (for example integer linear programming (ILP), semidefinite programming (Jiang et al., 2017), branch-and-cut methods, etc.), and (b) heuristic algorithms to find a potentially suboptimal solution, but much faster. Note that the latter are also vital as input into the former (as bounds on the solution). For a review of these approaches see Lalou et al. (2018).

Hence my concern is that implementing and applying parallel computation to the brute-force approach is misplaced, as it can never be competitive with sophisticated exact solution algorithms (particularly where these could also be parallelized) on large-scale problems.

Regarding the heuristic (greedy) algorithm, it also seems that better results could be obtained with a more sophisticated heuristic, as already foreshadowed by Borgatti (2006). For example I demonstrate in Section B below, that the influenceR program is two orders of magnitude faster (albeit on a single example) than Pyntacle for KPP-Pos, as it uses a different heuristic algorithm. In addition there are many publications showing superior results with different heuristic algorithms for CNDP variants (see Lalou et al., 2018 for a review).

> BF was implemented because it is exact and without aiming to compete with any other search algorithm. The fact that we implemented some parallelism for it is because some fields of research, like ecology, deal with small/medium size networks, where size is in the order of a few tens/hundreds of nodes. Why should we yield suboptimal results for these when exact is achievable?
We were not clear enough on this: parallelism was applied to BF (coarse+fine grain) as well as on heuristics (fine grain only). Fine-grain parallelism applies directly to the way we enumerate shortest-paths and, by this feature, Pyntacle drops the computational burden of elaborating big matrices/graphs. This happens regardless of whether one uses BF or GO search strategies. However, we agree that Pyntacle could greatly benefit from more sophisticated algorithms and the implementation of a range of different search and optimization algorithms (e.g. particle swarm, ant colony, stochastic gradient descent, etc.), that the user can select from, is in our plans.

A.1.5. Details of parallelism and benchmarks.

From my understanding of the manuscript (and some experiments I tried with it detailed in Section B below), there are two types of parallelism used in Pyntacle. First, the "fine grained" parallelism it obtains simply from using the Numba library, and second, explicit "coarse grained" parallelism. The first type uses multithreading in lower level numeric libraries, similar to Microsoft R/Open using multithreaded linear algebra routines. The second type is used only for the brute-force algorithm however (and on Linux seems to work by forking new processes rather than using threads).

> This reviewer is almost correct. Multi-threading is exploited by Numba in the calculation of all shortest-paths (Floyd-Warshall) and only for this task. We do not use any sort of off-the-shelf linear algebra library. Numba allows us to release the global interpreter lock when computing a (not so) restricted set of manipulation tasks on Numpy arrays. With the GIL released, we can spawn threads, handle concurrency, and, therefore, save a for-loop from the computation of Floyd-Warshall. Therefore, all

Powered by Editorial Manager® and ProduXion Manager® from Aries Systems Corporation

metrics based on the calculation of the shortest-paths deal with this.
As a side note, in Pyntacle we have three codes to calculate all the shortest-paths of a graph:
-That borrowed from igraph (sequential)
-That implemented by us using Numba (multi-threading)
-That implemented by us using Numba (GPU)

The Brute-force search, on the other hand, relies on the multiprocess Python package and, as correctly noted, is used to fork processes and not threads. It is used exclusively here because Brute-force search knows the exact number of groups to be computed in advance. Although possible, we have discouraged the mixed-use of processes and threads.

It is not entirely clear, but it seems to me that Figure 4 shows results for using the greedy heuristic with a single thread (no parallelism). Thus it compares the same heuristic algorithm implemented in Pyntacle and R/keyplayer, showing that Pyntacle is faster (due to implementation details, being in Python not R, etc.).

This is a fair comparison if both implementations are really using only a single thread. However I found that Pyntacle used multithreading (up to the number of cores on the node it is run on) automatically, unless the NUMBA_NUM_THREADS environment variable is set (I am not familiar with the Numba library and had to find this in its online documentation). I could not find any documentation in the Pyntacle online manuals about parallelism or adjusting the number of threads, except for the -T/--threads option which applies to the brute-force algorithm only (not the greedy heuristic).
The keyplayer R package, however, uses a single thread unless explicitly requested to use parallelism by a function argument.

Hence I think this should be clarified in the manuscript, and some documentation of how parallelism works and how to adjust the number of threads added to the Pyntacle documentation.

> Since the command-line interface of Pyntacle was designed for not-experts, we decided to hide one level of parallelism (the fine-grained) to the user and to automatically manage the parallelism related to the calculation of the shortest paths. We used this simple algorithm that we will make clever and adaptive in future releases.

```
// auto-select the computing mode
if nprocs > 1  // n.b., nprocs is user-defined
    Let's enable multi-process and disable multi-threading
else if size(graph) < 250 or rho(graph)<0.5  //rho measures sparseness
    Let's disable multi-process and disable multi-threading
else
    Let's disable multi-process and enable multi-threading
```

N.b., we have replaced -T/--threads with -O/--nprocs since it actually refers to the coarse-grained parallelism that forks processes and not threads.

The number of threads of the fine-grained parallelism is internally set to the number of available cpus -1 by default, which can be tuned by setting the env variable NUMBA_NUM_THREADS, as correctly pointed out. However, caution must be paid on this: Numba adjusts the number of active threads on-the-fly according to the current overheads and, hence, the efficiency of parallelism. This means that what specified in the environment variable might not be actually respected. This is the reason why we were not explicit about this aspect. We did not want to cause confusion and frustrating experience to not-expert users.
We have made this clear in the online documentation.

Multi-threading is however controllable via APIs. To actually make fair comparisons, we benchmarked naked functions with code like this:

```
parallelism_test.py
from algorithms.bruteforce_search import BruteforceSearch
from io_stream.generator import PyntacleGenerator
from tools.enums import CmodeEnum, KpposEnum
```

```
if __name__ == '__main__':
    graph_rnd = PyntacleGenerator.Random([100, 0.6])

    start = time.perf_counter()

    # Single-threaded
    mreach_s = BruteforceSearch.reachability(
                graph_rnd,
                2,
                KpposEnum.mreach,
                None,
                m=2,
                cmode=CmodeEnum.igraph, #this enables single-threading
                nprocs=1)  #this is the default choice
    end = time.perf_counter()
    print("--- Elapsed time: {:.2f} seconds ---".format(end - start))
```

Multiprocessing (parallelism on sets) can be enabled via the option nprocs=n as follows:

```
...
BruteforceSearch.reachability(
                graph_rnd,
                2,
                KpposEnum.mreach,
                None,
                m=2,
                cmode=CmodeEnum.igraph,
                nprocs=4)
...
```

Mixing multi-processing with multi-threading is possible, although discouraged since it requires fine-tuning procs/threads ratios to perform well.

```
...
BruteforceSearch.reachability(
                graph_rnd,
                2,
                KpposEnum.mreach,
                None,
                m=2,
                cmode=CmodeEnum.cpu, #this enables multi-threading
                nprocs=3)
...
```

Fig. 5, however, shows the speedup obtained by Pyntacle using the brute-force algorithm with explicit (coarse-grained) parallelism as described, of computing the metric on the independent possible node sets in parallel. This is a valid comparison, however as per A.1.4 above, I question the practicality of the brute-force method at all, given the availability of many more efficient (albeit more complicated, and perhaps requiring a sophisticated ILP solver for example) algorithms for exact solution.

> As in point A.1.4, we agree that there is a broad spectrum of better heuristic search algorithms and optimization techniques than the one currently implemented in Pyntacle. It is our goal to integrate them in the next releases of Pyntacle.
However, in GitHub, we have created a fork (“feat#31) where we have already

implemented the Stochastic Gradient Descent algorithm. This branch is not still merged because this new implementation has to be carefully tested and unittests still need to be done.

A.1.6. Graphics processing unit (GPU).

The manuscript mentions graphics processing units (GPU) for parallelism, but I found no further details on this. When I run Pyntacle on a node with no GPU, I get a warning message that "NVIDIA: no NVIDIA devices found" which would seem to indicate that NVIDIA (CUDA) GPU support is available, and when I run the pyntacle keyplayer module on a cluster node which does have a GPU, I do not get the warning message, but the GPU does not seem to be used (checking with the nvidia-smi program).

Note that I installed the 'miniconda' package on a cluster head node which does not have a GPU (details in Section B below).

> This reviewer is correct. GPU-base processing is an experimental feature at the moment and, as noted by the algorithm above, not covered by the command line. This is because of weird behaviors of Numba with some hardware that might puzzle the user. The GPU feature will be stable in the release 2.0 when we will have covered the possibility to manage big matrices for which replacing fine-grained parallelism with GPU computing would make sense. We have clearly specified this in the documentation.
However, GPU-computing is currently possible by APIs:

```
...
BruteforceSearch.reachability(
        graph_rnd,
        2,
        KpposEnum.mreach,
        None,
        m=2,
        cmode=CmodeEnum.gpu,
        nprocs=1)
...
```

This command applied on a 4000 nodes network makes Pyntacle use just ⅓ of the available GPU capabilities and a not negligible overhead. For this reason, also, we will fully include the GPU option when Pyntacle will be able to handle > 10.000 nodes, efficiently.

A.2. Minor issues

A.2.1. Some notational confusion with 'F'.

The variable F is first mentioned in Case Study 1 (p. 7 "... the best negative key-players in the network (according to both F and DF metrics), ...") but not defined, although it presumably refers to the fragmentation centrality of Borgatti (2006, equation (4)). However it is then used as a variable representing any centrality function (key-player metric) in Algorithms 1 and 2 (p. 9), which could be confusing.
Sorry for this. We have replaced F with FUNC in the algorithms. We have defined Borgatti's F metrics in methods. Thanks.

A.2.2. Some potential confusion for readers between KeyPlayer (Analytic Technologies) and R "keyplayer" package.

At the bottom of p. 5 (column 1): "... keyplayer is not rigorously testable here since it is a Windows-only GUI-based application." This could be confusing as the R keyplayer package (An & Liu, 2016) is previously styled as "keyplayer" (lowercase) while the Analytic Technologies KeyPlayer program (Borgatti, 2019) is styled as "KeyPlayer" ("camel case"), and it is the Analytic Technologies KeyPlayer program that is Windows-only.

Powered by Editorial Manager® and ProduXion Manager® from Aries Systems Corporation

> Amended.

B. Notes on the Pyntacle software
--------------------------------

The Pyntacle website referenced in the manuscript ([http://pyntacle.css-mendel.it)]http://pyntacle.css-mendel.it) is easy to navigate and read, and contains extensive documentation on installation and usage, as well as, importantly, examples and case studies which can be followed and reproduced easily.

I installed the Pyntacle software on a Linux server (the master [head] node of the cluster described below, which has 2 x Intel E5-2630 v3, 16 (2 x 8) cores CPU,64GB DDR4 @ 2133MHz RAM) using the 'miniconda' system as recommended on the Pyntacle home page. This worked without problem, and I ran the tests which passed. I was also easily able to reproduce Case Study 3 (C. elegans connectome) linked from the software home page.

I then ran some of my own tests (using the command line interface, running as batch jobs on cluster compute nodes) to compare results between Pyntacle, the keyplayer (v1.0.3) R package (An & Liu, 2016) and (for KPP-Pos only) the influenceR R package (v0.l.0) (Jacobs et al., 2015).

I came across one bug in the command line interface: the --type m-reach option does not seem to work. But I found this already reported in the GitHub issue tracker as issue #23.

> Amended

Note I ran the tests once only, so the times (which are elapsed times of the key player finding module only, as reported by Pyntacle itself, or system.time() for R packages) are merely indicative. For parallel tests I reserved all 20 cores on a compute node. For serial tests I reserved a single core on a compute node. I set environment variable NUMBA_NUM_THREADS for Pyntacle and OMP_NUM_THREADS for R packages using OpenMP, and the parallel flag and cluster option for the keyplayer R package, to the appropriate number of cores (20 or 1) to ensure the correct number of cores is (potentially) used.

All tests I conducted were on a Linux (CentOS 7.5 x86_64) cluster with 42 compute nodes and Intel 40Gbps QDR Infiniband. The compute nodes used have 2 x Intel Xeon E5-2650 v3 @ 2.30GHz, 20 (2 x 10) cores CPU, 64GB DDR4 @ 2133MHz RAM. The node with a GPU is 2 x Intel Xeon E5-2650 v3 @ 2.30GHz, 20 (2 x 10) cores CPU, 128GB DDR4 @ 2133MHz RAM, GPU:1 x NVIDIA GeForce GTX 1080 Founders Edition 8GB GDDR5X 2560 CUDA cores. The cluster scheduling system is slurm and the R version I used is Microsoft R Open v3.2.5.

Test 1: Key players DF (KPP-Neg) of size k = 2 in the C. elegans connectome (279 nodes), computed with greedy heuristic.

The network data set used is the CAEEL_Connectome.sif file as used in "Case Study 3" from the Pyntacle home page. I used the "pyntacle convert" command to convert it from SIF format to edgelist format for use in the R keyplayers package. Note that the influenceR package only does KPP-Pos so it is not included in this test.

| Method | DF value | Solutions | Elapsed time (s) |
|---|---|---|---|
| Pyntacle serial | 0.58991 | AVAL, AVAR | 18.73 |
| Pyntacle parallel | 0.58991 | AVAL, AVAR | 18.17 |
| R/keyplayer serial | 0.5899131 | AVAL, AVAR | 1940.055 |
| R/keyplayer parallel | 0.5899131 | AVAL, AVAR | 9576.714 |

So this shows Pyntacle computing the KPP-Neg (DF metric) correctly on this test, two orders of magnitude faster than the R keyplayer software. There is no real difference in the time for Pyntacle on 1 core versus 20 cores, perhaps unimportant when it takes

Powered by Editorial Manager® and ProduXion Manager® from Aries Systems Corporation

less than 20 seconds anyway. I'm not sure why the keyplayer R package is so much slower with 20 cores than 1 core, but I suspect it is because the parallel mode in that package is for trying to avoid local minima by running the algorithm with different randomization seeds in parallel (An & Liu 2016, p. 264) rather than speeding the computation of a single run.

In running these tests I found a bug in Pyntacle-Ink (the HTML graphical output): for KPP-Neg metric dF it reports both "Initial Value" and "Value" but they appear to be swapped (they are the right way around in the stdout and the Report TSV file however).

> Amended

Test 2: Key players DR (KPP-Pos) of size k = 2 in the C. elegans PPI network (3303 nodes), computed with greedy heuristic. (This should be the same test as the benchmark shown in Fig. 4 of the manuscript for (D)).

The network data used here is the APID_CAEEL_Level2_maincomponent.adjm adjacency matrix file as used in "Case Study 2" linked from the Pyntacle software home page, and referred to as "APID" in the manuscript.

| Method | DR value | Solutions | Elapsed time (s) |
|---|---|---|---|
| Pyntacle serial | 0.37486 | Q22174, Q95QA6 | 416.70 |
| Pyntacle parallel | 0.37486 | Q22174, Q95QA6 | 353.84 |
| influenceR serial | [not shown] | O45521, Q95QA6 | 1.208 |
| influenceR parallel | [not shown] | Q22174, Q95QA6 | 2.600 |
| R/keyplayer serial | [did not complete within 20 hour elapsed time limit] | | |
| R/keyplayer parallel | [did not complete within 20 hour elapsed time limit] | | |

So it seems to be working correctly, and on my test took about 400 seconds, compared to about 200 seconds in the manuscript Fig. 4, so this seems reasonable. And again, much faster than the keyplayer R package (which could not find a solution within 20 hours).

Again, however, using Pyntacle key player module using the greedy heuristic with 20 cores has only a relatively small advantage over a single core, even though the elapsed time is now several minutes. The Linux (tcsh) time builtin shows 99.8% CPU for the serial run and only 109.0% CPU for the parallel run, showing that little parallelism was used.

> the reason for that might be ascribed to the fact that Numba reduces parallelism when overheads become significant.

However, note that the influenceR R package, which uses a different heuristic algorithm, "stochastic gradient descent" (Jacobs et al., 2015, "keyplayer") is able to find the same solution as Pyntacle, but two orders of magnitude faster (note it also finds a different solution in the serial run - since it is a stochastic algorithm we could get different values each run - but unfortunately does not seem to report the objective value so we cannot see if it is a better or worse solution).

> this note is weak as pointed out by the reviewer him/herself, also because the SGD explores a smaller solution space than the GO algorithm implemented in Pyntacle. In particular:

Stochastic Gradient Descent (SGD)Greedy-optimization search (GO)
1.Select k nodes at random to populate set S
2.Set F = fit using appropriate key player metric (KPP-Pos in our case)
3.Get a new state:
○Pick a random u in S and v not in S.
○F' = fit if u and v were swapped
○If F' > F, swap u and v in S. Else, repeat step 3. (Alternatively, if a positive value is given for the `prob' parameter, a swap will be accepted with a small probability regardless of whether it improves the fit).
If F' - F < tolerance or our maximum computation time is exceeded, return S. Else, go

Powered by Editorial Manager® and ProduXion Manager® from Aries Systems Corporation

to step 3.

(from: https://www.rdocumentation.org/packages/influenceR/versions/0.1.0/topics/keyplayer)
1. Select k nodes at random to populate set S
2. Set F = fit using appropriate key player metric
3. For each node u in S and each node v not in S  <<<<< Loop in red
a. DELTAF=improvement in fit if u and v were swapped
4. Select pair with largest DELTAF
a. If DELTAF <= then terminate
b. Else, swap pair with greatest improvement in fit and set F=F+DELTAF
5. Go to step 3

> SGD adds a random contribution that (can) supersedes the fitting function and saves the loop in red in the GO algorithm. This last fact increases the chance to fall into disadvantageous local maxima, much more than the other algorithm. Even without any deep analysis, out of this scope, it is intuitive why the former is faster than the latter, as it is equally clear that the chances for the former to get the best solution are less than those of the GO algorithm.

Note also that influenceR (despite the short elapsed time) also seems to be making good use of multicore (20 cores): the (Linux) time command shows similar user and elapsed time for the serial run (as expected), but the parallel run shows 26.137 user CPU seconds and only 1.208 elapsed seconds (and only 0.016 system).

Note I also tried to find the key player set size k = 2 for the DF metric (KPP-Neg) with the greedy heuristic in Pyntacle in this data set, but it was unable to complete with the time limit of 20 hours (serial or parallel).

> Pyntacle will do better with more efficient search algorithms in the next releases.

C. Network software libraries/packages referenced
-------------------------------------------------

This list is not necessarily exhaustive, but are some packages that I have used or are aware of, particularly those that are usable from Python. They are all general-purpose graph libraries, with the exception of influenceR and keyplayer, which are specifically for the key players problem. Some have many more features than others though; for example SNAP (Leskovec) handles very large networks efficiently, but has very few algorithms implemented compared to igraph or NetworkX).

| Name | Website | Citation | Interface | Implementation | Parallel | KeyPlayers |
|---|---|---|---|---|---|---|
| graph-tool | https://graph-tool.skewed.de/ | Peixoto (2014) | Python | C++, Boost | OpenMP | No |
| igraph | https://igraph.org/ | Csardi & Nepusz (2006) | Python,R,C/C++,Mathematica | C | No | No |
| influenceR | https://cran.r-project.org/web/packages/influenceR/index.html | Jacobs et al. (2015) | R | R, igraph, SNAP(Bader) | OpenMP | KPP-Pos(DR) |
| keyplayer | https://cran.r-project.org/web/packages/keyplayer/index.html | An & Liu (2016) | R | R, igraph, sna | R/parallel | Yes |
| NetworkX | http://networkx.github.io/ | Hagberg et al. (2008) | Python | Python, SciPy | No | No |
| sna (statnet) | https://cran.r-project.org/web/packages/sna/index.html | Butts (2008) | R | C | No | No |
| SNAP (Bader) | http://snap-graph.sourceforge.net/ | Bader & Madduri (2008); Madduri & Bader (2009) | C | C | OpenMP | No |
| SNAP (Leskovec) | http://snap.stanford.edu/ | Leskovec & Sosič(2016) | Python, C++, NodeXL | C++ | Maybe? | No |

D. References (in addition to those in manuscript)

Powered by Editorial Manager® and ProduXion Manager® from Aries Systems Corporation

-------------------------------------------------


Arulselvan, A., Commander, C. W., Elefteriadou, L., & Pardalos, P. M. (2009). Detecting critical nodes in sparse graphs. Computers & Operations Research, 36(7), 2193-2200.
Bader, D. A., & Madduri, K. (2008, April). Snap, small-world network analysis and partitioning: An open-source parallel graph framework for the exploration of large-scale networks. In 2008 IEEE international symposium on parallel and distributed processing (pp. 1-12). IEEE.
Boginski, V., & Commander, C. W. (2009). Identifying critical nodes in protein-protein interaction networks. In Clustering challenges in biological networks (pp. 153-167).
Butts, C. T. (2008). Social network analysis with sna. Journal of statistical software, 24(6), 1-51.
Celestini, A., Cianfriglia, M., Mastrostefano, E., Palma, A., Castiglione, F., & Tieri, P. (2019). Critical nodes reveal peculiar features of human essential genes and protein interactome. bioRxiv, 831750.
Csardi, G., & Nepusz, T. (2006). The igraph software package for complex network research. InterJournal, complex systems, 1695(5), 1-9.
Csermely, P., Korcsmáros, T., Kiss, H. J., London, G., & Nussinov, R. (2013). Structure and dynamics of molecular networks: a novel paradigm of drug discovery: a comprehensive review. Pharmacology & therapeutics, 138(3), 333-408.
Hagberg, A., Swart, P., & S Chult, D. (2008). Exploring network structure, dynamics, and function using NetworkX (No. LA-UR-08-05495; LA-UR-08-5495). Los Alamos National Lab.(LANL), Los Alamos, NM (United States).
Jacobs, S., Khanna, A., & Madduri, K. Bader D. influenceR: Software Tools to Quantify Structural Importance of Nodes in a Network. 2015. https://cran.r-project.org/package=influenceR [accessed 7 April 2020]. R package version 0.1.0.
Jiang, C., & Liu, Z. (2019). Detecting multiple key players under the positive effect by using a distance-based connectivity approach. Physica A: Statistical Mechanics and its Applications, 534, 122322.
Jiang, C., Liu, Z., Wang, J., Yu, H., & Guo, X. (2017). An optimal approach for the critical node problem using semidefinite programming. Physica A: Statistical Mechanics and its Applications, 471, 315-324.
Lalou, M., Tahraoui, M. A., & Kheddouci, H. (2018). The critical node detection problem in networks: A survey. Computer Science Review, 28, 92-117.
Leskovec, J., & Sosič, R. (2016). Snap: A general-purpose network analysis and graph-mining library. ACM Transactions on Intelligent Systems and Technology (TIST), 8(1), 1-20.
Madduri, K., & Bader, D. A. (2009, May). Compact graph representations and parallel connectivity algorithms for massive dynamic network analysis. In 2009 IEEE International Symposium on Parallel & Distributed Processing (pp. 1-11). IEEE.
McGuire, R. M., Deckro, R. F., & Ahner, D. K. (2015). The weighted key player problem for social network analysis. Military Operations Research, 20(2), 35-53.
Paudel, N., Georgiadis, L., & Italiano, G. F. (2018). Computing critical nodes in directed graphs. Journal of Experimental Algorithmics, 23(2):2.2.
Peixoto, T. (2014). The graph-tool python library. figshare. DOI: 10.6084/m9.figshare.1164194
Schrödinger, LLC. The PyMOL Molecular Graphics System, Version 2.3.3.
Tomaino, V., Arulselvan, A., Veltri, P., & Pardalos, P. M. (2012). Studying connectivity properties in human protein-protein interaction network in cancer pathway. In Data Mining for Biomarker Discovery (pp. 187-197). Springer, Boston, MA.
Ventresca, M. (2012). Global search algorithms using a combinatorial unranking-based problem representation for the critical node detection problem. Computers & Operations Research, 39(11), 2763-2775.
Walteros, J. L., & Pardalos, P. M. (2012). Selected topics in critical element detection. In Applications of mathematics and informatics in military science (pp. 9-26). Springer, New York, NY.


> Irrespective of the success of this submission, we would be happy to keep in touch with this reviewer to get deeper into performance issues and new developments and applications of Pyntacle in ours and other research fields. We are grateful for this precise and in depth review.

Powered by Editorial Manager® and ProduXion Manager® from Aries Systems Corporation

Reviewer #2: In this article, the authors describe Pyntacle, a Python-based software tool for the analysis of networks. Specifically, they focus on identifying groups of nodes by performing a combinatorial evaluation, of various parameters/indices/centrality measures. The manuscript provides a good outline of the package and its capabilities. Their approach is explained well; I found Figure 1 to be a good toy example to illustrate the capabilities of the network. I have a few concerns for the authors to comment upon/implement to further improve their manuscript.

Specific comments:
1. I find the networks in Figure 5 to be very small and not representative of biological networks in complexity, size and density. It would be nice if the examples are changed to 10^3 and 10^4 nodes, discarding 100 nodes. Or, will the combinatorial complexity become a major problem for larger networks?

> As we have shown in the case study, with the network derived from a protein-protein interaction interface, the target of Pyntacle is represented also by small networks (<100 nodes/edges), therefore we wanted to present the different scenarios in which the potential user can find Pyntacle useful, e.g. residue contact or H-bond networks in a complex during a molecular dynamics simulation. Bigger networks (10^3 or 10^4 nodes) would not equally display the same level of informativeness in a Figure given their sizes; moreover, they would not represent a fair comparison ground with the other methods, since already it took more than one day of computation with networks in the 10^3 category, revealing the speedup gain when using Pyntacle. Finally, 10^4-node networks are indeed combinatorially complex to analyze, they will be properly dealt with in the next release of Pyntacle, with a stable GPU implementation and faster heuristic search algorithms and optimization approaches to choose from (as pointed out by reviewer 1 as well).

2. Perhaps, it would be nice if another example, of a protein-protein functional association network from the STRING database is considered. It would be a biologically motivating example, given the opening discussions about protein interaction networks.

> The C.elegans network, collected from APID and used for benchmarking and tests in the manuscript, is built from quality-checked protein-protein interaction data. We clarified this in the text.

3. Could any heuristics be used to cut down the combinatorial search, especially for larger networks. For instance, could nodes with a degree of 1 be ignored? Can the authors suggest some heuristics to simplify computations for larger networks, based on their observations of what nodes typically figure in the "groups".

> The greedy optimization heuristics is shown to identify near-optimal solutions in the manuscript. It represents a fast and simple implementation, even if it is only a starting point. As pointed out by reviewer 1, there is a plethora of faster and more efficient heuristic search algorithms that we aim to include in Pyntacle, so that the user can select different algorithms of choice. (N.b., as a first reaction to this review, we have started and almost finished to implement another search algorithm, the Stochastic Gradient Descent, as it is verifiable in the Pyntacle's GitHub website, branch "feat#31"). We think this is the right path to follow. We do not recommend node (network leaves) pruning as it would greatly influence the outcome of different metrics (for example, the m-reach parameter would yield different results since the removed nodes cannot be reached).

4. `pyntacle test` works very well. Will it be possible to have `pyntacle demo` similarly, which can showcase some of the capabilities? Also, can some example networks be included?

> In order to gradually introduce the user to Pyntacle, we created a quick-start guide and 3 case studies, all detailed with data explanation, command lines and graphs both in the forms of HTML pages and python notebooks. We feel that a Pyntacle "demo" function would not be as welcoming to new users as the guides and case studies. Several examples can be downloaded directly from the quick-start guide/case studies in the website. Moreover, the adjacency matrices associated to the network analyzed

| | |
|---|---|
| | in the two case studies can now be accessed from GigaDB.<br><br>5. An example case study with a larger network, such as E. coli from STRING would also add a lot of value to the paper.<br><br>> The STRING network for C.elegans is one order of magnitude bigger than the E.coli network from the same resource: the STRING C.elegans network counts 18181 nodes (6170 when considering only high-quality interactions with a combined score equal or higher than 900) while the STRING E.coli K12 W3110/MG1655 networks count respectively 4210/4125 nodes (which decrease to 2698/3259 nodes when applying the same high-quality filter). The C.elegans network we used (collected from the APID resource) counts 3303 nodes, same order of magnitude of the STRING networks, and therefore is a suitable representative of this kind of ppi networks. The number of edges greatly varies on the quality of each reported interaction.<br><br>Optional features:<br>1. Lastly, it would be nice if there is a way to pull networks from commonly used databases such as networkrepository.com or STRING. This would enable a lot of network scientists and biologists to easily perform various network analyses.<br><br>> This is a great option to be added in the immediate next release of Pyntacle, the user would be given the choice of selecting among the most used repositories (e.g. STRING, IMEx and APID).<br><br>Typographical errors:<br>Page 1, Para 1: Protein interaction networks, ... represents -> represent<br>Page 1, Para 1: These, -> unclear antecedent -- could we re-written<br>Page 1, Para 3: "having relatively few"" -> "having a few relatively"<br><br>> We corrected these errors. Thanks for this valuable review. |
| **Additional Information:** | |
| **Question** | **Response** |
| Are you submitting this manuscript to a special series or article collection? | No |
| **Experimental design and statistics**<br><br>Full details of the experimental design and statistical methods used should be given in the Methods section, as detailed in our Minimum Standards Reporting Checklist. Information essential to interpreting the data presented should be made available in the figure legends.<br><br>Have you included all the information requested in your manuscript? | Yes |
| **Resources**<br><br>A description of all resources used, including antibodies, cell lines, animals and software tools, with enough information to allow them to be uniquely | Yes |

Powered by Editorial Manager® and ProduXion Manager® from Aries Systems Corporation

| | |
|---|---|
| identified, should be included in the Methods section. Authors are strongly encouraged to cite Research Resource Identifiers (RRIDs) for antibodies, model organisms and tools, where possible.<br><br>Have you included the information requested as detailed in our Minimum Standards Reporting Checklist? | |
| **Availability of data and materials**<br><br>All datasets and code on which the conclusions of the paper rely must be either included in your submission or deposited in publicly available repositories (where available and ethically appropriate), referencing such data using a unique identifier in the references and in the “Availability of Data and Materials” section of your manuscript.<br><br>Have you have met the above requirement as detailed in our Minimum Standards Reporting Checklist? | Yes |

Powered by Editorial Manager® and ProduXion Manager® from Aries Systems Corporation

Manuscript

Click here to access/download;Manuscript;Pyntacle_GigaScience_2020_rev

*GigaScience*, 2020, 1–12

doi: xx.xxxx/xxxx
Manuscript in Preparation
Technical Notes

TECHNICAL NOTES

# Pyntacle: a parallel computing-enabled framework for large-scale network biology analysis

Luca Parca[1], Mauro Truglio[1], Tommaso Biagini[1], Stefano Castellana[1], Francesco Petrizzelli[4,1], Daniele Capocefalo[1], Ferenc Jordán[2], Massimo Carella[3] and Tommaso Mazza[1,*]

[1]IRCCS Casa Sollievo della Sofferenza, Laboratory of Bioinformatics, San Giovanni Rotondo (FG), Italy and [2]Balaton Limnological Institute, Centre for Ecological Research Klebelsberg Kuno 3, 8237, Tihany, Hungary and [3]IRCCS Casa Sollievo della Sofferenza, Laboratory of Medical Genetics, San Giovanni Rotondo (FG), Italy and [4]Department of experimental medicine, Sapienza University of Rome, Rome, Italy

[*]to whom correspondence should be addressed: t.mazza@css-mendel.it

## Abstract

Some natural systems are big in size, complex and often characterized by convoluted mechanisms of interaction, such as *epistasis*, *pleiotropy*, and *trophism*, which cannot be immediately ascribed to individual natural events or biological entities, but that are often derived from group-effects. However, the determination of important groups of entities, like genes or proteins, in complex systems is considered a computationally hard task. Here, we present Pyntacle, a high-performance framework designed to exploit parallel computing and Graph Theory to efficiently identify critical groups in big networks and in scenarios that cannot be tackled with traditional network analysis approaches. We showcase potential applications of Pyntacle with transcriptomics and structural biology data, thereby highlighting the outstanding improvement in terms of computational resources over existing tools.

**Key words**: network; topology; group centrality; systems biology; parallel computing; python

## Background

Interactive systems are commonly represented as graphs (or networks), which are mathematical representations of *elements* (nodes) and their relationships (edges). The semantics of relationships is specific for each graph and completely defines its expressiveness. Protein interaction networks, for example, represents physical interactions as edges and proteins as nodes; metabolic networks wire metabolites ~~if these participate~~ in ~~the same~~ biochemical reactions; regulatory networks are directed graphs, where the directionality of relationships matters. Thus, a link exists between two molecules if there is evidence either of regulatory activity by a transcription factor onto a gene or of post-translational modifications. These, together with several other kinds of networks, like RNA, signaling, neuronal, trophic, and co-expression networks, are the concrete signs of an exceptional growth of molecular interaction data and, hence, of an intense research activity in the field of *Network medicine* [1].

Network medicine is a relatively new discipline that exploits graph theory to identify key-molecules in the human *diseasome* [2] together with their hidden molecular relationships. The general aim is that of reverse-engineering the mechanisms of pathogenesis of complex disorders and traits, whereby the etiology is notoriously convoluted. The diseasome is, in fact, a network where diseases are nodes and links represent relationships between the disease-associated cellular components. Determining such links would help identify the molecular relationships between phenotypes, the reasons of certain comorbidities, and would positively affect diagnosis, treatment and drug multi-purposing.

Certain kinds of biological networks share the feature of

**Compiled on:** July 10, 2020.
Draft manuscript prepared by the author.

**Key Points**

- Understanding the architecture of networks as well as the key-roles of their components is a process that historically relies on the calculation of sets of global and local topological indices.
- The information provided by local topological indices is little or meaningless for natural networks of big sizes or for those that model complex events, like *epistasis*, *pleiotropy*, and *trophism*, which in fact result from the interleaving actions of multiple natural actors.
- Pyntacle provides the user with an array of computationally efficient algorithms to manage networks and to search and find *important* groups of nodes.

having a few relatively ~~few~~ highly connected nodes, often called *hubs*, suggesting that the molecules represented by hubs should play special biological roles. The first hypothesis of Network medicine is that a vast majority of known diseases genes, which are *non-essential*, lie in the periphery of these networks and are far from hubs. On the contrary, at least in human cells, hub molecules are encoded by essential genes [3]. A database, DEG, exists that reports essential genes for some bacteria, archaea and eukaryotes [4]. An interesting speculation is that, because of their many links, hubs are reasonably associated with *disease genes* [5, 6, 7], which in turn, by virtue of the *local hypothesis* of Network medicine, exhibit increased tendency to interact with each other, being them all involved in the same disease. Thus, molecular networks are not random, but tightly organized based on specific principles, according to which the effect of a *central* gene, which is eventually aberrant, reverberates on the gene products of neighboring genes in its network. Hence, the expression of a disease phenotype does rarely result from an individual aberrant gene, rather from the harmonized effects of groups of related genes. This holds true also for other types of networks, ranging from ecological, to evolutionary and chemical networks.

Graph theory draws upon various tools to identify the most central elements, i.e. the key molecules, in a network. Here, the concept of centrality is synonymous with importance, even if it has been seen to decline differently in literature. A topologically important node may be a hub, a *bottleneck*, namely a node that lies in many pathways, or one that is "close" to most other nodes. But for what said above, local (i.e., regarding nodes or edges) and global (i.e., regarding the entire network) properties of networks are unlikely to completely explain the functioning of complex systems, since they do not take into account or underestimate the effects that groups of important nodes may jointly exert on these systems. Node 2 in Figure 1 has 7 ties and it is the highest *degree* node in this example network. It is connected with 7 unimportant nodes, since these exhibit low degree values. Node 9 is the second more connected node with only one less edge than node 2, but two of its neighbors, i.e., node 16 and 21, are the third and fourth ranked nodes by degree with 5 and 4 ties, respectively. Thus, although node 2 is top ranked by degree, it may not be the most functionally central node. Whether this assertion is true or not strictly depends on the purposes by which a network is being studied.

More interestingly, the *network parsimony* principle of Network medicine, according to which *causal molecular pathways often coincide with the shortest molecular paths between known disease-associated components*, implies that it is fundamental to find the nodes that lie within the highest number of pathways in networks, since these are more likely to be functionally critical [1]. The *betweenness* centrality index [8] is the most suitable for this task. Node 21 in Figure 1 is the top ranked node by betweenness. This was expected since it lies in the exact middle of the network, that in turn, exhibits a quasi-tree topological node organization. But even if node 21 belongs to almost all

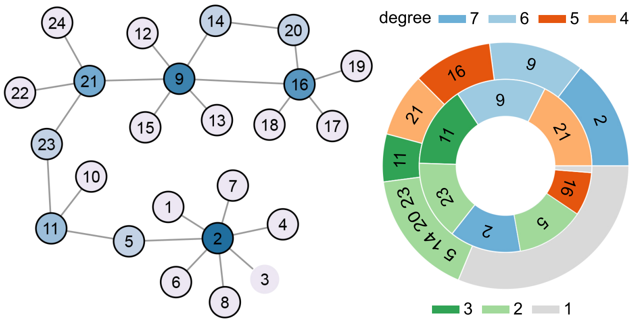


**Figure 1.** (left) Example network. The darker the blue color, the higher the degree of nodes. (right) Pie chart of the most central nodes. The outer circle reports the highest degree nodes (counterclockwise, blue through gray). The inner circle represents the highest betweenness nodes, from light orange to gray, counterclockwise. Node names are reported within circle sectors. Sector width is proportional to the degree (outer) and betweenness (inner) values of nodes. Gray sectors contain unimportant nodes, i.e. nodes with unitary degree and negligible betweenness values.

**Table 1.** Group centrality metrics calculated for the example network. Higher scores indicate higher centrality.

| Group | Degree | Betweenness | Closeness* |
|---|---|---|---|
| {2, 21} | 0.5 | 0.39 | 0.58 |
| {2, 9} | 0.59 | 0.43 | 0.69 |
| {21, 9} | 0.36 | 0.35 | 0.38 |
| {2, 9, 21} | 0.71 | 0.45 | 0.75 |

* The *minimum* method was used to measure the distance from the group to an outside node.

shortest paths of the network, it ranks only fourth by degree, since it is not individually much connected. Node 9 is the second node by betweenness with a score very close to that of node 21 but, on the contrary, it ranks second by degree (Supplementary data S1). Whether the most important node is 2, 21 or node 9 depends on the aims and context of the study.

Whenever more than one node exhibits similar topological scores, as in this case or when a co-responsibility for a phenotype is suspected, studying groups and their centrality might be a reasonable option. In 1999, Everett and Borgatti expanded the definition of degree, betweenness and *closeness* to groups of nodes [9]. Calculating these indices for the following groups: {2, 21}, {9, 21}, {2, 9}, the latter achieved the highest scores. Moreover, considering the group made by all three nodes, only degree and closeness increased significantly in respect to {2, 9} (cf. Table 1).

In 2006, Borgatti introduced other two classes of metrics for groups that were meant to assess the ability of groups either to disrupt a network, when removed, or to efficiently spread *information* through a network. These were defined as “Key-Player Problem/Negative” (KPP-Neg) and “Key-Player Prob-

lem/Positive" (KPP-Pos), respectively [10]. N.B. similar concepts were also covered in other research fields and scientific contexts [11, 12], where specific search strategies [13, 14] were implemented. KPP-Neg and KPP-Pos were calculated for the same groups and reported in Table 2. It is interesting to notice that {2, 9} is still the most important group in terms of disruption potential and connectivity. Their scores were slightly lower than those of group {2, 9, 11}, meaning that even here node 11 does not contribute significantly to the centrality of {2, 9}.

**Table 2.** KPP-Neg and KPP-Pos metrics calculated for the example network. DF (Neg) achieves its maximum value of 1.0 when the graph consists entirely of isolated nodes. M-reach (Pos) is a count of the number of unique nodes reached by any member of the group in *m* links or less. DR (Pos) achieves a maximum value of 1 when every non-group node is adjacent to at least one member of the group.

| Group | DF (0.66) | m-reach* | DR |
|---|---|---|---|
| {2, 21} | 0.87 | 79.2% | 0.65 |
| {2, 9} | 0.91 | 95.8% | 0.72 |
| {21, 9} | 0.84 | 62.5% | 0.53 |
| {2, 9, 21} | 0.93 | 95.8% | 0.74 |

* The *m* parameter of the algorithm was set to 2. The percentage of nodes reached by the group, including the group nodes, is reported.

What remains to be verified is whether any other group exists that exhibits similar or higher centrality values. Considering the small network size, the option of running a *brute-force* algorithm to search the absolute best group(s) among all possible ones is computationally feasible, in place of a *greedy-optimization* search, as suggested by Borgatti in [10]. In this case, the best group of size 2 for all metrics is still {2, 9}, whereas {5, 9} reaches 100% of non-group nodes and ranked first by m-reach. However, since none of the centrality scores of {2, 9} equaled their maximum possible values, we applied again the brute-force search to groups of increasing sizes, 3 through 6. We thus found that degree and closeness reached their absolute maximum scores, i.e. 1, equally with two groups {2, 9, 11, 16, 21}, {2, 9, 10, 16, 21} of size 5, meaning that nodes 10 and 11 are interchangeable and equally important; betweenness obtained its maximum score (0.497) with the group {2, 9, 11, 16, 21} (Supplementary data S2). The best group by DF is {2, 9, 11, 14, 16, 21}, which achieves the score of 1. The groups {2, 9, 10, 16, 21} and {2, 9, 11, 16, 21} equally obtained the best DR score (0.792). It is worth noticing that DR and betweenness do not reach their absolute maximum scores, which however are plausibly the highest possible for this network, since groups of bigger sizes exhibit lower scores (Figure 2). It is also interesting to notice that nodes 2, 9 and 21 are included in all groups found above, thereby highlighting their central roles in the network (Supplementary data S3).

Computing the *nestedness*, which consists in verifying whether sets of nodes recur in groups of increasing sizes, could confirm the importance of nodes 2, 9 and 21. Hence, if larger sets contain smaller sets, higher values of nestedness may be a proxy for identifying upstream/master regulators through the key-nodes of the smallest groups. One way to calculate the nestedness of the example network is by the *Nrow* metrics [15, 16]. Nrow is defined as the average percentage of nodes from smaller sets that are contained in larger sets, taking all possible pairs of sets. Thus, after computing all the best sets of increasing sizes, from 2 to 5, for each group centrality metrics but *m-reach*, nodes 2 and 9, and not 21, resulted to be nested in all sets, regardless of their size (Figure 3 and Supplementary

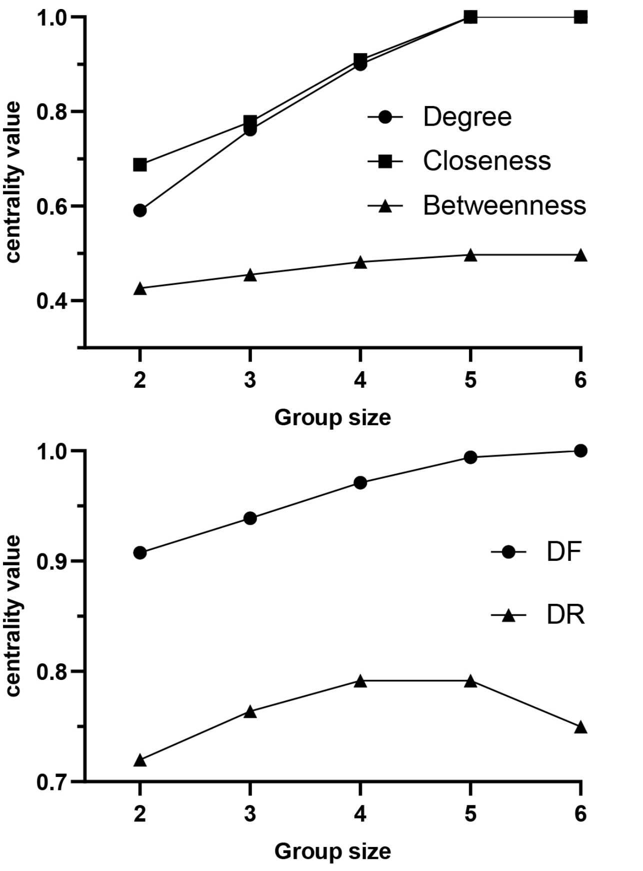


**Figure 2.** Brute-force search algorithm applied to all groups of sizes 2 through 6. For any size, the maximum score obtained for (top) group-degree, group-closeness, group-betweenness, (bottom) DF and DR are plotted.

data S2). The same evidence emerged with the key-player metrics (Supplementary data S3). The nestedness scores were generally quite high, meaning that nodes are not interchangeable among groups, i.e., there are few equally important nodes. The group {2, 9} is definitely important from a topological point of view and its discovery would have not been immediately hypothesized without this investigation, since the nodes 2 and 9 are five links apart.

This "practical" introduction aims at introducing the theory underlying Pyntacle. A toy-model was used to describe the main features, outline a possible analytical pathway and highlight how Pyntacle may help extract valuable information from real-world networks. The rest of the article will thus present i) the software and its main components, ii) its design and implementation, iii) how to finely use it; iv) benchmarks, assessed on real and simulated networks of increasing sizes, in comparison with a similar software package; v) two real-world case studies.

## Pyntacle

Pyntacle is an open-source network analysis framework that was originally designed to tackle the Borgatti's Key-Player Problem [10] efficiently through the identification of maximally reachable or disruptive groups of nodes. Contrary to similar tools that either analyze networks with standard global and local topological metrics [17, 18], or that provide the users with limited tools to detect important groups of nodes [19], Pyntacle adopts optimized heuristic algorithms and parallel computing strategies to make the task of identifying key-player nodes feasible. It has the following attributes: i) available for Windows,

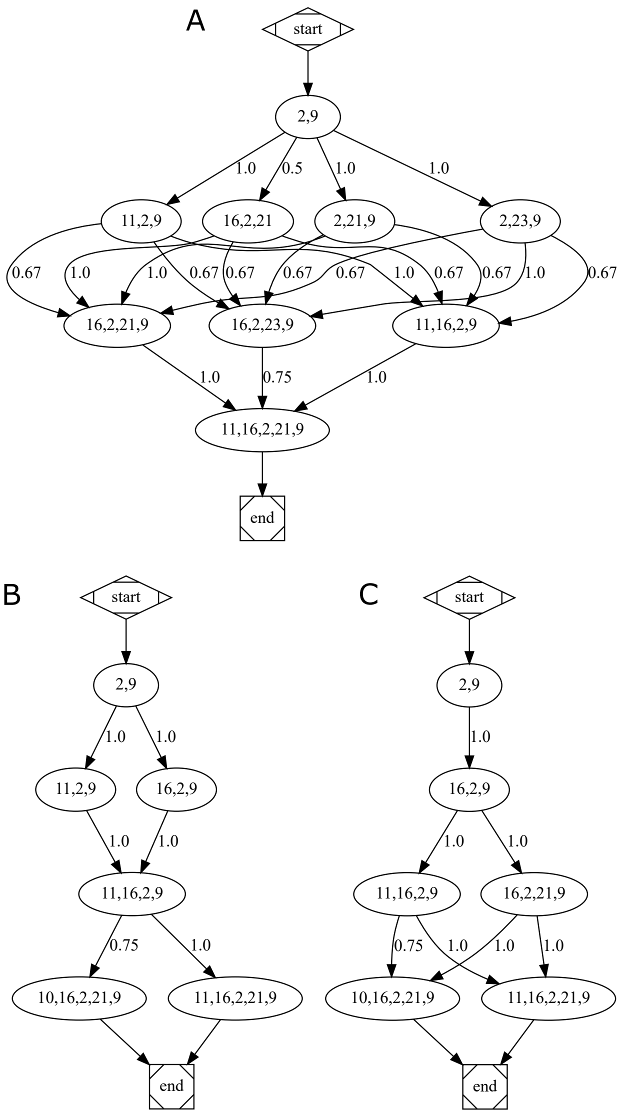


**Figure 3.** Nestedness graphs for group- (A) *betweenness*, (B) *closeness*, (C) *degree* centrality metrics. Nodes represent groups with top centrality values in respect to all other possible groups of nodes with same sizes. Edges connect groups when the bigger group contains at least one element of the smaller one. Edges are labeled with the overlap ratio between the elements of the connected groups.

Mac, and Linux OS; ii) available as both command line tool and API, with an easy and user-friendly interface for both input commands and results visualization; iii) it allows the management of real-world graphs in a computationally efficient way.

Pyntacle is implemented in modules, each designed to analyze a particular aspect of a network. These can calculate global and local topological metrics (*metrics* module), the importance of groups of nodes (*groupcentrality* and *keyplayer* modules), search and analyze clusters of nodes (*communities* module), perform set operations between networks (*set* module), generate networks with different topological organizations (e.g. random, scale-free and small-world networks, *generate* module), convert and load/save networks using different data formats (e.g. adjacency matrix, edge list, SIF and dot, *convert* module).

## Features

### *Centrality measures for groups*

Pyntacle tackles the problem of identifying key-player nodes that, together, optimally diffuse *something* through a network or maximally disrupt or fragment a network when removed. It further extends the standard network centrality measures of *degree*, *closeness*, and *betweenness* (refer to [20] for a clear introduction and to [21] for further theoretical explanations) to groups rather than individual elements. To this regard, these methods are a direct generalization of the corresponding individual measures, in a way that if, e.g., *group-degree* and *degree* are applied to groups consisting of single elements, they yield identical results. The class of algorithms are thus two: one that measures the importance of a set on the basis of its impact on the remaining nodes of a network, and another that does it by considering the sole properties of the elements of a set.

The former class is composed by the DF (KPP-Neg; cf. Eq. 2 in Methods), DR (cf. Eq. 3) and m-reach (KPP-Pos; cf. Eg. 4) algorithms [10]. KPP-Neg measures the fragmentation of a network because of a set. KPP-Pos measures the overall cohesion that members of a set have with the remainder of the network. As described in the Methods section, DF measures the degree of *reachability* of a set of nodes, taking also into account the degree of cohesion of the set. M-reach counts the number of unique nodes reached by any member of a set in *m* links or less. DR is the weighted proportion of all nodes reached by the set, where nodes are inversely weighted by their minimum distance from the set.

The latter class is made by the *group-degree* centrality measure that accounts for the number of non-group nodes that are connected to group members (cf. Eq. 5 in Methods); the *group-betweenness* centrality measure that measures the proportion of (shortest) paths connecting pairs of non-group members that pass through the group (cf. Eq. 6); the *group-closeness*, which sums the distances from the group to all vertices outside the group (cf. Eq. 7).

### *Search strategies for optimal sets*

When the aim is not to quantify the centrality of a specific set of nodes, but that of discovering which is/are the most central set(s) in a network, the search heuristics might come in handy. In particular, Pyntacle implements a *greedy optimization* search heuristics presented in [10] and a *brute-force* combinatorial optimization search strategy (cf. *Search algorithms* section in Methods). The former progressively replaces the components of a starting random set with all other nodes of a graph, calculating one of the formerly mentioned centrality metrics for that group, and then stops when a sub-optimal solution is obtained. The latter loops through all possible groups of a predefined size and returns only those exhibiting the best scores for any of the centrality measure. It is immediate that the computational complexity of the heuristic method is much lower than that of the exact method, at the cost of sub-optimal solutions. The brute-force search yields exact solutions, but is computationally impracticable for big networks.

### *Exploration of crosstalk pathways of sparse real-world networks*

Real-world biological networks exhibit hierarchical organizations, where subnetworks (e.g., signaling pathways) are bridged by *crosstalk* links [22]. A number of developmental processes rely on crosstalks, where their aberrant regulation was found to be associated with inflammatory response defects as well as cancer and neurodegeneration [23, 24]. Together with the observation that causal molecular pathways often coincide with the shortest molecular paths between known disease-associated components (cf. the *network parsimony* principle [1]), these render the study of crosstalks in networks fundamental.

Pyntacle eases the exploration of crosstalks by *set operations* on graphs. Individual networks can thus be compared (union, intersection and difference) or merged and then studied topologically.

These networks are typically sparse and can be analyzed employing algorithms that work best with graphs with a few edges. Pyntacle is optimized to work with increasingly large and complex networks. It provides the user with the possibility to assess the extent of sparseness of a network though mathematical indices, including the *compactness* and *completeness* indices [25, 26]. In addition, it chooses the best implementation of computationally heavy algorithms at run-time (e.g., the search for all the shortest paths), according to the available hardware (i.e., single or multi-core processors and GPU-enabled graphics cards) and some network global metrics, including the *sparseness*.

### *Data format compatibility and reporting*

Pyntacle is compliant with the Cytoscape's *SIF* data format and with the *dot* network data format. It can input and output *adjacency matrices*, *edge lists* as textual files as well as serialized binary Python objects. Graph, node, or edge attributes can be imported/exported from/to file.

Pyntacle can report any analysis result in two formats: as textual files and as rich HTML files. In particular, the *PyntacleInk* module outputs an interactive, automatically generated web page that displays the graph, its attributes, and all the results of the analyses that were performed on it.

## Implementation

Pyntacle is accessible via command line and exposes a Python API for fine-tuning its algorithms. It depends on iGraph [17] for handling the graph data structure and borrowing some basic local and global topological measures and network generators.

Heavy computations of new algorithms are just-in-time compiled to native machine instructions by Numba [27] and thus run on multi-process CPU or NVIDIA-compatible GPU hardware, if available in the hosting computing infrastructure (experimental feature only accessible through APIs in version 1.2). Differently from similar packages, this allows Pyntacle to process graphs with thousands of nodes, thus helping it manage, for example, the whole human transcriptome and other networks of comparable sizes. Moreover, GPU-acceleration provides high-speed computing of the Pyntacle's algorithms, thereby making heavy and long-running tasks feasible.

The *PyntacleInk* visualizer exploits HTML5, Javascript and Sigma to produce an interactive representation of the input graph, its base metrics, and a graphic rendering of the results of most of Pyntacle's algorithms (KPP, group centrality, graph generation, set operations, community detection, Figure 6B). A graph can be displayed using different layouts (i.e., *Random, Circular, ForceAtlas, Fuchterman-Reingold*), and the canvas renderer allows the visualization and smooth interaction with graphs up to 5000 nodes in size, using a web browser of a standard desktop PC. All the information about a graph and the analyses that were performed on it are stored in a JSON file; this dictionary is updated with new information whenever a new run of analysis is performed on the same graph, allowing the user to simultaneously explore the results of different algorithms and - through the use of timestamps - the results of the same algorithm run with different parameters over time. Any graphical representation can be exported as vector graphics (SVG) or PNG screenshots.

Finally, Pyntacle is fully compatible with the Jupyter Notebook.

## Benchmarks

Compared with the keyplayer 1.0.3 R package [28] and KeyPlayer 1.44 [29], Pyntacle has the following attributes: i) available for Windows, Mac, and Linux OS; ii) available as both command line tool and API; iii) it allows management of real-world graphs in a computationally efficient way.

Wall-clock-time (WCT) comparisons of Pyntacle and keyplayer, when searching for optimal kp-sets of some real and simulated graphs, are shown in Figure 4. Noteworthy is that ~~keyplayer~~KeyPlayer is not rigorously testable here since it is a Windows-only GUI-based application.

Random networks were generated according to the Erdős-Rényi model. Six random networks, three with 100 nodes and three with 1000 nodes, were generated. These two groups of networks differed for their wiring probability, which varied as 0.3, 0.5 and 0.7. This probability is a kind of weighting function, which ranges from 0 to 1, with bigger numbers producing denser networks. Other four real networks were used: the network representing strong advice-seeking ties in global consulting company from [10] (32 vertices and 55 edges); the parasite-host food web of the Carpinteria Salt Marsh Reserve (128 vertices and 1198 edges) [15]; the *C. elegans* connectome (a modified version of the network published in [30], 279 vertices and 1960 edges) and a high-quality *C. elegans* ~~PPI~~protein-protein interaction network (3303 vertices and 5561 edges, downloaded from APID [31], the Agile Protein Interactomes DataServer [32]).

WCT were measured three times for each network and centrality algorithm. DR, m-reach and DF were the only three algorithms in common between the two software packages. The sub-optimal sets of size 2 were determined by both software using their own implementations of the greedy optimization search algorithm (cf. *Search algorithms* in the Methods section). Starting from the 100-nodes random networks, Pyntacle computed all indices in fractions of seconds (or a few seconds for DF), irrespective of the wiring probability. keyplayer computed the same indices of the same networks in 4 to 9 minutes. Considering the 1000-nodes networks, keyplayer completed the computation of all indexes in more than one day, while Pyntacle took a few minutes to 5 hours (DF). Similarly, real networks were analyzed in fractions (or tenths for DF) of seconds by Pyntacle and in a few seconds to 1 hour by keyplayer, which took more than 1 day to analyze the APID network, as opposed to Pyntacle that ran for a few minutes to 17 hours. Generally, Pyntacle was 40 to 3,900 times faster than keyplayer, depending on the test.

The brute-force search algorithm yields exact solutions at the cost of an intrinsic combinatorial complexity. However, its computational load can be split into parallel processors. In Pyntacle, the best solutions are obtained after the enumeration of all possible groups of nodes and the calculation of their topological indices. Calculations are in fact independent from each other and hence suitable to be executed in parallel. When applied to our test networks with the aim to calculate the DR index, we verified that the smaller ones ($\leq$ 100 nodes) have benefited from parallel execution only limitedly. While the *strong advice-seeking ties in global consulting company* network exhibited the best *speed-up* with the employment of 4 computing cores (1.76X), before decreasing its performance, the net execution time improvement consisted in fact only of 82 msec, on average (Fig. 5C). Similarly, 100-nodes random networks, proportionally to the rewiring probability, achieved the best speedup values with 16 cores (~8X) with an improvement of just ~4 sec (Fig. 5B). As expected, bigger networks benefited from parallel execution increasingly with the number of nodes. The Carpinteria network achieved the best speedup record (~11X) with 16 cores, although saving just 7 sec. of com-

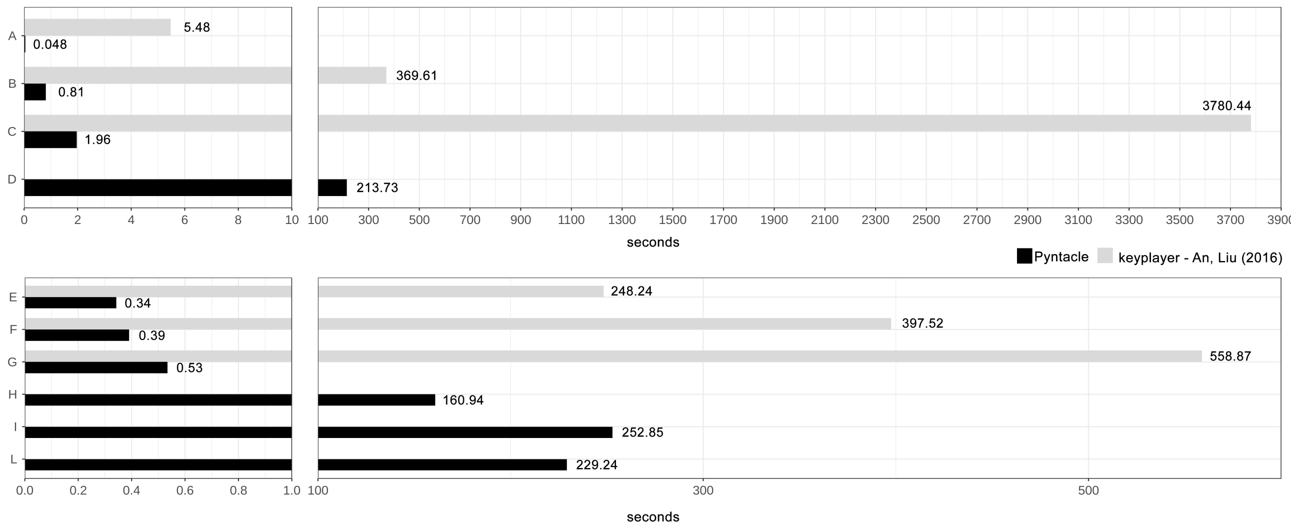


**Figure 4.** Greedy-optimization search, metrics: DR. (A) Strong advice-seeking ties in global consulting company (Borgatti, 2006); (B) Parasite-host food web of the Carpinteria Salt Marsh Reserve (Capocefalo, et al., 2018); (C) *C. elegans* connectome; (D) High quality *C. elegans* PPI network (APID); Erdős–Rényi random networks with (E-G) 100 nodes and rewiring probability p = 0.3, 0.5 and 0.7; (H-L) 1000 nodes and p = 0.3, 0.5 and 0.7.

putation, while the Connectome peaked at ~25X with 32 cores. (Fig. 5A). The computations of 1000-nodes random networks scaled well up to 16 nodes, exhibiting comparable speedups of ~7X, ~6X,~6X, when varying the rewiring probability from 0.3 to 0.5 and 0.7, respectively. The bigger APID network exhibited the best performance with 32 cores, achieving a speedup of ~29X and terminating the computation 23 hours earlier than the non-parallel run (Fig. 5D).

Although these are far from being linear speedups, the advantage and efficacy of parallel computing strategies is evident for networks of big sizes. These results can be reproduced using a Docker image available from the Pyntacle website.

## Analyses

### Case Study 1 - protein-protein interaction interface

NADH dehydrogenase [ubiquinone] flavoproteins 1 and 2 (NDUFV1 and NDUFV2) are two core subunits of the mitochondrial respiratory Complex 1 [33]. Their interaction is mediated by 138 interface residues (Figure 6A).

We have built a network whose edges linked interacting residues of the two proteins with the aim to identify key residues at the interface between the two proteins and whose mutations might significantly affect their interaction (Figure 6B). Thus, we computed several local topological metrics for these residues, e.g. *degree*, *betweenness*, *closeness*, *radiality* and a few others, but none of them showed to correlate appreciably with the contribution provided by each residue (Supplementary Figure S1) on the NDUFV1-NDUFV2 interaction energy ($\Delta\Delta G$, expressed in Kcal/mol and calculated with FoldX [34], see Methods): a maximum Pearson correlation of 0.32 was observed between $\Delta\Delta G$ and *betweenness*.

We then applied Pyntacle to the network, searching for the best positive and negative key-player sets of size 2 (colored in blue and red, respectively, in Figure 6, Supplementary data S4). The residues Glu161 and Tyr46 of NDUFV1 were identified as the best negative key-players in the network (according to both F and DF metrics), namely their removal was estimated to maximally fragment the network and thus potentially hamper the interaction between the two proteins. This was further confirmed by their energetic contributions to the interaction when mutated to Alanine ($\Delta\Delta G$ +0.9 and +3.7 Kcal/mol, respectively for Glu161 and Tyr46). Moreover, Glu161 of NDUFV1, paired with Leu234 of NDUFV2, and Cys125 of NDUFV1, paired with Tyr46, were identified as the best positive key-players pairs (respectively calculated with the DF and m-reach metrics), namely they resulted to be immediately reachable from the remaining network by direct links or indirect links joining close neighbor residues.

Contrary to Glu161 and Tyr46, Leu234 and Cys125 neither exhibited a significant $\Delta\Delta G$ when mutated to Alanine, nor were characterized by high values of local topological metrics. For these reasons, they would have been overlooked by all other techniques, even the more accurate and computationally intensive, as the Alanine-scanning [35]. Other than being computationally demanding and impracticable for large-scale analyses, Alanine-scanning is known to be blind to residues which are chemically similar to Alanine, thereby ignoring their epistatic features which are critical in some regions of the interaction interface.

All these issues are overcome with Pyntacle, which allows to look for topologically important groups of residues between proteins efficiently and regardless of their chemical structure.

### Case Study 2 - miRNA-miRNA interaction network

microRNAs (miRNAs) are small RNA molecules (18-25 nucleotides) able to regulate gene expression levels through different cellular mechanisms, the most important of which is that a miRNA can recognize different messenger RNAs (mRNAs) as targets and, at the same time, one of those targets can be recognized by multiple miRNAs. Due to the renowned role played by miRNAs in tumorigenesis and cancer progression [36], we have analyzed a miRNA interaction network of patients affected by breast cancer.

Expression data of healthy and tumor tissue samples of 87 patients affected by breast cancer were retrieved from The Cancer Genome Atlas (TCGA) and were used to wire 487 miRNAs in two correlation networks, built on healthy and tumor samples, respectively. A functional association between any two miRNAs was assumed to exist if the absolute values of the Pearson's correlation coefficients of their expression levels exceeded 0.5 (cf. *Case Study 2* in Methods).

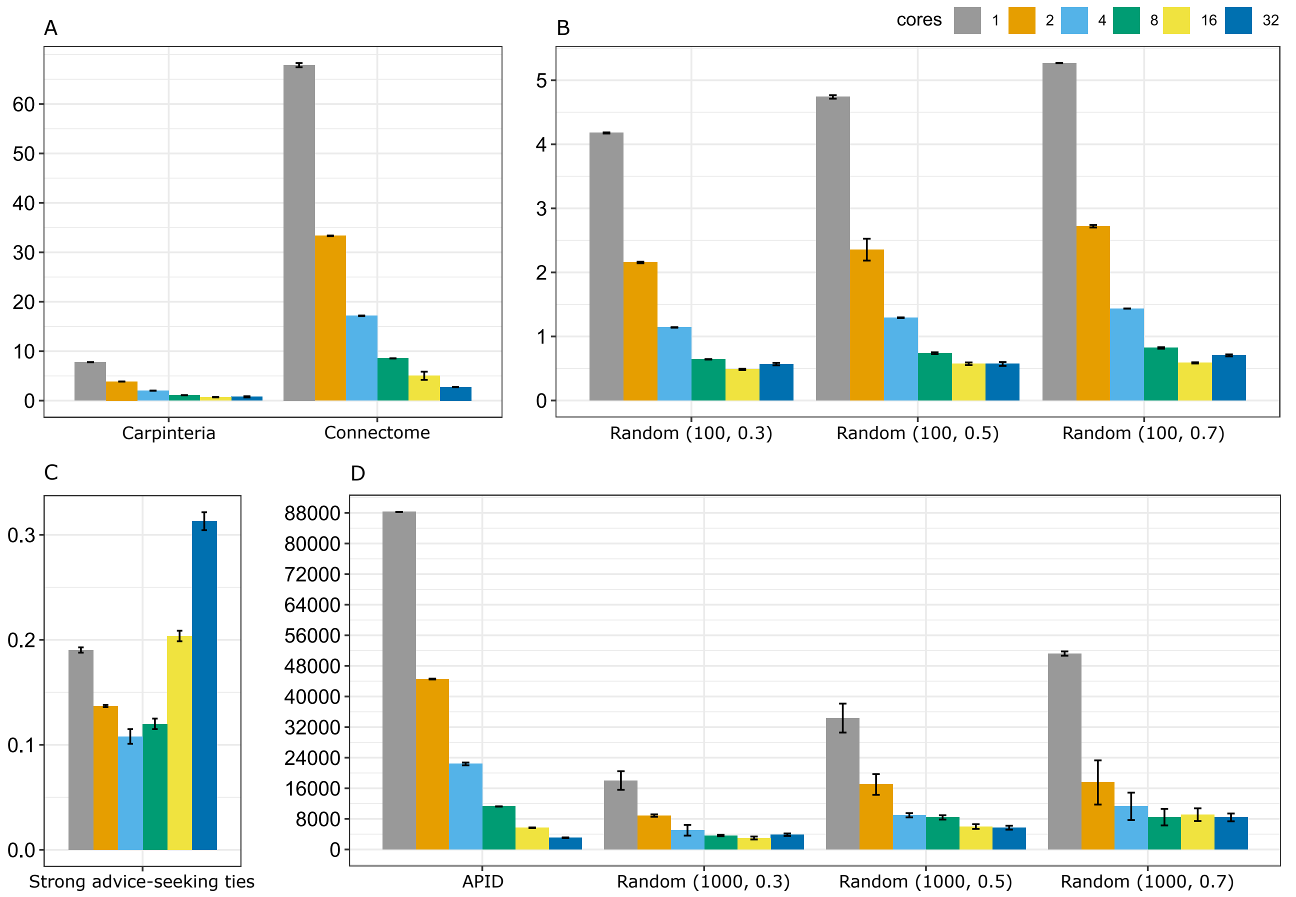


**Figure 5.** Improvement of execution times (in seconds) of Pyntacle using parallel computing on different networks using increasing numbers of computing cores.

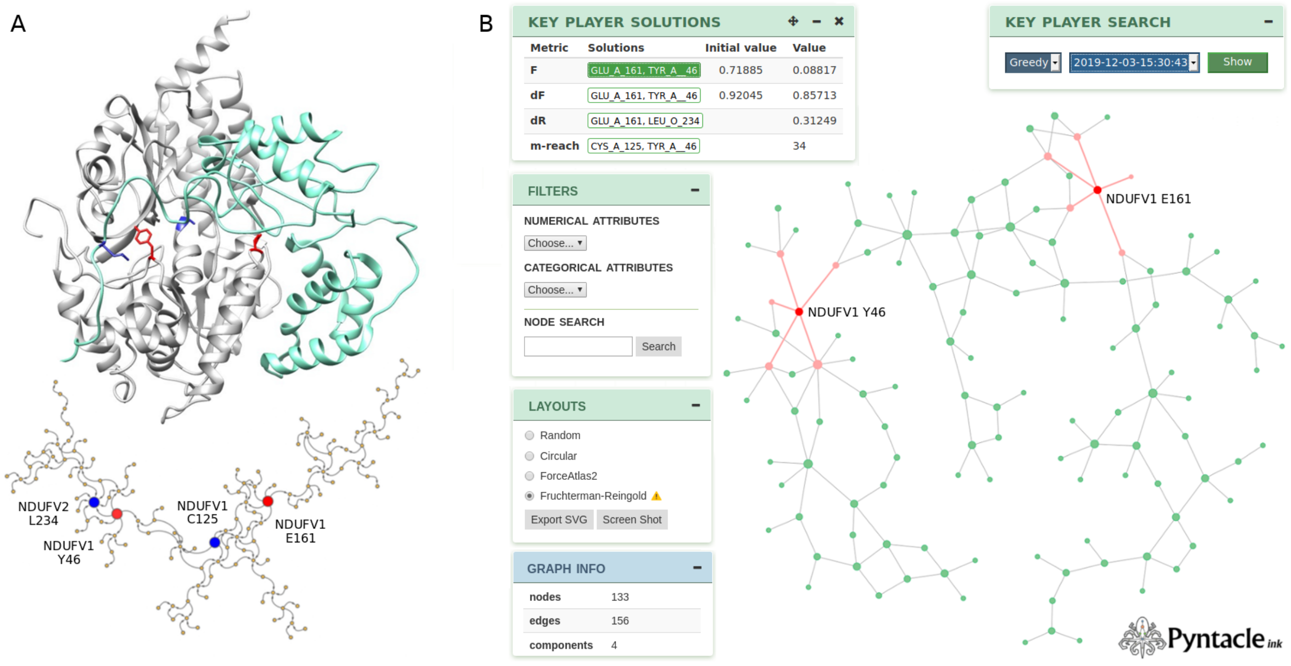


**Figure 6.** A: Representation of the interaction between NDUFV1 and NDUFV2, colored in white and cyan respectively (PDB id 5xtd). The ~~138~~ residues forming the interaction interface are represented as a graph (bottom) connecting residues close in space. Positive and negative key-players are colored in blue and red respectively in both the interaction structure and interaction network. B: The PyntacleInk viewer. Different menus can be used to i) visualize all the analyses that have been performed on a network, ii) visualize the network general metrics, iii) filter nodes by attributes and iv) change the overall layout of the network.

The healthy network was very dense and highly connected (average degree of 40.4), as opposed to the tumor network which was mostly disconnected (average degree of 17.4). Both networks were analyzed with Pyntacle, which identified miR-

1307-3p and miR-140-3p, as negative key-players and miR-136-5p, miR-484 and miR-127-5p, as positive key-players of the healthy tissue network. These are all associated with the onset, development of breast cancer and some are even used as markers for prognosis [37, 38, 39, 40, 41, 42] (Supplementary data S5).

In the tumor network, Pyntacle identified miR-192-5p, miR-483-5p and miR-577 as negative key-players and miR-324-5p and miR-337-3p as positive key-players, all involved in *proliferation*, *cell migration* and *metastasis* of breast cancer [43, 44, 45, 46, 47] (Supplementary data S6).

It has to be noted that while these miRNAs have individually high betweenness values, it would not have been possible to infer a possible synergistic interaction between them without the key-player analysis of Pyntacle.

## Discussion & concluding remarks

The motivations behind Pyntacles come from the constant growth of experimental data sets and from the increasing need to represent and analyze them with computational efficient methods. This first release of Pyntacle has been designed with these aims and thus to help researchers from different scientific fields and with different levels of computing skills to approach Network Biology and benefit from its analytical tools.

We showed the attributes of Pyntacle and its versatility in dealing with different problems, but all traceable to possibly big networks of interactive elements. In a former case study, Pyntacle was thus able to identify key-amino acids that were greatly contributing to the formation of a protein-protein interaction interface. This otherwise time-consuming task was accomplished very efficiently by translating the problem into a network analysis task, compared to current approaches that take tens of minutes to handle even small interaction interfaces. In a second case study, Pyntacle was used to analyze the TCGA data set of miRNA expression in breast cancer to build miRNA-miRNA networks. These were analyzed in search of microRNAs that were occupying key positions in the network, which were later discovered to be already known biomarkers or responsible for the onset and progression of breast cancer.

Concluding, Pyntacle represents a starting point for large-scale Network Biology studies. Being a modular framework, it will be expanded to handle *weighted* networks, in the near future, and *directed* networks, immediately after. These features, which are shared with some other key-player detection methods and tools [48, 49, 28], are relevant to make Pyntacle fully capable of analyzing all kinds of biological networks. It will also be enriched with new optimization search algorithms and with a new algorithm to compute the set *nestedness*. New applications and use-cases are envisaged, the currently most concrete is the one concerning the analysis of trajectories of Molecular Dynamics simulation of proteins.

## Potential implications

The main problem concerning most of the currently available network analysis tools, which is also the main reason why we made Pyntacle, is that these do not handle networks of medium to big sizes efficiently. This issue not only regards the big networks and the suitability and effectiveness of the currently available algorithms to analyze them, but also their practicability over a reasonable time axis and in terms of required computing resources. This point is critical for most fields of research, from Biology to Medical and Social sciences, where systems are naturally big and complex (e.g., the whole *Proteome*, the *Diseasome* and the *Socialnomics* to mention a few). In particular, a field that at the time of this writing is hitting the headlines, i.e. Epidemiology, is greatly developing in terms of the capability to draw contagion maps and predict infection growth over time. These maps are actually networks where nodes are people and edges are relationships occurred in recent and short periods of time. The way these networks could be studied are many. One could be that of determining the front of the infection, namely a group made by healthy people that are *close* to the affected people and *highly social* and to administer a vaccine, in order to curb the infection. Another possibility would be that of determining the minimum possible number of communication routes to be closed at a national level in order to implement a clever lock-down. These and several other options may be implemented in Pyntacle, using ad-hoc algorithms and computing protocols tailored for big networks.

## Methods

### Key-Player and group-centrality metrics

Pyntacle tackles the problem of identifying key-player nodes that, together, optimally diffuse *something* through a network or maximally disrupt or fragment a network when removed. The classes of algorithms are thus two: one that measures the importance of a set on the basis of its impact on the remaining nodes of a network, and another that does it by considering the sole properties of the elements of a set. The former class, also known as KPP-Neg, measures the fragmentation of a network because of the removal of a set of nodes. It is composed by the F metrics:

$$F = 1 - \frac{\sum_k s_k(s_k - 1)}{n(n-1)} \tag{1}$$

which bases on the size $s_k$ of its components $k$; and by the DF metrics:

$$DF = 1 - \frac{2\sum_{i>j} \frac{1}{d_{ij}}}{n(n-1)} \tag{2}$$

where $d_{ij}$ denotes the distance between the $i_{th}$ and the $j_{th}$ node. It ranges from 1, when all nodes are adjacent to 0, when all nodes are *isolates*.

The latter class, also known as KPP-Pos, measures the overall cohesion that members of a set have with the remainder of the network and is made by:

$$DR = \frac{\sum_j \frac{1}{d_{Kj}}}{n} \tag{3}$$

where $n$ is the size of the graph and $d_{Kj}$ denotes the minimum distance (shortest path) between any member $i$ of the set $K$ and the remaining nodes $j$ in the graph. Similarly, the *m-reach* metrics counts how many unique nodes can be reached from $K$ in $m$ steps or less. The formulation is:

$$C_K = \sum_{j \in V \setminus K} \cup \frac{1}{d_{ij}} \tag{4}$$

where $C_K$ ranges from 0 to $n - k$, $n$ representing the size of the graph and $k$ that of the considered set of nodes. It is important to notice that this index assumes that all paths of length $m$ or less are equally important and that all paths longer than $m$ are wholly irrelevant [10].

Pyntacle further extends the standard network centrality measures of *degree*, *closeness*, and *betweenness* to groups of nodes, in a way that if, e.g., *group-degree* and *degree* centrality measures are applied to groups consisting of single elements, they yield identical results. This class of metrics is made by the *group-degree* centrality measure, which is intended as the number of non-group nodes that are connected to group members. Multiple ties to the same node are counted only once. It is defined as:

$$GD_K = \frac{\sum_{i \in K, j \in V \setminus K} a_{ij}}{\|V \setminus K\|} \tag{5}$$

where $a_{ij}$ equals 1 when $i$ and $j$ are adjacent nodes, if $i \in K$ and $j \in \{V \setminus K\}$, and counting $a_{i,j}$ and $a_{v,j}$ only once $\forall v \in K$ when $a_{i,j} = 1$ and $a_{v,j} = 1$. Hence, the group-degree centrality ranges from 0 to 1, if the group $K$ is completely isolated or fully connected to all other nodes. *Group-betweenness* centrality of a set $K$ is defined as the number of shortest paths connecting any two nodes $u$ and $v$ passing through $K$ over the number of all paths between the two.

$$GB_K = \frac{p_{u,v}(K)}{p_{u,v}} \tag{6}$$

where $u$ and $v$ are any pair of nodes not belonging to the group $K$, $p_{u,v}(K)$ represents the number of shortest paths connecting $u$ and $v$ and that traverses $K$, while $p_{u,v}$ is the total number of shortest paths between $u$ and $v$. *Group-closeness* of a group $K$ is defined as the sum of the *minimum, maximum, average* distances from the nodes belonging to the group to all other nodes outside the group.

$$GC_K = \frac{\sum_{j \in V \setminus K} \bar{d}_{Kj}}{\|V \setminus K\|} \tag{7}$$

where $\bar{d}_{ij}$ is the minimum, maximum or average distance between nodes in $K$ and all other nodes.

### Search algorithms

When the aim is not to quantify the centrality of a specific set of nodes, but to discover which is/are the most central set/s of nodes in a network, search heuristics might come in handy. In particular, Pyntacle implements a greedy optimization search heuristics presented in [10], and a brute-force combinatorial optimization search strategy. The former follows this naïve algorithm:

**Algorithm 1** Greedy optimization search

```
procedure greedySearch(k: int, V: list, F: lambda)
    score = 0.0
    scoredict = {} ← hashmap
    halt = false
    K = DRAWNODES(k, V)
    while not halt do
        foreach u ∈ K do
            foreach v ∈ V \ K do
                Ktemp = SWAP(K, u, v)
                scoretemp = FUNC(Ktemp)
                scoredict[(u, v)] = scoretemp
        u_best, v_best = GETPAIRSWITHBESTSCORE(scoredict)
        bestscore = scoredict[(u_best, v_best)]
        if best_score > score then
            K = swap(K, u_best, v_best)
            score = bestscore
        else
            halt = true
      return (K, score)
    end
```

FUNC is an appropriate key-player metrics; `drawnodes` is a function that randomly picks $k$ nodes from $V$, which contains all nodes of the network; `swap(K,u,v)` substitutes the element $u$ in $K$ with $v$; `getPairsWithBestScore` is a function that returns the pairs which yielded the best centrality score. This method progressively replaces the components of a starting random set $K$ with all other nodes of a graph, calculating one of the formerly mentioned centrality metrics for that group and then stops when a sub-optimal solution is obtained.

The brute-force combinatorial optimization search strategy implemented in Pyntacle loops through all possible groups of a predefined size ($k$) and returns only those exhibiting the best scores for any of the previous centrality measure ($F$). Even if

the computation can be performed in parallel (the **foreach** loop below), it is immediate that the computational complexity of the heuristic method is much lower than that of this method, at the cost of yielding sub-optimal solutions. The algorithm below returns exact solutions, but is computationally impracticable with big networks.

**Algorithm 2** Brute-force search

```
procedure bruteforceSearch(k: int, V: list, F: lambda)
    bestscore = 0.0
    scoredict = {} ← hashmap
    allsets = GENERATECOMBINATIONS(k, V)
    foreach set ∈ allsets do
        scoretemp = FUNC(set)
        scoredict[set] = scoretemp
    bestsets = GETSETSWITHBESTSCORE(scoredict)
    bestscore = scoredict[bestsets]
    return (bestsets, bestscore)
    end
```

GENERATECOMBINATIONS is a function that generates all possible sets of nodes of size $k$ picking nodes from $V$.

## Set operations on graphs

Graph *union*, $G1 \cup G2$, is implemented as $(V1 \cup V2, E1 \cup E2)$, namely as the union of nodes ($V$) and edges ($E$). Graph *intersection* is defined as $G1 \cap G2 = (V1 \cap V2, E1 \cap E2)$, where only common nodes and edges are reported in the resulting graph. The *difference* between $G1$ and $G2$ results in a graph with nodes and edges only present in $G1$ and not in $G2$. Since the difference between graphs is not reciprocal, $G1 - G2 \neq G2 - G1$.

## Case study 1

Chains $A$ and $O$ of the PDB structure `5xtd` were considered for the analysis of NDUFV1 and NDUFV2, respectively. First, the structure has been repaired (*RepairPDB* module of FoldX) and thus allowing more relaxed residue side-chain rotamers and solving clashes. Then, residues located at the interaction interface and the interaction energy were determined with the *AnalyseComplex* module of FoldX. Alanine-scanning of the interface residues was performed by substituting each amino acid with an Alanine residue (*BuildModel* module). The interaction energy of each mutant has been calculated with the *AnalyseComplex* module in order to determine the $\Delta\Delta G$ of the mutant compared to the wild-type interaction structure. A residue-residue interaction network has been built, which connected residues, belonging to different chains in the complex, if any of their non-hydrogen atoms (both backbone and side-chain) were in a 4.5Å radius from each other. The network has been then analyzed with the *keyplayer* module of Pyntacle and a greedy-search algorithm was used to find the optimal key-player sets of size 2, considering all available metrics (F and DF, as KPP-Neg. and DR and m-reach, with m set to 2, as KPP-Pos).

## Case study 2

Expression levels of 547 miRNAs in 87 healthy and 87 tumor breast samples were retrieved from TCGA. Separately for healthy and tumor individuals, correlations of expression between any possible pairs of miRNAs (149, 331 total pairs) were calculated by Pearson correlation coefficient. Only significant values that exceeded $\pm 0.5$ were considered to represent edges connecting miRNAs in the *healthy* and *tumor* networks. In both networks, the best KPP-Pos and KPP-Neg sets of size 2 were sought using the Pyntacle's greedy optimization search and calculating F and DF, as KPP-Neg, and DR and m-reach, with m set to ~~2~~1, as KPP-Pos.

## Availability of source code and requirements

- Project name: **Pyntacle**
- Project home page: `http://pyntacle.css-mendel.it`
- Operating systems: **Linux, Mac and Windows**
- Programming language: **Python 3.6+**
- Other requirements: **CUDA toolkit** (optional)
- License: e.g. **GNU GPL 3.0**

Source code is stored in GitHub. Installation procedures, tutorials, case studies, a Docker container are all available from the Pyntacle's website.

## Declarations

### List of abbreviations

- **KPP-Neg**: Key-Player Problem/Negative
- **KPP-Pos**: Key-Player Problem/Positive
- **APID**: Agile Protein Interactomes
- **TCGA**: The Cancer Genome Atlas
- **RNA**: Ribonucleic Acid
- **mRNA**: messenger RNA
- **miRNA**: microRNA
- **NADH**: Nicotinamide Adenine Dinucleotide - Hydrogen (reduced)
- **API**: Application Programming Interface
- **WCT**: Wall-clock-time

### Competing Interests

The authors declare that they have no competing interests.

### Funding

This study was supported by the Italian Ministry of Health (Ricerca Corrente 2020 RC20DS-COMU) and by the "5x1000" voluntary contribution. Conflict of Interest: none declared.

### Author's Contributions

LP designed and performed the experiments; MT implemented PyntacleInk and took care of the package maintenance and deployment; DC implemented the command line interface, the set operations, wrapped the iGraph modules and performed the benchmarks; TB, SC and FP contributed to the case study analysis; MC contributed to the definition of the case studies; FJ oversaw the implementation of the topology metrics; TM designed and implemented the software and oversaw the project.

## Acknowledgements

We are grateful to Christoph Gohlke, from the Laboratory for Fluorescence Dynamics, University of California, for providing Windows unofficial binaries of the iGraph Python extension package; Juliana Pereira for testing and constructing discussions and to NVIDIA Corporation for supporting this research. The results of case study 2 shown here are in whole or part based upon data generated by the TCGA Research Network.

## References

1. Barabasi AL, Gulbahce N, Loscalzo J. Network medicine: a network-based approach to human disease. Nat Rev Genet 2011;12:56–68.
2. Goh KI, Cusick ME, Valle D, Childs B, Vidal AL M Barabási. The human disease network. Proc Natl Acad Sci USA 2007;104:8685–8690.
3. Jeong H, Mason SP, Barabási AL, Oltvai ZN. Lethality and centrality in protein networks. Nature 2001;411:41–42.
4. Luo H, Lin Y, Gao F, Zhang CT, Zhang R. DEG 10, an update of the Database of Essential Genes that includes both protein-coding genes and non-coding genomic elements. Nucleic Acids Research 2014;42:D574–D580.
5. Wachi YK S, Wu R. Interactome-transcriptome analysis reveals the high centrality of genes differentially expressed in lung cancer tissues. Bioinformatics 2005;21:4205–4208.
6. Jonsson PF, Bates PA. Global topological features of cancer proteins in the human interactome. Bioinformatics 2006;22:2291–2297.
7. Xu J, Li Y. Discovering disease-genes by topological features in human protein–protein interaction network. Bioinformatics 2006;22:2800–2805.
8. Freeman LC. A Set of Measures of Centrality Based on Betweenness. Sociometry 1977;40:35–41.
9. Everett MG, Borgatti SP. The centrality of groups and classes. The Journal of Mathematical Sociology 1999;23(3):181–201. `https://doi.org/10.1080/0022250X.1999.9990219`.
10. Borgatti SP. Identifying sets of key players in a social network. Computational & Mathematical Organization Theory 2006;12:21–34.
11. Boginski V, Commander CW. Clustering Challenges in Biological Networks. Springer; 2009.
12. Csermely P, Korcsmáros T, Kiss HJM, London NR G. Structure and dynamics of molecular networks: A novel paradigm of drug discovery: A comprehensive review. Pharmacology & Therapeutics 2013;138:333–408.
13. Lalou M, Tahraoui MA, Kheddouci H. The Critical Node Detection Problem in networks: A survey. Computer Science Review 2018;28:92–117.
14. Walteros JL, Pardalos PM. Applications of Mathematics and Informatics in Military Science. Springer Optimization and Its Applications, vol. 71. Springer; 2012.
15. Capocefalo D, Pereira J, Mazza T, Jordán F. Food Web Topology and Nested Keystone Species Complexes. Complexity 2018;2018.
16. Almeida-Neto M, Guimarães P, Guimarães Jr PR, Loyola RD, Ulrich W. A consistent metric for nestedness analysis in ecological systems: reconciling concept and measurement. Oikos 2008;117.
17. Csardi G, Nepusz T. The igraph software package for complex network research. InterJournal Complex Systems 2006;1695.
18. Hagberg A, Swart P, Chult DS. Exploring network structure, dynamics, and function using NetworkX. Los Alamos National Lab (LANL), Los Alamos, NM (United States) 2008;p. LA-UR-08-05495; LA-UR-08-5495.
19. Jacobs S, Khanna A, Madduri K, Bader d, influenceR: Software Tools to Quantify Structural Importance of Nodes in a Network; 2015. `https://cran.r-project.org/package=influenceR`.
20. Borgatti SP, Everett MG. A Graph-theoretic perspective on centrality. Social Networks 2006;28:466–484.
21. Freeman LC. Centrality in networks: I. Conceptual clarification. Social Networks 1979;1:215–239.
22. Vert G, Chory J. Crosstalk in Cellular Signaling:

Background Noise or the Real Thing? Dev Cell 2011;21(6):985–991.
23. Zolezzi JM, Inestrosa NC. Wnt/TLR Dialog in Neuroinflammation, Relevance in Alzheimer's Disease. Front Immunol 2017;24(8):187.
24. Qu X, Tang Y, Hua S. Immunological Approaches Towards Cancer and Inflammation: A Cross Talk. Front Immunol 2018;20(9):563.
25. Menniti S, Castagna E, Mazza T. Estimating the global density of graphs by a sparseness index. Applied Mathematics and Computation 2013;224:346–357.
26. Mazza T, Romanel A, Jordán F. Estimating the divisibility of complex biological networks by sparseness indices. Briefings in Bioinformatics 2010;11(3):364–374.
27. Crist J. Dask & Numba: Simple libraries for optimizing scientific python code. In: 2016 IEEE International Conference on Big Data (Big Data); 2016. p. 2342–2343.
28. An WH, Liu YH. An R Package for Locating Key Players in Social Networks. The R Journal 2016;8(1):257–268.
29. Borgatti SP, KeyPlayer 1.44; 2019. [Online; accessed 06-August-2019]. http://www.analytictech.com/keyplayer/keyplayer.htm.
30. Towlson EK, Vértes PE, Ahnert SE, Schafer WR, Bullmore ET. The Rich Club of the C. elegans Neuronal Connectome. Journal of Neuroscience 2013;33(15):6380–6387. https://www.jneurosci.org/content/33/15/6380.
31. Alonso-López D, Campos-Laborie FJ, Gutiérrez MA, Lambourne L, Calderwood MA, Vidal M, et al., Agile Protein Interactomes DataServer; 2019. [Online; accessed 07-August-2019]. http://apid.dep.usal.es/.
32. Alonso-López D, Campos-Laborie FJ, Gutiérrez MA, Lambourne L, Calderwood MA, Vidal M, et al. APID database: redefining protein-protein interaction experimental evidences and binary interactomes. Database 2019 01;2019. https://doi.org/10.1093/database/baz005.
33. Guo R, Zong S, Wu M, Gu J, Yang M. Architecture of Human Mitochondrial Respiratory Megacomplex I2III2IV2. Cell 2017;170:1247–1257.
34. Schymkowitz J, Borg J, Stricher F, Nys R, Rousseau F, Serrano L. The FoldX web server: an online force field. Nucleic Acids Res 2005;33:W382–W388.
35. Weiss GA, Watanabe CK, Zhong A, Goddard A, Sidhu SS. Rapid mapping of protein functional epitopes by combinatorial alanine scanning. Proc Natl Acad Sci U S A 2000;97:8950–8954.
36. Reddy KB. MicroRNA (miRNA) in cancer. Cancer Cell International 2015;15(38).
37. McGuire A, Brown JAL, Kerin J. Metastatic breast cancer: the potential of miRNA for diagnosis and treatment monitoring. Cancer Metastasis Rev 2015;34:145–155.
38. Wang DY, Gendoo DMA, Ben-David Y, Woodgett JR, Zacksenhaus E. A subgroup of microRNAs defines PTEN-deficient, triple-negative breast cancer patients with poorest prognosis and alterations in RB1, MYC, and Wnt signaling. Breast Cancer Res 2019;21(18).
39. Pronina IV, Loginov VI, Burdennyy AM, Fridman MV, Senchenko VN, Kazubskaya TP, et al. DNA methylation contributes to deregulation of 12 cancer-associated microRNAs and breast cancer progression. Gene 2017;604:1–8.
40. Li Q, Yao Y, Eades G, Liu Z, Zhang Y, Zhou Q. Downregulation of miR-140 promotes cancer stem cell formation in basal-like early stage breast cancer. Oncogene 2014;33:2589–2600.
41. Vos S, Vesuna F, Raman V, van Diest PJ, van der Groep P. miRNA expression patterns in normal breast tissue and invasive breast cancers of BRCA1 and BRCA2 germ-line mutation carriers. Oncotarget 2015;6:32115–32137.
42. Wang X, Zhu J. Mir-1307 regulates cisplatin resistance by targeting Mdm4 in breast cancer expressing wild type P53. Thorac Cancer 2018;9:676–683.
43. Wang Z, Wang J, Yang Y, Hao B, Wang R, Li Y, et al. Loss of has-miR-337-3p expression is associated with lymph node metastasis of human gastric cancer. Journal of Experimental & Clinical Cancer Research 2013;32(76).
44. Zuo XL, Chen ZQ, Wang JF, Wang JG, Liang LH, Cai J. miR-337-3p suppresses the proliferation and invasion of hepatocellular carcinoma cells through targeting JAK2. Am J Cancer Res 2018;8:662–674.
45. Yin C, Mou Q, Pan X, Zhang G, Li H, Sun Y. MiR-577 suppresses epithelial-mesenchymal transition and metastasis of breast cancer by targeting Rab25. Thoracic Cancer 2018;9:472–479.
46. Cioce M, Valerio M, Casadei L, Pulito C, Sacconi A, Mori F, et al. Metformin-induced metabolic reprogramming of chemoresistant ALDHbright breast cancer cells. Oncotarget 2014;5:4129–4143.
47. Li JY, Jia S, Zhang WH, Zhang Y, Kang Y, Li PS. Differential Distribution of microRNAs in Breast Cancer Grouped by Clinicopathological Subtypes. Asian Pacific Journal of Cancer Prevention 2013;14:3197–3203.
48. Paudel N, Georgiadis L, Italiano G. Computing Critical Nodes in Directed Graphs. Journal of Experimental Algorithmics 2018;23:2.2.
49. McGuire RM, Deckro RF, Ahner DK. The Weighted Key Player Problem for Social Network Analysis. Military Operations Research 2015;20:35–53.

Supplementary Material (6 xls sheets)

Click here to access/download
**Supplementary Material**
Supplementary Data S1-S6.xls

Supplementary Figure S1

Click here to access/download
**Supplementary Material**
Supplementary Figure S1.pdf

Cover and rebuttal letter
Click here to access/download;Personal Cover;Pyntacle_rev1_rebuttal.pdf

*Bioinformatics laboratory*
IRCCS Casa Sollievo della Sofferenza
bioinformatics@css-mendel.it
http://bioinformatics.css-mendel.it

Rome, July 10th 2020

Dear Dr. Zauner,
We have revised our manuscript replying to all reviewers concerns at our best. In particular:
- We have faced the problem of reproducibility, by submitting the definition files of the networks used for our case studies to GigaDB. One of the curators confirmed that a link to these files will be available in case of acceptance of the manuscript.
- We have clarified that the Brute-force algorithm, although clearly disadvantageous, can still be useful for case studies encompassing small/medium size networks. This is one possibility to have exact solutions to the search of optimal sets of nodes. As clarified, Pyntacle makes use of fine and coarse-grained parallelism. While the latter is exclusively used with the brute-force implementation, the former is implicitly applied to large-scale networks with the aim to ease their analysis.
- We are particularly grateful to this reviewer, because he/she has helped a lot in identifying critical performance issues and improve the clarity of our presentation. We have tackled this point seriously and answered accordingly.
- We have updated our references, also following the reviewer's suggestions

Following this cover letter, the reviewers will find a point-to-point answer to their concerns. Hope they and yourself will appreciate our efforts to make Pyntacle as much appealing, catching and intuitive as possible. All authors have participated in the work and take full public responsibility for the content of the paper.

Yours sincerely

Tommaso Mazza, PhD
Bioinformatics Laboratory, head
Casa Sollievo della Sofferenza - Mendel
Viale Regina Margherita 261 - 00198 Roma IT

Tel: +39 06 44160526 - Fax: +39 06 44160548
E-mail: t.mazza@css-mendel.it
Web page: http://www.css-mendel.it/
Web page: http://bioinformatics.css-mendel.it

*Bioinformatics laboratory*
IRCCS Casa Sollievo della Sofferenza
bioinformatics@css-mendel.it
http://bioinformatics.css-mendel.it

**Reviewer #1**: "Pyntacle: a parallel computing-enabled framework for large-scale network biology analysis" GigaScience manuscript (Technical Note) GIGA-D-20-00087

This manuscript describes a software library for graph computations usable as both a Python library and command line interface. In addition, it describes an interactive viewer output (Pyntacle-Ink) module for visualizing the results. Although Pyntacle is a general-purpose graph library, it has a particular focus (as does the manuscript) on group centrality metrics and the "key player problem" (KPP), which is the computationally intractable problem of finding sets of (rather than individual) nodes in a network which maximize (or minimize) a group centrality metric.

This distinguishes it from other general-purpose network packages, which usually do not include algorithms for the KPP, while those packages that do address it tend to be specific to that problem, and not general purpose libraries (see section C below).

Another distinguishing feature of Pyntacle is that it is targeted particularly at large-scale network biology analysis, and emphasizes parallel computing. Although the KPP is well-known in social network analysis (from which it originated), it is not so well-known in biology (although not unknown, see A.1.1 below) and so this manuscript makes a contribution in introducing it (and making available usable multi-platform software in a platform widely used in bioinformatics, i.e. Python) to the field.

A. Notes on the manuscript
--------------------------
The manuscript is generally clear, giving a (quite lengthy) introduction and exposition of the KPP, description of the software and algorithms implemented, benchmark results, and two case studies.
The software (both source code and installation packages), documentation, and data sets required for reproducibility are all referenced and freely available from the project website (although see A.1.3 below for an exception).

A.1. Major issues
These are points I think must be addressed before the manuscript can be accepted.

A.1.1. Insufficient references to existing research literature.
The KPP originated in social network research with the "key player problem" so named by Borgatti (2006). However in other fields, and specifically computer science and applied mathematics (operations research for example), the KPP and variants are known as "critical element detection" or the "critical node problem" (CNP) (Walteros & Pardalos, 2012), or the "critical node detection problem" (CNDP), of which the CNP is a variant (Lalou et al. (2018)).

This is an active research field, as evidenced by the review paper Lalou et al. (2018), and although a review of this field is clearly outside the scope of this manuscript (technical note), at least a brief mention of the relationship of the KPP to the CNDP, and a citation of (for example) the Lalou et al. (2018) review paper would be of value to readers and not leave them with the misleading impression that the KPP has only been studied in a couple of papers and only the brute-force and greedy heuristic algorithms have been studied to solve it.

As well as this general issue, I think some references to the prior use of the KPP (or related CNDP variants) in biology would be welcome, for example in protein-protein interaction (PPI) networks (Boginsnki & Commander, 2009; Tomaino et al., 2012), and genetic regulatory networks (Celestini et al., 2019). For a more general perspective on network methods in drug discovery, the extensive review of Csermely et al. (2013) could be informative.

In the concluding remarks (p. 8) where extension to weighted and directed networks is mentioned, it might be noted that some work exists on the CNDP in directed graphs (Paudel et al., 2018) and KPP on weighted graphs (McGuire et al., 2015; Yang, 2015; Jiang & Liu, 2019). Indeed the R keyplayer package is claimed to work with both directed and weighted networks (An & Liu, 2016).

Although reproducing results from the many algorithms for solving the KPP or CNDP variants (either exactly i.e. to optimality, or to potentially suboptimal solutions with heuristics) is clearly impractical, rough comparisons to other methods could be facilitated by running the benchmarks on the Critical Node Detection Problem Benchmark Instances (Ventresca 2012), available from http://individual.utoronto.ca/mventresca/cnd.html, which are used in many CNDP papers.

We thank the reviewer for the suggested reads, we integrated them in the revised manuscript. As far as the proposed benchmark is concerned, we think that it would be very informative and useful. However, we prefer to postpone this to the next future for two reasons:
1- as the reviewer has noticed, Pyntacle is equipped with just one heuristic method. We think that the suggested effort would be much more effective to take when Pyntacle will run several more algorithms. This will happen with the next major release, scheduled to come out sometimes in the second half of the next year.
2- we are aware that those presented are preliminary benchmarks and we were struggling with whether to present them or not. We would prefer to keep them at this preliminary level or, eventually, completely removing them, if suggested by the reviewers.

A.1.2. Insufficient references to existing software.
Although a review of existing network libraries is beyond the scope of the manuscript, it could be useful for readers to at least mention some other general purpose graph libraries usable from

Python such as graph-tool (Peixoto, 2014), igraph (Csardi & Nepusz, 2006), NetworkX (Hagberg et al., 2008), and SNAP (Leskovec & Sosič, 2016). (See Section C below for more).

Most particularly, it is noted that Pyntacle uses igraph modules, yet the igraph paper (Csardi & Nepusz, 2006) is not cited.

In addition, although the Analytic Technologies KeyPlayer program and the R keyplayer package (An & Liu, 2016) are mentioned, the influenceR R package (Jacobs et al., 2015) is not mentioned. Although this package only solves KPP-Pos with the DF metric, as I show in Section B below (on a single example) it does so much faster than Pyntacle.

We integrated the references in the revised manuscript.

A.1.3. Reproducibility (data availability) of case studies.

For Case Study 1, I was easily able to reproduce the 3D protein cartoon in Fig. 6 by downloading the 5XTD structure from the RCSB PDB into PyMOL (Schrödinger, LLC), selecting the appropriate two chains, and locating the key player residues as shown. However to obtain the network of "The 138 residues forming the interaction interface" I would (according to the Methods) have to conduct several operations in FoldX and then extract the network according to a distance threshold described in the Methods. Although it seems there is sufficient detail to reproduce this, it requires a registration to use FoldX and some further work construct the network.

I think it would assist reproducibility (an important consideration in the GigaScience journal) to supply the network as constructed by the authors as a Supplementary Information file (as for example the results of the Pyntacle analysis are already supplied in an Excel file).

Similarly, the network constructed for Case Study 2 could also be made available as Supplementary Information.

We have stored the adjacency matrices of the two case studies in GigaDB. N.b., A member of the GigaScience editorial team confirmed that a link to these files will be made available eventually after the publication of this piece of research. Moreover, we have explained how we made them in order to improve replicability.
In fact, in Case Study 1, the 138 residues identified via Foldx are those considered at the interface and for which energetic contributions were calculated. The network we built has been obtained by directly analyzing the PDB file of the complex, connecting residues of different chains, and relying only on distance constraints (therefore avoiding external software like FoldX). As we demonstrate in the case study, valuable information is gained nonetheless, without using external software (FoldX) and in much less time.

A.1.4. Use of "brute-force" algorithm and "large-scale".

The manuscript is described (including in the title) as being for "large-scale" network analysis, and parallelism is emphasized. However (related to point A.1.1 above) it seems to me that the emphasis is perhaps misplaced. To be specific, two algorithms are implemented and benchmarked: the "brute-force" algorithm, and the greedy heuristic algorithm of Borgatti (2006). Parallelism then mainly seems to be discussed in relation to the brute-force algorithm.

However, as the KPP is known to be computationally intractable and indeed NP-complete (Arulselvan et al., 2009), a brute-force algorithm is never going to be practical, and certainly not for large-scale instances. For this reason there are two practical approaches: (a) exact solution by more sophisticated methods (for example integer linear programming (ILP), semidefinite programming (Jiang et al., 2017), branch-and-cut methods, etc.), and (b) heuristic algorithms to find a potentially suboptimal solution, but much faster. Note that the latter are also vital as input into the former (as bounds on the solution). For a review of these approaches see Lalou et al. (2018).

Hence my concern is that implementing and applying parallel computation to the brute-force approach is misplaced, as it can never be competitive with sophisticated exact solution algorithms (particularly where these could also be parallelized) on large-scale problems.

Regarding the heuristic (greedy) algorithm, it also seems that better results could be obtained with a more sophisticated heuristic, as already foreshadowed by Borgatti (2006). For example I demonstrate in Section B below, that the influenceR program is two orders of magnitude faster (albeit on a single example) than Pyntacle for KPP-Pos, as it uses a different heuristic algorithm. In addition there are many publications showing superior results with different heuristic algorithms for CNDP variants (see Lalou et al., 2018 for a review).

BF was implemented because it is exact and without aiming to compete with any other search algorithm. The fact that we implemented some parallelism for it is because some fields of research, like ecology, deal with small/medium size networks, where size is in the order of a few tens/hundreds of nodes. Why should we yield suboptimal results for these when exact is achievable?
We were not clear enough on this: parallelism was applied to BF (coarse+fine grain) as well as on heuristics (fine grain only). Fine-grain parallelism applies directly to the way we enumerate shortest-paths and, by this feature, Pyntacle drops the computational burden of elaborating big matrices/graphs. This happens regardless of whether one uses BF or GO search strategies. However, we agree that Pyntacle could greatly benefit from more sophisticated algorithms and the implementation of a range of different search and optimization algorithms (e.g. particle

swarm, ant colony, stochastic gradient descent, etc.), that the user can select from, is in our plans.

A.1.5. Details of parallelism and benchmarks.

From my understanding of the manuscript (and some experiments I tried with it detailed in Section B below), there are two types of parallelism used in Pyntacle. First, the "fine grained" parallelism it obtains simply from using the Numba library, and second, explicit "coarse grained" parallelism. The first type uses multithreading in lower level numeric libraries, similar to Microsoft R/Open using multithreaded linear algebra routines. The second type is used only for the brute-force algorithm however (and on Linux seems to work by forking new processes rather than using threads).
This reviewer is almost correct. Multi-threading is exploited by Numba in the calculation of all shortest-paths (Floyd-Warshall) and only for this task. We do not use any sort of off-the-shelf linear algebra library. Numba allows us to release the global interpreter lock when computing a (not so) restricted set of manipulation tasks on Numpy arrays. With the GIL released, we can spawn threads, handle concurrency, and, therefore, save a for-loop from the computation of Floyd-Warshall. Therefore, all metrics based on the calculation of the shortest-paths deal with this.
As a side note, in Pyntacle we have three codes to calculate all the shortest-paths of a graph:

- That borrowed from igraph (sequential)
- That implemented by us using Numba (multi-threading)
- That implemented by us using Numba (GPU)

The Brute-force search, on the other hand, relies on the *multiprocess* Python package and, as correctly noted, is used to fork processes and not threads. It is used exclusively here because Brute-force search knows the exact number of groups to be computed in advance. Although possible, we have discouraged the mixed-use of processes and threads.

It is not entirely clear, but it seems to me that Figure 4 shows results for using the greedy heuristic with a single thread (no parallelism). Thus it compares the same heuristic algorithm implemented in Pyntacle and R/keyplayer, showing that Pyntacle is faster (due to implementation details, being in Python not R, etc.).

This is a fair comparison if both implementations are really using only a single thread. However I found that Pyntacle used multithreading (up to the number of cores on the node it is run on) automatically, unless the NUMBA_NUM_THREADS environment variable is set (I am not familiar with the Numba library and had to find this in its online documentation). I could not find any documentation in the Pyntacle online manuals about parallelism or adjusting the number of threads, except for the -T/--threads option which applies to the brute-force algorithm only (not the greedy heuristic).

The keyplayer R package, however, uses a single thread unless explicitly requested to use parallelism by a function argument.

Hence I think this should be clarified in the manuscript, and some documentation of how parallelism works and how to adjust the number of threads added to the Pyntacle documentation.

Since the command-line interface of Pyntacle was designed for not-experts, we decided to hide one level of parallelism (the fine-grained) to the user and to automatically manage the parallelism related to the calculation of the shortest paths. We used this simple algorithm that we will make clever and adaptive in future releases.

```
// auto-select the computing mode
if nprocs > 1  // n.b., nprocs is user-defined
    Let's enable multi-process and disable multi-threading
else if size(graph) < 250 or rho(graph)<0.5  //rho measures sparseness
    Let's disable multi-process and disable multi-threading
else
    Let's disable multi-process and enable multi-threading
```

N.b., we have replaced -T/--threads with -O/--nprocs since it actually refers to the coarse-grained parallelism that forks processes and not threads.

The number of threads of the fine-grained parallelism is internally set to the *number of available cpus -1* by default, which can be tuned by setting the env variable NUMBA_NUM_THREADS, as correctly pointed out. However, caution must be paid on this: Numba adjusts the number of active threads *on-the-fly* according to the current overheads and, hence, the efficiency of parallelism. This means that what specified in the environment variable might not be actually respected. This is the reason why we were not explicit about this aspect. We did not want to cause confusion and frustrating experience to not-expert users.
We have made this clear in the online documentation.

Multi-threading is however controllable via APIs. To actually make fair comparisons, we benchmarked naked functions with code like this:

**parallelism_test.py**

```
from algorithms.bruteforce_search import BruteforceSearch
from io_stream.generator import PyntacleGenerator
from tools.enums import CmodeEnum, KpposEnum

if __name__ == '__main__':
```

```
graph_rnd = PyntacleGenerator.Random([100, 0.6])

start = time.perf_counter()

# Single-threaded
mreach_s = BruteforceSearch.reachability(
                graph_rnd,
                2,
                KpposEnum.mreach,
                None,
                m=2,
                cmode=CmodeEnum.igraph, #this enables single-threading
                nprocs=1)  #this is the default choice
end = time.perf_counter()
print("--- Elapsed time: {:.2f} seconds ---".format(end - start))
```

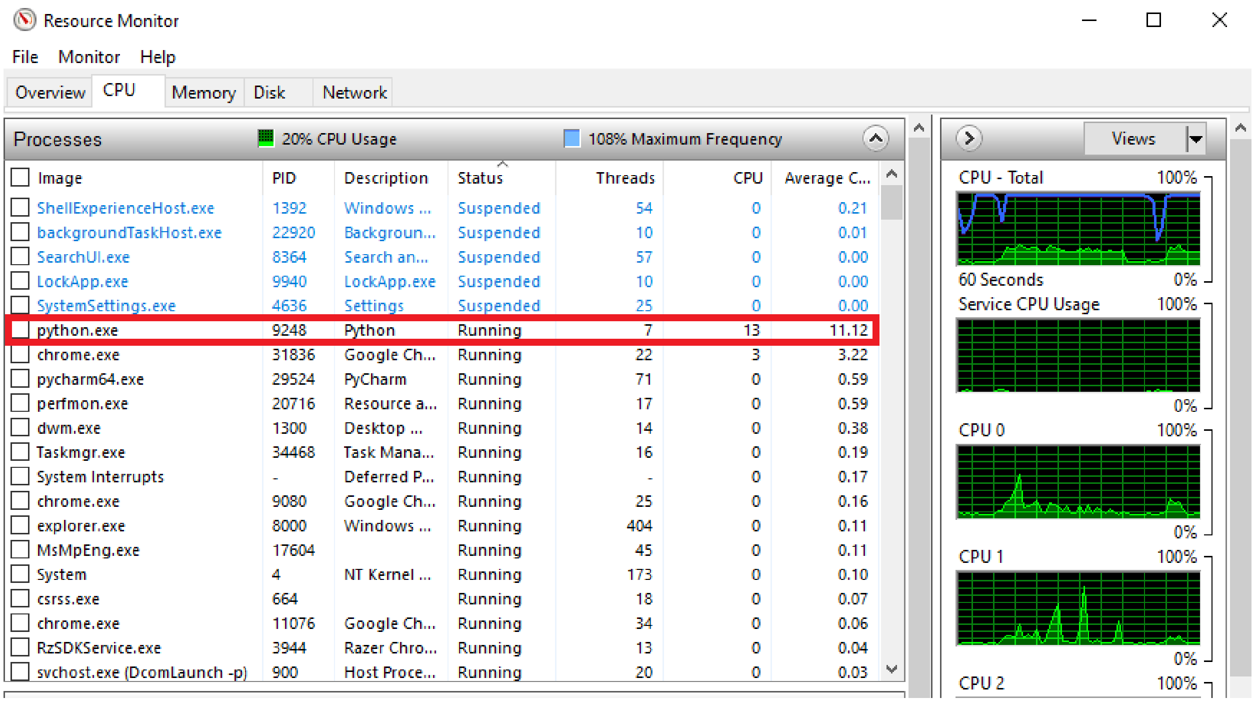


Multiprocessing (parallelism on sets) can be enabled via the option *nprocs=n* as follows:

```
...
BruteforceSearch.reachability(
                graph_rnd,
                2,
                KpposEnum.mreach,
                None,
```

```
                        m=2,
                        cmode=CmodeEnum.igraph,
                        nprocs=4)
...
```

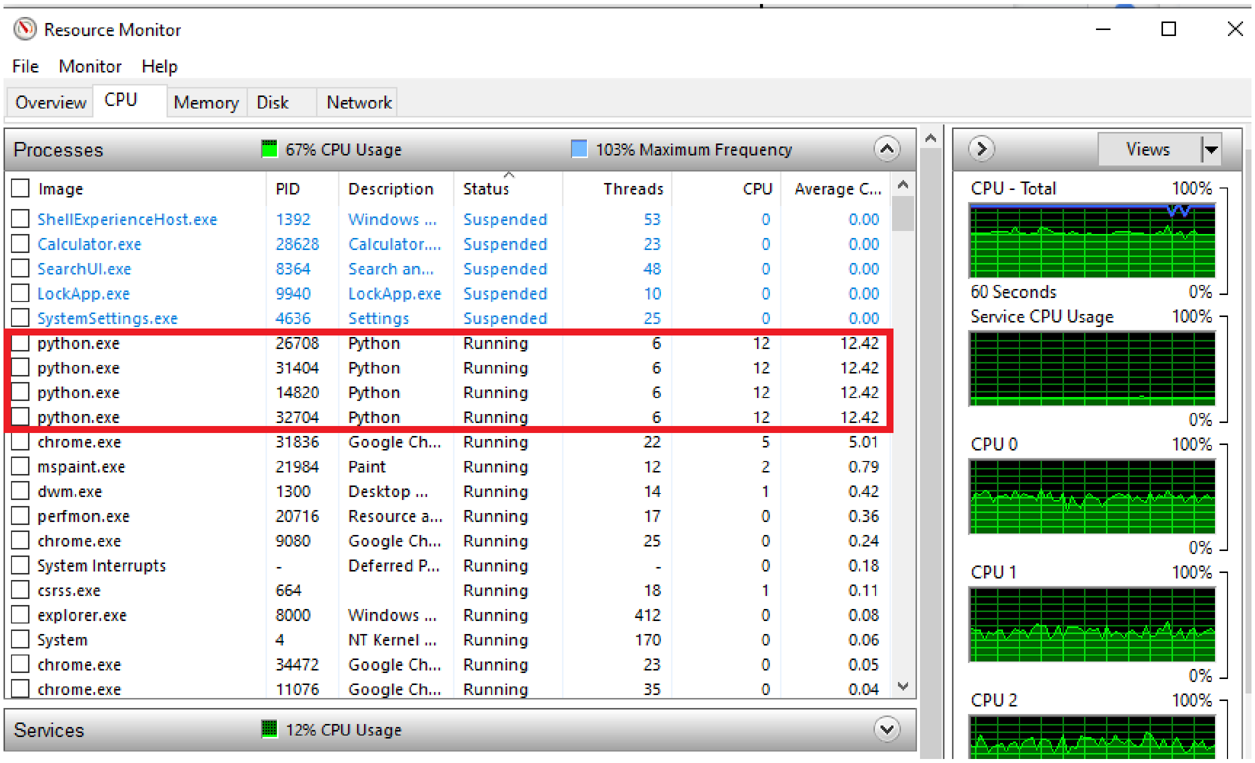


Mixing multi-processing with multi-threading is possible, although discouraged since it requires fine-tuning procs/threads ratios to perform well.

```
...
BruteforceSearch.reachability(
                        graph_rnd,
                        2,
                        KpposEnum.mreach,
                        None,
                        m=2,
                        cmode=CmodeEnum.cpu, #this enables multi-threading
                        nprocs=3)
...
```

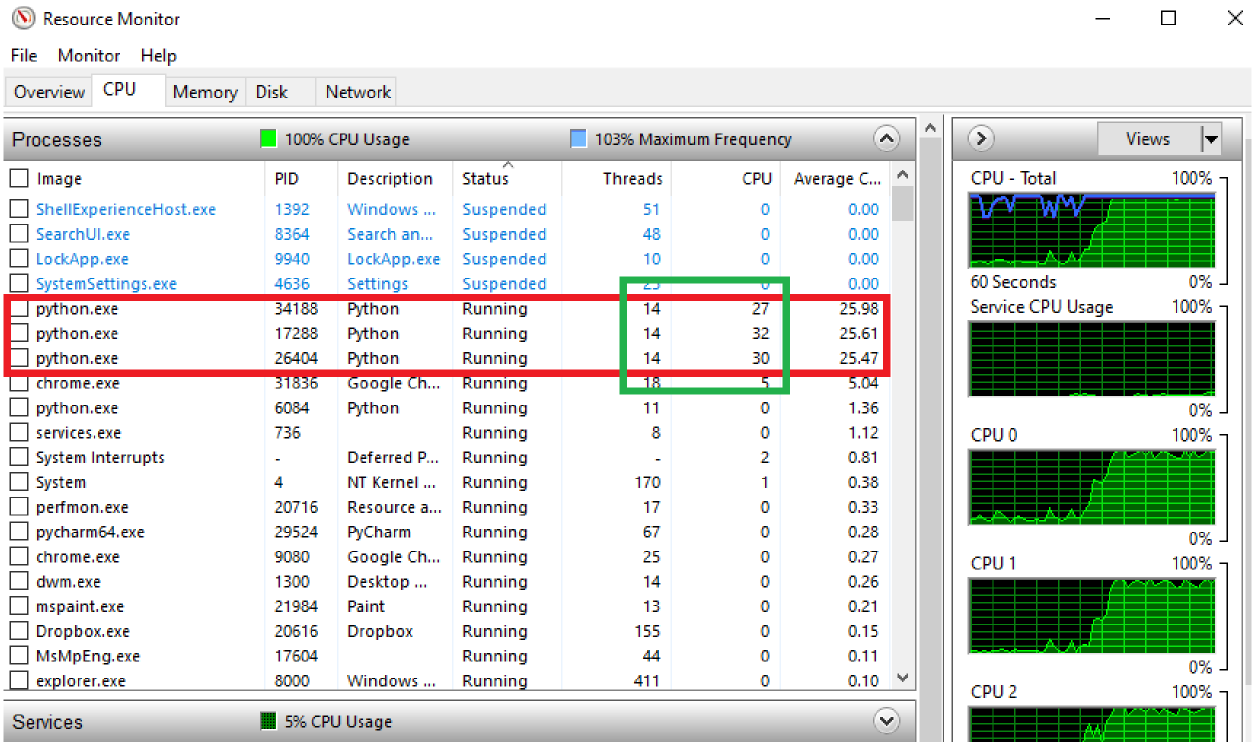


Fig. 5, however, shows the speedup obtained by Pyntacle using the brute-force algorithm with explicit (coarse-grained) parallelism as described, of computing the metric on the independent possible node sets in parallel. This is a valid comparison, however as per A.1.4 above, I question the practicality of the brute-force method at all, given the availability of many more efficient (albeit more complicated, and perhaps requiring a sophisticated ILP solver for example) algorithms for exact solution.

As in point A.1.4, we agree that there is a broad spectrum of better heuristic search algorithms and optimization techniques than the one currently implemented in Pyntacle. It is our goal to integrate them in the next releases of Pyntacle.
However, in GitHub, we have created a fork ("feat#31) where we have already implemented the Stochastic Gradient Descent algorithm. This branch is not still merged because this new implementation has to be carefully tested and unittests still need to be done.

A.1.6. Graphics processing unit (GPU).

The manuscript mentions graphics processing units (GPU) for parallelism, but I found no further details on this. When I run Pyntacle on a node with no GPU, I get a warning message that "NVIDIA: no NVIDIA devices found" which would seem to indicate that NVIDIA (CUDA) GPU support is available, and when I run the pyntacle keyplayer module on a cluster node which

does have a GPU, I do not get the warning message, but the GPU does not seem to be used (checking with the nvidia-smi program).

Note that I installed the 'miniconda' package on a cluster head node which does not have a GPU (details in Section B below).

This reviewer is correct. GPU-base processing is an experimental feature at the moment and, as noted by the algorithm above, not covered by the command line. This is because of weird behaviors of Numba with some hardware that might puzzle the user. The GPU feature will be stable in the release 2.0 when we will have covered the possibility to manage big matrices for which replacing fine-grained parallelism with GPU computing would make sense. We have clearly specified this in the documentation.
However, GPU-computing is currently possible by APIs:

```
...
BruteforceSearch.reachability(
                    graph_rnd,
                    2,
                    KpposEnum.mreach,
                    None,
                    m=2,
                    cmode=CmodeEnum.gpu,
                    nprocs=1)
...
```

This command applied on a 4000 nodes network makes Pyntacle use just ⅓ of the available GPU capabilities and a not negligible overhead. For this reason, also, we will fully include the GPU option when Pyntacle will be able to handle > 10.000 nodes, efficiently.

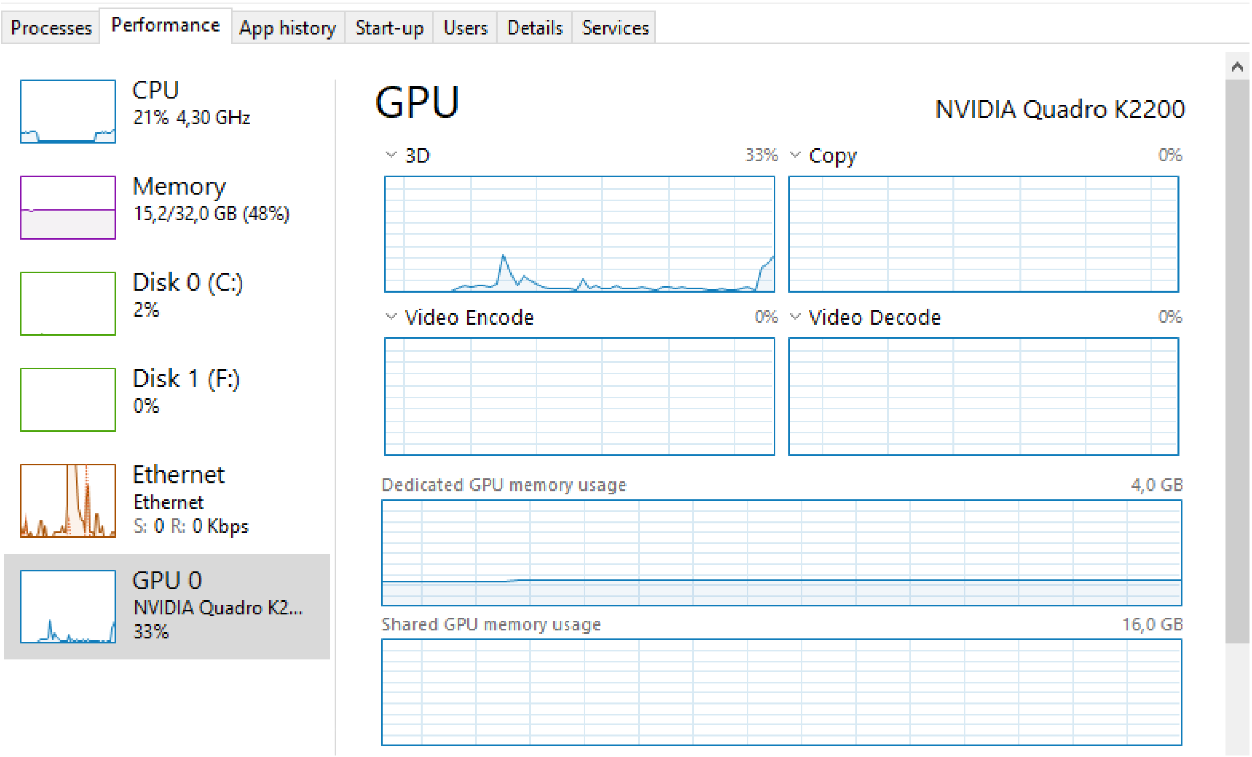


A.2. Minor issues

A.2.1. Some notational confusion with 'F'.

The variable F is first mentioned in Case Study 1 (p. 7 "... the best negative key-players in the network (according to both F and DF metrics), ...") but not defined, although it presumably refers to the fragmentation centrality of Borgatti (2006, equation (4)). However it is then used as a variable representing any centrality function (key-player metric) in Algorithms 1 and 2 (p. 9), which could be confusing.
Sorry for this. We have replaced F with FUNC in the algorithms. We have defined Borgatti's F metrics in methods. Thanks.

A.2.2. Some potential confusion for readers between KeyPlayer (Analytic Technologies) and R "keyplayer" package.

At the bottom of p. 5 (column 1): "... keyplayer is not rigorously testable here since it is a Windows-only GUI-based application." This could be confusing as the R keyplayer package (An & Liu, 2016) is previously styled as "keyplayer" (lowercase) while the Analytic Technologies KeyPlayer program (Borgatti, 2019) is styled as "KeyPlayer" ("camel case"), and it is the Analytic Technologies KeyPlayer program that is Windows-only.

Amended.

B. Notes on the Pyntacle software
---------------------------------

The Pyntacle website referenced in the manuscript ([http://pyntacle.css-mendel.it)]http://pyntacle.css-mendel.it) is easy to navigate and read, and contains extensive documentation on installation and usage, as well as, importantly, examples and case studies which can be followed and reproduced easily.

I installed the Pyntacle software on a Linux server (the master [head] node of the cluster described below, which has 2 x Intel E5-2630 v3, 16 (2 x 8) cores CPU,64GB DDR4 @ 2133MHz RAM) using the 'miniconda' system as recommended on the Pyntacle home page. This worked without problem, and I ran the tests which passed. I was also easily able to reproduce Case Study 3 (C. elegans connectome) linked from the software home page.

I then ran some of my own tests (using the command line interface, running as batch jobs on cluster compute nodes) to compare results between Pyntacle, the keyplayer (v1.0.3) R package (An & Liu, 2016) and (for KPP-Pos only) the influenceR R package (v0.l.0) (Jacobs et al., 2015).

I came across one bug in the command line interface: the --type m-reach option does not seem to work. But I found this already reported in the GitHub issue tracker as issue #23.

Amended

Note I ran the tests once only, so the times (which are elapsed times of the key player finding module only, as reported by Pyntacle itself, or system.time() for R packages) are merely indicative. For parallel tests I reserved all 20 cores on a compute node. For serial tests I reserved a single core on a compute node. I set environment variable NUMBA_NUM_THREADS for Pyntacle and OMP_NUM_THREADS for R packages using OpenMP, and the parallel flag and cluster option for the keyplayer R package, to the appropriate number of cores (20 or 1) to ensure the correct number of cores is (potentially) used.

All tests I conducted were on a Linux (CentOS 7.5 x86_64) cluster with 42 compute nodes and Intel 40Gbps QDR Infiniband. The compute nodes used have 2 x Intel Xeon E5-2650 v3 @ 2.30GHz, 20 (2 x 10) cores CPU, 64GB DDR4 @ 2133MHz RAM. The node with a GPU is 2 x Intel Xeon E5-2650 v3 @ 2.30GHz, 20 (2 x 10) cores CPU, 128GB DDR4 @ 2133MHz RAM, GPU:1 x NVIDIA GeForce GTX 1080 Founders Edition 8GB GDDR5X 2560 CUDA cores. The cluster scheduling system is slurm and the R version I used is Microsoft R Open v3.2.5.

Test 1: Key players DF (KPP-Neg) of size k = 2 in the C. elegans connectome (279 nodes), computed with greedy heuristic.

The network data set used is the CAEEL_Connectome.sif file as used in "Case Study 3" from the Pyntacle home page. I used the "pyntacle convert" command to convert it from SIF format to edgelist format for use in the R keyplayers package. Note that the influenceR package only does KPP-Pos so it is not included in this test.

| Method | DF value | Solutions | Elapsed time (s) |
|---|---|---|---|
| Pyntacle serial | 0.58991 | AVAL, AVAR | 18.73 |
| Pyntacle parallel | 0.58991 | AVAL, AVAR | 18.17 |
| R/keyplayer serial | 0.5899131 | AVAL, AVAR | 1940.055 |
| R/keyplayer parallel | 0.5899131 | AVAL, AVAR | 9576.714 |

So this shows Pyntacle computing the KPP-Neg (DF metric) correctly on this test, two orders of magnitude faster than the R keyplayer software. There is no real difference in the time for Pyntacle on 1 core versus 20 cores, perhaps unimportant when it takes less than 20 seconds anyway. I'm not sure why the keyplayer R package is so much slower with 20 cores than 1 core, but I suspect it is because the parallel mode in that package is for trying to avoid local minima by running the algorithm with different randomization seeds in parallel (An & Liu 2016, p. 264) rather than speeding the computation of a single run.

In running these tests I found a bug in Pyntacle-Ink (the HTML graphical output): for KPP-Neg metric dF it reports both "Initial Value" and "Value" but they appear to be swapped (they are the right way around in the stdout and the Report TSV file however).

Amended

Test 2: Key players DR (KPP-Pos) of size k = 2 in the C. elegans PPI network (3303 nodes), computed with greedy heuristic. (This should be the same test as the benchmark shown in Fig. 4 of the manuscript for (D)).

The network data used here is the APID_CAEEL_Level2_maincomponent.adjm adjacency matrix file as used in "Case Study 2" linked from the Pyntacle software home page, and referred to as "APID" in the manuscript.

| Method | DR value | Solutions | Elapsed time (s) |
|---|---|---|---|
| Pyntacle serial | 0.37486 | Q22174, Q95QA6 | 416.70 |
| Pyntacle parallel | 0.37486 | Q22174, Q95QA6 | 353.84 |

| | | | |
|---|---|---|---|
| influenceR serial | [not shown] | O45521, Q95QA6 | 1.208 |
| influenceR parallel | [not shown] | Q22174, Q95QA6 | 2.600 |
| R/keyplayer serial | [did not complete within 20 hour elapsed time limit] | | |
| R/keyplayer parallel | [did not complete within 20 hour elapsed time limit] | | |

So it seems to be working correctly, and on my test took about 400 seconds, compared to about 200 seconds in the manuscript Fig. 4, so this seems reasonable. And again, much faster than the keyplayer R package (which could not find a solution within 20 hours).

Again, however, using Pyntacle key player module using the greedy heuristic with 20 cores has only a relatively small advantage over a single core, even though the elapsed time is now several minutes. The Linux (tcsh) time builtin shows 99.8% CPU for the serial run and only 109.0% CPU for the parallel run, showing that little parallelism was used.

> the reason for that might be ascribed to the fact that Numba reduces parallelism when overheads become significant.

However, note that the influenceR R package, which uses a different heuristic algorithm, "stochastic gradient descent" (Jacobs et al., 2015, "keyplayer") is able to find the same solution as Pyntacle, but two orders of magnitude faster (note it also finds a different solution in the serial run - since it is a stochastic algorithm we could get different values each run - but unfortunately does not seem to report the objective value so we cannot see if it is a better or worse solution).

> this note is weak as pointed out by the reviewer him/herself, also because the SGD explores a smaller solution space than the GO algorithm implemented in Pyntacle. In particular:

| Stochastic Gradient Descent (SGD) | Greedy-optimization search (GO) |
|---|---|
| 1. Select k nodes at random to populate set S<br>2. Set F = fit using appropriate key player metric (KPP-Pos in our case)<br>3. Get a new state:<br>○ Pick a random u in S and v not in S.<br>○ F' = fit if u and v were swapped<br>○ If F' > F, swap u and v in S. Else, repeat step 3. (Alternatively, if a positive value is given for the `prob' parameter, a swap will be accepted with a small probability regardless of whether it improves the fit).<br><br>If F' - F < tolerance or our maximum computation time is exceeded, return S. Else, go to step 3. | 1. Select k nodes at random to populate set S<br>2. Set F = fit using appropriate key player metric<br>3. For each node *u* in S and each node *v* not in S<br>a. DELTAF=improvement in fit if *u* and *v* were swapped<br>4. Select pair with largest DELTAF<br>a. If DELTAF <= then terminate<br>b. Else, swap pair with greatest improvement in fit and set F=F+DELTAF<br>5. Go to step 3 |

| | |
|---|---|
| (from: https://www.rdocumentation.org/packages/influenceR/versions/0.1.0/topics/keyplayer) | |

> SGD adds a random contribution that (can) supersedes the fitting function and saves the loop in red in the GO algorithm. This last fact increases the chance to fall into disadvantageous local maxima, much more than the other algorithm. Even without any deep analysis, out of this scope, it is intuitive why the former is faster than the latter, as it is equally clear that the chances for the former to get the best solution are less than those of the GO algorithm.

Note also that influenceR (despite the short elapsed time) also seems to be making good use of multicore (20 cores): the (Linux) time command shows similar user and elapsed time for the serial run (as expected), but the parallel run shows 26.137 user CPU seconds and only 1.208 elapsed seconds (and only 0.016 system).

Note I also tried to find the key player set size k = 2 for the DF metric (KPP-Neg) with the greedy heuristic in Pyntacle in this data set, but it was unable to complete with the time limit of 20 hours (serial or parallel).

> Pyntacle will do better with more efficient search algorithms in the next releases.

C. Network software libraries/packages referenced
-----------------------------------------------

This list is not necessarily exhaustive, but are some packages that I have used or are aware of, particularly those that are usable from Python. They are all general-purpose graph libraries, with the exception of influenceR and keyplayer, which are specifically for the key players problem. Some have many more features than others though; for example SNAP (Leskovec) handles very large networks efficiently, but has very few algorithms implemented compared to igraph or NetworkX).

| Name | Website | Citation | Interface | Implementation | Parallel | KeyPlayers |
|---|---|---|---|---|---|---|
| graph-tool | https://graph-tool.skewed.de/ | Peixoto (2014) | Python | C++, Boost | OpenMP | No |
| igraph | https://igraph.org/ | Csardi & Nepusz (2006) | Python,R,C/C++,Mathematica | C | No | No |
| influenceR | https://cran.r-project.org/web/packages/influenceR/index.html | Jacobs et al. (2015) | R | R, igraph, SNAP(Bader) | OpenMP | KPP-Pos(DR) |

keplayer https://cran.r-project.org/web/packages/keyplayer/index.html An & Liu (2016) R R, igraph, sna R/parallel Yes
NetworkX http://networkx.github.io/ Hagberg et al. (2008) Python Python, SciPy No No
sna (statnet) https://cran.r-project.org/web/packages/sna/index.html Butts (2008) R C No No
SNAP (Bader) http://snap-graph.sourceforge.net/ Bader & Madduri (2008); Madduri & Bader (2009) C C OpenMP No
SNAP (Leskovec) http://snap.stanford.edu/ Leskovec & Sosič(2016) Python, C++, NodeXL C++ Maybe? No

D. References (in addition to those in manuscript)
--------------------------------------------------

Arulselvan, A., Commander, C. W., Elefteriadou, L., & Pardalos, P. M. (2009). Detecting critical nodes in sparse graphs. Computers & Operations Research, 36(7), 2193-2200.

Bader, D. A., & Madduri, K. (2008, April). Snap, small-world network analysis and partitioning: An open-source parallel graph framework for the exploration of large-scale networks. In 2008 IEEE international symposium on parallel and distributed processing (pp. 1-12). IEEE.

Boginski, V., & Commander, C. W. (2009). Identifying critical nodes in protein-protein interaction networks. In Clustering challenges in biological networks (pp. 153-167).

Butts, C. T. (2008). Social network analysis with sna. Journal of statistical software, 24(6), 1-51.

Celestini, A., Cianfriglia, M., Mastrostefano, E., Palma, A., Castiglione, F., & Tieri, P. (2019). Critical nodes reveal peculiar features of human essential genes and protein interactome. bioRxiv, 831750.

Csardi, G., & Nepusz, T. (2006). The igraph software package for complex network research. InterJournal, complex systems, 1695(5), 1-9.

Csermely, P., Korcsmáros, T., Kiss, H. J., London, G., & Nussinov, R. (2013). Structure and dynamics of molecular networks: a novel paradigm of drug discovery: a comprehensive review. Pharmacology & therapeutics, 138(3), 333-408.

Hagberg, A., Swart, P., & S Chult, D. (2008). Exploring network structure, dynamics, and function using NetworkX (No. LA-UR-08-05495; LA-UR-08-5495). Los Alamos National Lab.(LANL), Los Alamos, NM (United States).

Jacobs, S., Khanna, A., & Madduri, K. Bader D. influenceR: Software Tools to Quantify Structural Importance of Nodes in a Network. 2015. https://cran.r-project.org/package=influenceR [accessed 7 April 2020]. R package version 0.1.0.

Jiang, C., & Liu, Z. (2019). Detecting multiple key players under the positive effect by using a distance-based connectivity approach. Physica A: Statistical Mechanics and its Applications, 534, 122322.

Jiang, C., Liu, Z., Wang, J., Yu, H., & Guo, X. (2017). An optimal approach for the critical node problem using semidefinite programming. Physica A: Statistical Mechanics and its Applications, 471, 315-324.

Lalou, M., Tahraoui, M. A., & Kheddouci, H. (2018). The critical node detection problem in networks: A survey. Computer Science Review, 28, 92-117.

Leskovec, J., & Sosič, R. (2016). Snap: A general-purpose network analysis and graph-mining library. ACM Transactions on Intelligent Systems and Technology (TIST), 8(1), 1-20.

Madduri, K., & Bader, D. A. (2009, May). Compact graph representations and parallel connectivity algorithms for massive dynamic network analysis. In 2009 IEEE International Symposium on Parallel & Distributed Processing (pp. 1-11). IEEE.

McGuire, R. M., Deckro, R. F., & Ahner, D. K. (2015). The weighted key player problem for social network analysis. Military Operations Research, 20(2), 35-53.

Paudel, N., Georgiadis, L., & Italiano, G. F. (2018). Computing critical nodes in directed graphs. Journal of Experimental Algorithmics, 23(2):2.2.

Peixoto, T. (2014). The graph-tool python library. figshare. DOI: 10.6084/m9.figshare.1164194

Schrödinger, LLC. The PyMOL Molecular Graphics System, Version 2.3.3.

Tomaino, V., Arulselvan, A., Veltri, P., & Pardalos, P. M. (2012). Studying connectivity properties in human protein-protein interaction network in cancer pathway. In Data Mining for Biomarker Discovery (pp. 187-197). Springer, Boston, MA.

Ventresca, M. (2012). Global search algorithms using a combinatorial unranking-based problem representation for the critical node detection problem. Computers & Operations Research, 39(11), 2763-2775.

Walteros, J. L., & Pardalos, P. M. (2012). Selected topics in critical element detection. In Applications of mathematics and informatics in military science (pp. 9-26). Springer, New York, NY.

> Irrespective of the success of this submission, we would be happy to keep in touch with this reviewer to get deeper into performance issues and new developments and applications of Pyntacle in ours and other research fields. We are grateful for this precise and in depth review.

Reviewer #2: In this article, the authors describe Pyntacle, a Python-based software tool for the analysis of networks. Specifically, they focus on identifying groups of nodes by performing a combinatorial evaluation, of various parameters/indices/centrality measures. The manuscript provides a good outline of the package and its capabilities. Their approach is explained well; I found Figure 1 to be a good toy example to illustrate the capabilities of the network. I have a few concerns for the authors to comment upon/implement to further improve their manuscript.

Specific comments:
1. I find the networks in Figure 5 to be very small and not representative of biological networks in complexity, size and density. It would be nice if the examples are changed to 10^3 and 10^4 nodes, discarding 100 nodes. Or, will the combinatorial complexity become a major problem for larger networks?

As we have shown in the case study, with the network derived from a protein-protein interaction interface, the target of Pyntacle is represented also by small networks (<100 nodes/edges), therefore we wanted to present the different scenarios in which the potential user can find Pyntacle useful, e.g. residue contact or H-bond networks in a complex during a molecular dynamics simulation. Bigger networks (10^3 or 10^4 nodes) would not equally display the same level of informativeness in a Figure given their sizes; moreover, they would not represent a fair comparison ground with the other methods, since already it took more than one day of computation with networks in the 10^3 category, revealing the speedup gain when using Pyntacle. Finally, 10^4-node networks are indeed combinatorially complex to analyze, they will be properly dealt with in the next release of Pyntacle, with a stable GPU implementation and faster heuristic search algorithms and optimization approaches to choose from (as pointed out by reviewer 1 as well).

2. Perhaps, it would be nice if another example, of a protein-protein functional association network from the STRING database is considered. It would be a biologically motivating example, given the opening discussions about protein interaction networks.

The C.elegans network, collected from APID and used for benchmarking and tests in the manuscript, is built from quality-checked protein-protein interaction data. We clarified this in the text.

3. Could any heuristics be used to cut down the combinatorial search, especially for larger networks. For instance, could nodes with a degree of 1 be ignored? Can the authors suggest some heuristics to simplify computations for larger networks, based on their observations of what nodes typically figure in the "groups".

The greedy optimization heuristics is shown to identify near-optimal solutions in the manuscript. It represents a fast and simple implementation, even if it is only a starting point. As pointed out by reviewer 1, there is a plethora of faster and more efficient heuristic search algorithms that we aim to include in Pyntacle, so that the user can select different algorithms of choice. (N.b., as a first reaction to this review, we have started and almost finished to implement another search algorithm, the Stochastic Gradient Descent, as it is verifiable in the Pyntacle's GitHub website, branch "feat#31"). We think this is the right path to follow. We do not recommend node (network leaves) pruning as it would greatly influence the outcome of different metrics (for example, the m-reach parameter would yield different results since the removed nodes cannot be reached).

4. `pyntacle test` works very well. Will it be possible to have `pyntacle demo` similarly, which can showcase some of the capabilities? Also, can some example networks be included?

In order to gradually introduce the user to Pyntacle, we created a quick-start guide and 3 case studies, all detailed with data explanation, command lines and graphs both in the forms of HTML pages and python notebooks. We feel that a Pyntacle "demo" function would not be as welcoming to new users as the guides and case studies. Several examples can be downloaded directly from the quick-start guide/case studies in the website. Moreover, the adjacency matrices associated to the network analyzed in the two case studies can now be accessed from GigaDB.

5. An example case study with a larger network, such as E. coli from STRING would also add a lot of value to the paper.

The STRING network for C.elegans is one order of magnitude bigger than the E.coli network from the same resource: the STRING C.elegans network counts 18181 nodes (6170 when considering only high-quality interactions with a combined score equal or higher than 900) while the STRING E.coli K12 W3110/MG1655 networks count respectively 4210/4125 nodes (which decrease to 2698/3259 nodes when applying the same high-quality filter). The C.elegans

network we used (collected from the APID resource) counts 3303 nodes, same order of magnitude of the STRING networks, and therefore is a suitable representative of this kind of ppi networks. The number of edges greatly varies on the quality of each reported interaction.

Optional features:
1. Lastly, it would be nice if there is a way to pull networks from commonly used databases such as networkrepository.com or STRING. This would enable a lot of network scientists and biologists to easily perform various network analyses.

This is a great option to be added in the immediate next release of Pyntacle, the user would be given the choice of selecting among the most used repositories (e.g. STRING, IMEx and APID).

Typographical errors:
Page 1, Para 1: Protein interaction networks, ... represents -> represent
Page 1, Para 1: These, -> unclear antecedent -- could we re-written
Page 1, Para 3: "having relatively few"" -> "having a few relatively"

We corrected these errors. Thanks for this valuable review.
