# Supplementary figures and images for: Pyntacle: a parallel computing-enabled framework for large-scale network biology analysis
Source: Gigascience. 2020 Oct 21;9(10):giaa115. doi: 10.1093/gigascience/giaa115 (PMC7576925; doi:10.1093/gigascience/giaa115)

Color Key

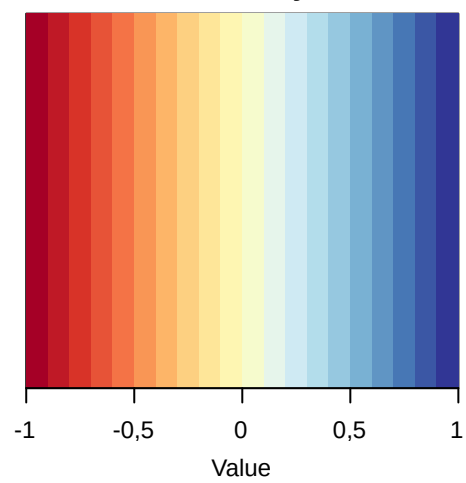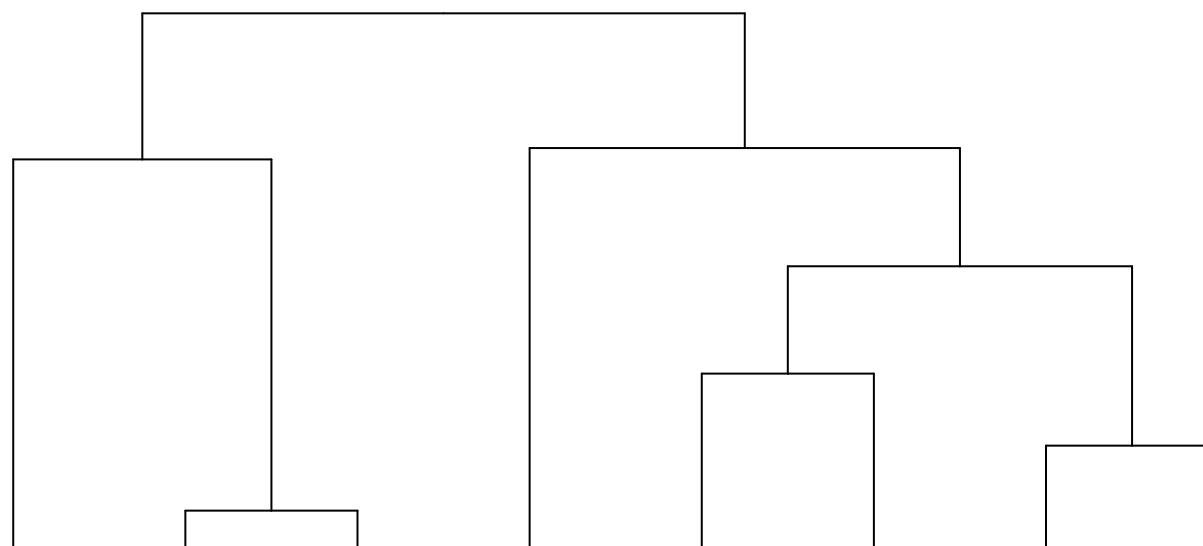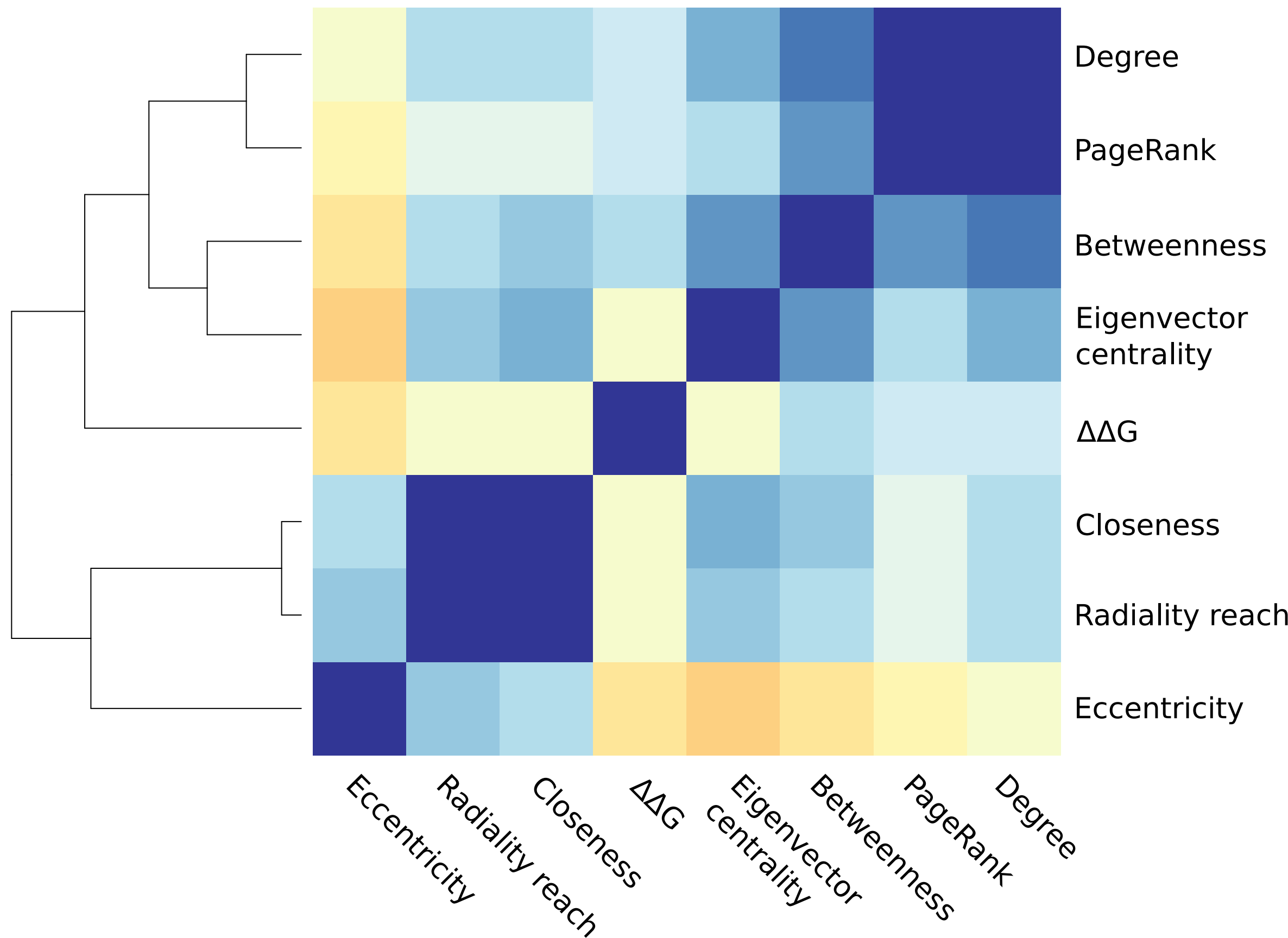

Supplement: giaa115_Supplemental_Files [file giaa115_supplemental_files.zip › Supplementary Figure S1.pdf]
