# Supplementary material for: Pyntacle: a parallel computing-enabled framework for large-scale network biology analysis
Source: Gigascience. 2020 Oct 21;9(10):giaa115. doi: 10.1093/gigascience/giaa115 (PMC7576925; doi:10.1093/gigascience/giaa115)
Supplement: giaa115_Response_to_Reviewer_Comments_Original_Submission [file giaa115_response_to_reviewer_comments_original_submission.pdf]

Author's Response To Reviewer Comments

Close

<< please note that following the cover letter, in the same PDF file, the editor and reviewers can find a better formatted rebuttal letter, with images attached, which can better support our answers>>

Reviewer #1: "Pyntacle: a parallel computing-enabled framework for large-scale network biology analysis" GigaScience manuscript (Technical Note) GIGA-D-20-00087

This manuscript describes a software library for graph computations usable as both a Python library and command line interface. In addition, it describes an interactive viewer output (Pyntacle-Ink) module for visualizing the results. Although Pyntacle is a general-purpose graph library, it has a particular focus (as does the manuscript) on group centrality metrics and the "key player problem" (KPP), which is the computationally intractable problem of finding sets of (rather than individual) nodes in a network which maximize (or minimize) a group centrality metric.

This distinguishes it from other general-purpose network packages, which usually do not include algorithms for the KPP, while those packages that do address it tend to be specific to that problem, and not general purpose libraries (see section C below).

Another distinguishing feature of Pyntacle is that it is targeted particularly at large-scale network biology analysis, and emphasizes parallel computing. Although the KPP is well-known in social network analysis (from which it originated), it is not so well-known in biology (although not unknown, see A.1.1 below) and so this manuscript makes a contribution in introducing it (and making available usable multi-platform software in a platform widely used in bioinformatics, i.e. Python) to the field.

A. Notes on the manuscript

The manuscript is generally clear, giving a (quite lengthy) introduction and exposition of the KPP, description of the software and algorithms implemented, benchmark results, and two case studies.
The software (both source code and installation packages), documentation, and data sets required for reproducibility are all referenced and freely available from the project website (although see A.1.3 below for an exception).

A.1. Major issues
These are points I think must be addressed before the manuscript can be accepted.

A.1.1. Insufficient references to existing research literature.
The KPP originated in social network research with the "key player problem" so named by Borgatti (2006). However in other fields, and specifically computer science and applied mathematics (operations research for example), the KPP and variants are known as "critical element detection" or the "critical node problem" (CNP) (Walteros & Pardalos, 2012), or the "critical node detection problem" (CNDP), of which the CNP is a variant (Lalou et al. (2018)).

This is an active research field, as evidenced by the review paper Lalou et al. (2018), and although a review of this field is clearly outside the scope of this manuscript (technical note), at least a brief mention of the relationship of the KPP to the CNDP, and a citation of (for example) the Lalou et al. (2018) review paper would be of value to readers and not leave them with the misleading impression that the KPP has only been studied in a couple of papers and only the brute-force and greedy heuristic algorithms have been studied to solve it.

As well as this general issue, I think some references to the prior use of the KPP (or related CNDP variants) in biology would be welcome, for example in protein-protein interaction (PPI) networks (Boginsnki & Commander, 2009; Tomaino et al., 2012), and genetic regulatory networks (Celestini et al., 2019). For a more general perspective on network methods in drug discovery, the extensive review

of Csermely et al. (2013) could be informative.

In the concluding remarks (p. 8) where extension to weighted and directed networks is mentioned, it might be noted that some work exists on the CNDP in directed graphs (Paudel et al., 2018) and KPP on weighted graphs (McGuire et al., 2015; Yang, 2015; Jiang & Liu, 2019). Indeed the R keyplayer package is claimed to work with both directed and weighted networks (An & Liu, 2016).

Although reproducing results from the many algorithms for solving the KPP or CNDP variants (either exactly i.e. to optimality, or to potentially suboptimal solutions with heuristics) is clearly impractical, rough comparisons to other methods could be facilitated by running the benchmarks on the Critical Node Detection Problem Benchmark Instances (Ventresca 2012), available from http://individual.utoronto.ca/mventresca/cnd.html, which are used in many CNDP papers.

> We thank the reviewer for the suggested reads, we integrated them in the revised manuscript. As far as the proposed benchmark is concerned, we think that it would be very informative and useful. However, we prefer to postpone this to the next future for two reasons:
1- as the reviewer has noticed, Pyntacle is equipped with just one heuristic method. We think that the suggested effort would be much more effective to take when Pyntacle will run several more algorithms. This will happen with the next major release, scheduled to come out sometimes in the second half of the next year.
2- we are aware that those presented are preliminary benchmarks and we were struggling with whether to present them or not. We would prefer to keep them at this preliminary level or, eventually, completely removing them, if suggested by the reviewers.

A.1.2. Insufficient references to existing software.
Although a review of existing network libraries is beyond the scope of the manuscript, it could be useful for readers to at least mention some other general purpose graph libraries usable from Python such as graph-tool (Peixoto, 2014), igraph (Csardi & Nepusz, 2006), NetworkX (Hagberg et al., 2008), and SNAP (Leskovec & Sosič, 2016). (See Section C below for more).

Most particularly, it is noted that Pyntacle uses igraph modules, yet the igraph paper (Csardi & Nepusz, 2006) is not cited.

In addition, although the Analytic Technologies KeyPlayer program and the R keyplayer package (An & Liu, 2016) are mentioned, the influenceR R package (Jacobs et al., 2015) is not mentioned. Although this package only solves KPP-Pos with the DF metric, as I show in Section B below (on a single example) it does so much faster than Pyntacle.

> We integrated the references in the revised manuscript.

A.1.3. Reproducibility (data availability) of case studies.

For Case Study 1, I was easily able to reproduce the 3D protein cartoon in Fig. 6 by downloading the 5XTD structure from the RCSB PDB into PyMOL (Schrödinger, LLC), selecting the appropriate two chains, and locating the key player residues as shown. However to obtain the network of "The 138 residues forming the interaction interface" I would (according to the Methods) have to conduct several operations in FoldX and then extract the network according to a distance threshold described in the Methods. Although it seems there is sufficient detail to reproduce this, it requires a registration to use FoldX and some further work construct the network.

I think it would assist reproducibility (an important consideration in the GigaScience journal) to supply the network as constructed by the authors as a Supplementary Information file (as for example the results of the Pyntacle analysis are already supplied in an Excel file).

Similarly, the network constructed for Case Study 2 could also be made available as Supplementary Information.

> We have stored the adjacency matrices of the two case studies in GigaDB. N.b., A member of the GigaScience editorial team confirmed that a link to these files will be made available eventually after the publication of this piece of research. Moreover, we have explained how we made them in order to improve replicability.
In fact, in Case Study 1, the 138 residues identified via Foldx are those considered at the interface and

for which energetic contributions were calculated. The network we built has been obtained by directly analyzing the PDB file of the complex, connecting residues of different chains, and relying only on distance constraints (therefore avoiding external software like FoldX). As we demonstrate in the case study, valuable information is gained nonetheless, without using external software (FoldX) and in much less time.

A.1.4. Use of "brute-force" algorithm and "large-scale".

The manuscript is described (including in the title) as being for "large-scale" network analysis, and parallelism is emphasized. However (related to point A.1.1 above) it seems to me that the emphasis is perhaps misplaced. To be specific, two algorithms are implemented and benchmarked: the "brute-force" algorithm, and the greedy heuristic algorithm of Borgatti (2006). Parallelism then mainly seems to be discussed in relation to the brute-force algorithm.

However, as the KPP is known to be computationally intractable and indeed NP-complete (Arulselvan et al., 2009), a brute-force algorithm is never going to be practical, and certainly not for large-scale instances. For this reason there are two practical approaches: (a) exact solution by more sophisticated methods (for example integer linear programming (ILP), semidefinite programming (Jiang et al., 2017), branch-and-cut methods, etc.), and (b) heuristic algorithms to find a potentially suboptimal solution, but much faster. Note that the latter are also vital as input into the former (as bounds on the solution). For a review of these approaches see Lalou et al. (2018).

Hence my concern is that implementing and applying parallel computation to the brute-force approach is misplaced, as it can never be competitive with sophisticated exact solution algorithms (particularly where these could also be parallelized) on large-scale problems.

Regarding the heuristic (greedy) algorithm, it also seems that better results could be obtained with a more sophisticated heuristic, as already foreshadowed by Borgatti (2006). For example I demonstrate in Section B below, that the influenceR program is two orders of magnitude faster (albeit on a single example) than Pyntacle for KPP-Pos, as it uses a different heuristic algorithm. In addition there are many publications showing superior results with different heuristic algorithms for CNDP variants (see Lalou et al., 2018 for a review).

> BF was implemented because it is exact and without aiming to compete with any other search algorithm. The fact that we implemented some parallelism for it is because some fields of research, like ecology, deal with small/medium size networks, where size is in the order of a few tens/hundreds of nodes. Why should we yield suboptimal results for these when exact is achievable?
We were not clear enough on this: parallelism was applied to BF (coarse+fine grain) as well as on heuristics (fine grain only). Fine-grain parallelism applies directly to the way we enumerate shortest-paths and, by this feature, Pyntacle drops the computational burden of elaborating big matrices/graphs. This happens regardless of whether one uses BF or GO search strategies. However, we agree that Pyntacle could greatly benefit from more sophisticated algorithms and the implementation of a range of different search and optimization algorithms (e.g. particle swarm, ant colony, stochastic gradient descent, etc.), that the user can select from, is in our plans.

A.1.5. Details of parallelism and benchmarks.

From my understanding of the manuscript (and some experiments I tried with it detailed in Section B below), there are two types of parallelism used in Pyntacle. First, the "fine grained" parallelism it obtains simply from using the Numba library, and second, explicit "coarse grained" parallelism. The first type uses multithreading in lower level numeric libraries, similar to Microsoft R/Open using multithreaded linear algebra routines. The second type is used only for the brute-force algorithm however (and on Linux seems to work by forking new processes rather than using threads).

> This reviewer is almost correct. Multi-threading is exploited by Numba in the calculation of all shortest-paths (Floyd-Warshall) and only for this task. We do not use any sort of off-the-shelf linear algebra library. Numba allows us to release the global interpreter lock when computing a (not so) restricted set of manipulation tasks on Numpy arrays. With the GIL released, we can spawn threads, handle concurrency, and, therefore, save a for-loop from the computation of Floyd-Warshall. Therefore, all metrics based on the calculation of the shortest-paths deal with this.
As a side note, in Pyntacle we have three codes to calculate all the shortest-paths of a graph:
- That borrowed from igraph (sequential)

- That implemented by us using Numba (multi-threading)
- That implemented by us using Numba (GPU)

The Brute-force search, on the other hand, relies on the multiprocess Python package and, as correctly noted, is used to fork processes and not threads. It is used exclusively here because Brute-force search knows the exact number of groups to be computed in advance. Although possible, we have discouraged the mixed-use of processes and threads.

It is not entirely clear, but it seems to me that Figure 4 shows results for using the greedy heuristic with a single thread (no parallelism). Thus it compares the same heuristic algorithm implemented in Pyntacle and R/keyplayer, showing that Pyntacle is faster (due to implementation details, being in Python not R, etc.).

This is a fair comparison if both implementations are really using only a single thread. However I found that Pyntacle used multithreading (up to the number of cores on the node it is run on) automatically, unless the NUMBA_NUM_THREADS environment variable is set (I am not familiar with the Numba library and had to find this in its online documentation). I could not find any documentation in the Pyntacle online manuals about parallelism or adjusting the number of threads, except for the -T/--threads option which applies to the brute-force algorithm only (not the greedy heuristic).
The keyplayer R package, however, uses a single thread unless explicitly requested to use parallelism by a function argument.

Hence I think this should be clarified in the manuscript, and some documentation of how parallelism works and how to adjust the number of threads added to the Pyntacle documentation.

> Since the command-line interface of Pyntacle was designed for not-experts, we decided to hide one level of parallelism (the fine-grained) to the user and to automatically manage the parallelism related to the calculation of the shortest paths. We used this simple algorithm that we will make clever and adaptive in future releases.

```
// auto-select the computing mode
if nprocs > 1 // n.b., nprocs is user-defined
Let’s enable multi-process and disable multi-threading
else if size(graph) < 250 or rho(graph)<0.5 //rho measures sparseness
Let’s disable multi-process and disable multi-threading
else
Let’s disable multi-process and enable multi-threading
```

N.b., we have replaced -T/--threads with -O/--nprocs since it actually refers to the coarse-grained parallelism that forks processes and not threads.

The number of threads of the fine-grained parallelism is internally set to the number of available cpus -1 by default, which can be tuned by setting the env variable NUMBA_NUM_THREADS, as correctly pointed out. However, caution must be paid on this: Numba adjusts the number of active threads on-the-fly according to the current overheads and, hence, the efficiency of parallelism. This means that what specified in the environment variable might not be actually respected. This is the reason why we were not explicit about this aspect. We did not want to cause confusion and frustrating experience to not-expert users.
We have made this clear in the online documentation.

Multi-threading is however controllable via APIs. To actually make fair comparisons, we benchmarked naked functions with code like this:

```
parallelism_test.py
from algorithms.bruteforce_search import BruteforceSearch
from io_stream.generator import PyntacleGenerator
from tools.enums import CmodeEnum, KpposEnum

if __name__ == '__main__':
graph_rnd = PyntacleGenerator.Random([100, 0.6])

start = time.perf_counter()
```

```
# Single-threaded
mreach_s = BruteforceSearch.reachability(
graph_rnd,
2,
KpposEnum.mreach,
None,
m=2,
cmode=CmodeEnum.igraph, #this enables single-threading
nprocs=1) #this is the default choice
end = time.perf_counter()
print("--- Elapsed time: {:.2f} seconds ---".format(end - start))
```

Multiprocessing (parallelism on sets) can be enabled via the option nprocs=n as follows:

```
...
BruteforceSearch.reachability(
graph_rnd,
2,
KpposEnum.mreach,
None,
m=2,
cmode=CmodeEnum.igraph,
nprocs=4)
...
```

Mixing multi-processing with multi-threading is possible, although discouraged since it requires fine-tuning procs/threads ratios to perform well.

```
...
BruteforceSearch.reachability(
graph_rnd,
2,
KpposEnum.mreach,
None,
m=2,
cmode=CmodeEnum.cpu, #this enables multi-threading
nprocs=3)
...
```

Fig. 5, however, shows the speedup obtained by Pyntacle using the brute-force algorithm with explicit (coarse-grained) parallelism as described, of computing the metric on the independent possible node sets in parallel. This is a valid comparison, however as per A.1.4 above, I question the practicality of the brute-force method at all, given the availability of many more efficient (albeit more complicated, and perhaps requiring a sophisticated ILP solver for example) algorithms for exact solution.

> As in point A.1.4, we agree that there is a broad spectrum of better heuristic search algorithms and optimization techniques than the one currently implemented in Pyntacle. It is our goal to integrate them in the next releases of Pyntacle.
However, in GitHub, we have created a fork ("feat#31) where we have already implemented the Stochastic Gradient Descent algorithm. This branch is not still merged because this new implementation has to be carefully tested and unittests still need to be done.

A.1.6. Graphics processing unit (GPU).

The manuscript mentions graphics processing units (GPU) for parallelism, but I found no further details

on this. When I run Pyntacle on a node with no GPU, I get a warning message that "NVIDIA: no NVIDIA devices found" which would seem to indicate that NVIDIA (CUDA) GPU support is available, and when I run the pyntacle keyplayer module on a cluster node which does have a GPU, I do not get the warning message, but the GPU does not seem to be used (checking with the nvidia-smi program).

Note that I installed the 'miniconda' package on a cluster head node which does not have a GPU (details in Section B below).

> This reviewer is correct. GPU-base processing is an experimental feature at the moment and, as noted by the algorithm above, not covered by the command line. This is because of weird behaviors of Numba with some hardware that might puzzle the user. The GPU feature will be stable in the release 2.0 when we will have covered the possibility to manage big matrices for which replacing fine-grained parallelism with GPU computing would make sense. We have clearly specified this in the documentation.
However, GPU-computing is currently possible by APIs:

```
...
BruteforceSearch.reachability(
graph_rnd,
2,
KpposEnum.mreach,
None,
m=2,
cmode=CmodeEnum.gpu,
nprocs=1)
...
```

This command applied on a 4000 nodes network makes Pyntacle use just ⅓ of the available GPU capabilities and a not negligible overhead. For this reason, also, we will fully include the GPU option when Pyntacle will be able to handle > 10.000 nodes, efficiently.

A.2. Minor issues

A.2.1. Some notational confusion with 'F'.

The variable F is first mentioned in Case Study 1 (p. 7 "... the best negative key-players in the network (according to both F and DF metrics), ...") but not defined, although it presumably refers to the fragmentation centrality of Borgatti (2006, equation (4)). However it is then used as a variable representing any centrality function (key-player metric) in Algorithms 1 and 2 (p. 9), which could be confusing.
Sorry for this. We have replaced F with FUNC in the algorithms. We have defined Borgatti's F metrics in methods. Thanks.

A.2.2. Some potential confusion for readers between KeyPlayer (Analytic Technologies) and R "keyplayer" package.

At the bottom of p. 5 (column 1): "... keyplayer is not rigorously testable here since it is a Windows-only GUI-based application." This could be confusing as the R keyplayer package (An & Liu, 2016) is previously styled as "keyplayer" (lowercase) while the Analytic Technologies KeyPlayer program (Borgatti, 2019) is styled as "KeyPlayer" ("camel case"), and it is the Analytic Technologies KeyPlayer program that is Windows-only.

> Amended.

B. Notes on the Pyntacle software

The Pyntacle website referenced in the manuscript ([http://pyntacle.css-mendel.it)]http://pyntacle.css-mendel.it) is easy to navigate and read, and contains extensive documentation on installation and usage, as well as, importantly, examples and case studies which can be followed and reproduced easily.

I installed the Pyntacle software on a Linux server (the master [head] node of the cluster described below, which has 2 x Intel E5-2630 v3, 16 (2 x 8) cores CPU,64GB DDR4 @ 2133MHz RAM) using the 'miniconda' system as recommended on the Pyntacle home page. This worked without problem, and I ran the tests which passed. I was also easily able to reproduce Case Study 3 (C. elegans connectome) linked from the software home page.

I then ran some of my own tests (using the command line interface, running as batch jobs on cluster compute nodes) to compare results between Pyntacle, the keyplayer (v1.0.3) R package (An & Liu, 2016) and (for KPP-Pos only) the influenceR R package (v0.l.0) (Jacobs et al., 2015).

I came across one bug in the command line interface: the --type m-reach option does not seem to work. But I found this already reported in the GitHub issue tracker as issue #23.

> Amended

Note I ran the tests once only, so the times (which are elapsed times of the key player finding module only, as reported by Pyntacle itself, or system.time() for R packages) are merely indicative. For parallel tests I reserved all 20 cores on a compute node. For serial tests I reserved a single core on a compute node. I set environment variable NUMBA_NUM_THREADS for Pyntacle and OMP_NUM_THREADS for R packages using OpenMP, and the parallel flag and cluster option for the keyplayer R package, to the appropriate number of cores (20 or 1) to ensure the correct number of cores is (potentially) used.

All tests I conducted were on a Linux (CentOS 7.5 x86_64) cluster with 42 compute nodes and Intel 40Gbps QDR Infiniband. The compute nodes used have 2 x Intel Xeon E5-2650 v3 @ 2.30GHz, 20 (2 x 10) cores CPU, 64GB DDR4 @ 2133MHz RAM. The node with a GPU is 2 x Intel Xeon E5-2650 v3 @ 2.30GHz, 20 (2 x 10) cores CPU, 128GB DDR4 @ 2133MHz RAM, GPU:1 x NVIDIA GeForce GTX 1080 Founders Edition 8GB GDDR5X 2560 CUDA cores. The cluster scheduling system is slurm and the R version I used is Microsoft R Open v3.2.5.

Test 1: Key players DF (KPP-Neg) of size k = 2 in the C. elegans connectome (279 nodes), computed with greedy heuristic.

The network data set used is the CAEEL_Connectome.sif file as used in "Case Study 3" from the Pyntacle home page. I used the "pyntacle convert" command to convert it from SIF format to edgelist format for use in the R keyplayers package. Note that the influenceR package only does KPP-Pos so it is not included in this test.

Method DF value Solutions Elapsed time (s)
Pyntacle serial 0.58991 AVAL, AVAR 18.73
Pyntacle parallel 0.58991 AVAL, AVAR 18.17
R/keyplayer serial 0.5899131 AVAL, AVAR 1940.055
R/keyplayer parallel 0.5899131 AVAL, AVAR 9576.714

So this shows Pyntacle computing the KPP-Neg (DF metric) correctly on this test, two orders of magnitude faster than the R keyplayer software. There is no real difference in the time for Pyntacle on 1 core versus 20 cores, perhaps unimportant when it takes less than 20 seconds anyway. I'm not sure why the keyplayer R package is so much slower with 20 cores than 1 core, but I suspect it is because the parallel mode in that package is for trying to avoid local minima by running the algorithm with different randomization seeds in parallel (An & Liu 2016, p. 264) rather than speeding the computation of a single run.

In running these tests I found a bug in Pyntacle-Ink (the HTML graphical output): for KPP-Neg metric dF it reports both "Initial Value" and "Value" but they appear to be swapped (they are the right way around in the stdout and the Report TSV file however).

> Amended

Test 2: Key players DR (KPP-Pos) of size k = 2 in the C. elegans PPI network (3303 nodes), computed with greedy heuristic. (This should be the same test as the benchmark shown in Fig. 4 of the manuscript for (D)).

The network data used here is the APID_CAEEL_Level2_maincomponent.adjm adjacency matrix file as used in "Case Study 2" linked from the Pyntacle software home page, and referred to as "APID" in the manuscript.

Method DR value Solutions Elapsed time (s)
Pyntacle serial 0.37486 Q22174, Q95QA6 416.70
Pyntacle parallel 0.37486 Q22174, Q95QA6 353.84
influenceR serial [not shown] O45521, Q95QA6 1.208
influenceR parallel [not shown] Q22174, Q95QA6 2.600
R/keyplayer serial [did not complete within 20 hour elapsed time limit]
R/keyplayer parallel [did not complete within 20 hour elapsed time limit]

So it seems to be working correctly, and on my test took about 400 seconds, compared to about 200 seconds in the manuscript Fig. 4, so this seems reasonable. And again, much faster than the keyplayer R package (which could not find a solution within 20 hours).

Again, however, using Pyntacle key player module using the greedy heuristic with 20 cores has only a relatively small advantage over a single core, even though the elapsed time is now several minutes. The Linux (tcsh) time builtin shows 99.8% CPU for the serial run and only 109.0% CPU for the parallel run, showing that little parallelism was used.

> the reason for that might be ascribed to the fact that Numba reduces parallelism when overheads become significant.

However, note that the influenceR R package, which uses a different heuristic algorithm, "stochastic gradient descent" (Jacobs et al., 2015, "keyplayer") is able to find the same solution as Pyntacle, but two orders of magnitude faster (note it also finds a different solution in the serial run - since it is a stochastic algorithm we could get different values each run - but unfortunately does not seem to report the objective value so we cannot see if it is a better or worse solution).

> this note is weak as pointed out by the reviewer him/herself, also because the SGD explores a smaller solution space than the GO algorithm implemented in Pyntacle. In particular:

Stochastic Gradient Descent (SGD) Greedy-optimization search (GO)
1. Select k nodes at random to populate set S
2. Set F = fit using appropriate key player metric (KPP-Pos in our case)
3. Get a new state:
○ Pick a random u in S and v not in S.
○ F' = fit if u and v were swapped
○ If F' > F, swap u and v in S. Else, repeat step 3. (Alternatively, if a positive value is given for the `prob' parameter, a swap will be accepted with a small probability regardless of whether it improves the fit).
If F' - F < tolerance or our maximum computation time is exceeded, return S. Else, go to step 3.

(from: https://www.rdocumentation.org/packages/influenceR/versions/0.1.0/topics/keyplayer)
1. Select k nodes at random to populate set S
2. Set F = fit using appropriate key player metric
3. For each node u in S and each node v not in S <<<<< Loop in red
a. DELTAF=improvement in fit if u and v were swapped
4. Select pair with largest DELTAF
a. If DELTAF <= then terminate
b. Else, swap pair with greatest improvement in fit and set F=F+DELTAF
5. Go to step 3

> SGD adds a random contribution that (can) supersedes the fitting function and saves the loop in red in the GO algorithm. This last fact increases the chance to fall into disadvantageous local maxima, much more than the other algorithm. Even without any deep analysis, out of this scope, it is intuitive why the former is faster than the latter, as it is equally clear that the chances for the former to get the best solution are less than those of the GO algorithm.

Note also that influenceR (despite the short elapsed time) also seems to be making good use of

multicore (20 cores): the (Linux) time command shows similar user and elapsed time for the serial run (as expected), but the parallel run shows 26.137 user CPU seconds and only 1.208 elapsed seconds (and only 0.016 system).

Note I also tried to find the key player set size k = 2 for the DF metric (KPP-Neg) with the greedy heuristic in Pyntacle in this data set, but it was unable to complete with the time limit of 20 hours (serial or parallel).

> Pyntacle will do better with more efficient search algorithms in the next releases.

C. Network software libraries/packages referenced

This list is not necessarily exhaustive, but are some packages that I have used or are aware of, particularly those that are usable from Python. They are all general-purpose graph libraries, with the exception of influenceR and keyplayer, which are specifically for the key players problem. Some have many more features than others though; for example SNAP (Leskovec) handles very large networks efficiently, but has very few algorithms implemented compared to igraph or NetworkX).

Name Website Citation Interface Implementation Parallel KeyPlayers
graph-tool https://graph-tool.skewed.de/ Peixoto (2014) Python C++, Boost OpenMP No
igraph https://igraph.org/ Csardi & Nepusz (2006) Python,R,C/C++,Mathematica C No No
influenceR https://cran.r-project.org/web/packages/influenceR/index.html Jacobs et al. (2015) R R, igraph, SNAP(Bader) OpenMP KPP-Pos(DR)
keyplayer https://cran.r-project.org/web/packages/keyplayer/index.html An & Liu (2016) R R, igraph, sna R/parallel Yes
NetworkX http://networkx.github.io/ Hagberg et al. (2008) Python Python, SciPy No No
sna (statnet) https://cran.r-project.org/web/packages/sna/index.html Butts (2008) R C No No
SNAP (Bader) http://snap-graph.sourceforge.net/ Bader & Madduri (2008); Madduri & Bader (2009) C C OpenMP No
SNAP (Leskovec) http://snap.stanford.edu/ Leskovec & Sosič(2016) Python, C++, NodeXL C++ Maybe? No

D. References (in addition to those in manuscript)

Arulselvan, A., Commander, C. W., Elefteriadou, L., & Pardalos, P. M. (2009). Detecting critical nodes in sparse graphs. Computers & Operations Research, 36(7), 2193-2200.
Bader, D. A., & Madduri, K. (2008, April). Snap, small-world network analysis and partitioning: An open-source parallel graph framework for the exploration of large-scale networks. In 2008 IEEE international symposium on parallel and distributed processing (pp. 1-12). IEEE.
Boginski, V., & Commander, C. W. (2009). Identifying critical nodes in protein-protein interaction networks. In Clustering challenges in biological networks (pp. 153-167).
Butts, C. T. (2008). Social network analysis with sna. Journal of statistical software, 24(6), 1-51.
Celestini, A., Cianfriglia, M., Mastrostefano, E., Palma, A., Castiglione, F., & Tieri, P. (2019). Critical nodes reveal peculiar features of human essential genes and protein interactome. bioRxiv, 831750.
Csardi, G., & Nepusz, T. (2006). The igraph software package for complex network research. InterJournal, complex systems, 1695(5), 1-9.
Csermely, P., Korcsmáros, T., Kiss, H. J., London, G., & Nussinov, R. (2013). Structure and dynamics of molecular networks: a novel paradigm of drug discovery: a comprehensive review. Pharmacology & therapeutics, 138(3), 333-408.
Hagberg, A., Swart, P., & S Chult, D. (2008). Exploring network structure, dynamics, and function using NetworkX (No. LA-UR-08-05495; LA-UR-08-5495). Los Alamos National Lab.(LANL), Los Alamos, NM (United States).
Jacobs, S., Khanna, A., & Madduri, K. Bader D. influenceR: Software Tools to Quantify Structural Importance of Nodes in a Network. 2015. https://cran.r-project.org/package=influenceR [accessed 7 April 2020]. R package version 0.1.0.
Jiang, C., & Liu, Z. (2019). Detecting multiple key players under the positive effect by using a distance-based connectivity approach. Physica A: Statistical Mechanics and its Applications, 534, 122322.
Jiang, C., Liu, Z., Wang, J., Yu, H., & Guo, X. (2017). An optimal approach for the critical node problem using semidefinite programming. Physica A: Statistical Mechanics and its Applications, 471, 315-324.

Lalou, M., Tahraoui, M. A., & Kheddouci, H. (2018). The critical node detection problem in networks: A survey. Computer Science Review, 28, 92-117.
Leskovec, J., & Sosič, R. (2016). Snap: A general-purpose network analysis and graph-mining library. ACM Transactions on Intelligent Systems and Technology (TIST), 8(1), 1-20.
Madduri, K., & Bader, D. A. (2009, May). Compact graph representations and parallel connectivity algorithms for massive dynamic network analysis. In 2009 IEEE International Symposium on Parallel & Distributed Processing (pp. 1-11). IEEE.
McGuire, R. M., Deckro, R. F., & Ahner, D. K. (2015). The weighted key player problem for social network analysis. Military Operations Research, 20(2), 35-53.
Paudel, N., Georgiadis, L., & Italiano, G. F. (2018). Computing critical nodes in directed graphs. Journal of Experimental Algorithmics, 23(2):2.2.
Peixoto, T. (2014). The graph-tool python library. figshare. DOI: 10.6084/m9.figshare.1164194
Schrödinger, LLC. The PyMOL Molecular Graphics System, Version 2.3.3.
Tomaino, V., Arulselvan, A., Veltri, P., & Pardalos, P. M. (2012). Studying connectivity properties in human protein-protein interaction network in cancer pathway. In Data Mining for Biomarker Discovery (pp. 187-197). Springer, Boston, MA.
Ventresca, M. (2012). Global search algorithms using a combinatorial unranking-based problem representation for the critical node detection problem. Computers & Operations Research, 39(11), 2763-2775.
Walteros, J. L., & Pardalos, P. M. (2012). Selected topics in critical element detection. In Applications of mathematics and informatics in military science (pp. 9-26). Springer, New York, NY.

> Irrespective of the success of this submission, we would be happy to keep in touch with this reviewer to get deeper into performance issues and new developments and applications of Pyntacle in ours and other research fields. We are grateful for this precise and in depth review.

Reviewer #2: In this article, the authors describe Pyntacle, a Python-based software tool for the analysis of networks. Specifically, they focus on identifying groups of nodes by performing a combinatorial evaluation, of various parameters/indices/centrality measures. The manuscript provides a good outline of the package and its capabilities. Their approach is explained well; I found Figure 1 to be a good toy example to illustrate the capabilities of the network. I have a few concerns for the authors to comment upon/implement to further improve their manuscript.

Specific comments:
1. I find the networks in Figure 5 to be very small and not representative of biological networks in complexity, size and density. It would be nice if the examples are changed to 10^3 and 10^4 nodes, discarding 100 nodes. Or, will the combinatorial complexity become a major problem for larger networks?

> As we have shown in the case study, with the network derived from a protein-protein interaction interface, the target of Pyntacle is represented also by small networks (<100 nodes/edges), therefore we wanted to present the different scenarios in which the potential user can find Pyntacle useful, e.g. residue contact or H-bond networks in a complex during a molecular dynamics simulation. Bigger networks (10^3 or 10^4 nodes) would not equally display the same level of informativeness in a Figure given their sizes; moreover, they would not represent a fair comparison ground with the other methods, since already it took more than one day of computation with networks in the 10^3 category, revealing the speedup gain when using Pyntacle. Finally, 10^4-node networks are indeed combinatorially complex to analyze, they will be properly dealt with in the next release of Pyntacle, with a stable GPU implementation and faster heuristic search algorithms and optimization approaches to choose from (as pointed out by reviewer 1 as well).

2. Perhaps, it would be nice if another example, of a protein-protein functional association network from the STRING database is considered. It would be a biologically motivating example, given the opening discussions about protein interaction networks.

> The C.elegans network, collected from APID and used for benchmarking and tests in the manuscript, is built from quality-checked protein-protein interaction data. We clarified this in the text.

3. Could any heuristics be used to cut down the combinatorial search, especially for larger networks. For instance, could nodes with a degree of 1 be ignored? Can the authors suggest some heuristics to simplify computations for larger networks, based on their observations of what nodes typically figure in

the "groups".

> The greedy optimization heuristics is shown to identify near-optimal solutions in the manuscript. It represents a fast and simple implementation, even if it is only a starting point. As pointed out by reviewer 1, there is a plethora of faster and more efficient heuristic search algorithms that we aim to include in Pyntacle, so that the user can select different algorithms of choice. (N.b., as a first reaction to this review, we have started and almost finished to implement another search algorithm, the Stochastic Gradient Descent, as it is verifiable in the Pyntacle's GitHub website, branch "feat#31"). We think this is the right path to follow. We do not recommend node (network leaves) pruning as it would greatly influence the outcome of different metrics (for example, the m-reach parameter would yield different results since the removed nodes cannot be reached).

4. `pyntacle test` works very well. Will it be possible to have `pyntacle demo` similarly, which can showcase some of the capabilities? Also, can some example networks be included?

> In order to gradually introduce the user to Pyntacle, we created a quick-start guide and 3 case studies, all detailed with data explanation, command lines and graphs both in the forms of HTML pages and python notebooks. We feel that a Pyntacle "demo" function would not be as welcoming to new users as the guides and case studies. Several examples can be downloaded directly from the quick-start guide/case studies in the website. Moreover, the adjacency matrices associated to the network analyzed in the two case studies can now be accessed from GigaDB.

5. An example case study with a larger network, such as E. coli from STRING would also add a lot of value to the paper.

> The STRING network for C.elegans is one order of magnitude bigger than the E.coli network from the same resource: the STRING C.elegans network counts 18181 nodes (6170 when considering only high-quality interactions with a combined score equal or higher than 900) while the STRING E.coli K12 W3110/MG1655 networks count respectively 4210/4125 nodes (which decrease to 2698/3259 nodes when applying the same high-quality filter). The C.elegans network we used (collected from the APID resource) counts 3303 nodes, same order of magnitude of the STRING networks, and therefore is a suitable representative of this kind of ppi networks. The number of edges greatly varies on the quality of each reported interaction.

Optional features:
1. Lastly, it would be nice if there is a way to pull networks from commonly used databases such as networkrepository.com or STRING. This would enable a lot of network scientists and biologists to easily perform various network analyses.

> This is a great option to be added in the immediate next release of Pyntacle, the user would be given the choice of selecting among the most used repositories (e.g. STRING, IMEx and APID).

Typographical errors:
Page 1, Para 1: Protein interaction networks, ... represents -> represent
Page 1, Para 1: These, -> unclear antecedent -- could we re-written
Page 1, Para 3: "having relatively few"" -> "having a few relatively"

> We corrected these errors. Thanks for this valuable review.

Close
