# Supplementary material for: Pyntacle: a parallel computing-enabled framework for large-scale network biology analysis
Source: Gigascience. 2020 Oct 21;9(10):giaa115. doi: 10.1093/gigascience/giaa115 (PMC7576925; doi:10.1093/gigascience/giaa115)
Supplement: giaa115_Response_to_Reviewer_Comments_Revision_1 [file giaa115_response_to_reviewer_comments_revision_1.pdf]

Author's Response To Reviewer Comments

Close

Dear editor and reviewers,
we have responded to all queries concerning our rev1 of this manuscript. In particular:
- [Editor] We have added the reference to our supplementary data stored in GigaDB;
- [Referee 1, A.1.4] We have added a reference to Lalou et al. (2018) and mentioned that to solve some larger problems it is possible to use more sophisticated exact algorithms such as those based on integer linear programming.
- [Referee 1, A.1.4] We have made it clearer in the manuscript that we have chosen to focus on the heuristic methods for scalability to large-scale networks, providing the brute-force method for exact solution only on the small (and yet biologically relevant) networks where it is practical.
- [Referee 1, A.2.1] We have replaced F with FUNC also in the headers of the algorithms.

Thank you once again for the accuracy of your editorial job and to help us improving the quality of our piece of work.

Close
