# Supplementary material for: Pyntacle: a parallel computing-enabled framework for large-scale network biology analysis
Source: Gigascience. 2020 Oct 21;9(10):giaa115. doi: 10.1093/gigascience/giaa115 (PMC7576925; doi:10.1093/gigascience/giaa115)
Supplement: giaa115_Response_to_Reviewer_Comments_Revision_2 [file giaa115_response_to_reviewer_comments_revision_2.pdf]

Author's Response To Reviewer Comments

Close

Dear editor,
I have registered Pyntacle in bio.tools and SciCrunch.org and I have indicated their ID in the proper section of the paper.
I have also added a title page with the list of our ORCID IDs. Please note that M. Truglio does not have any ORCID ID associated.

best regards and thanks
tommaso mazza

Close
