# Supplementary material for: Pyntacle: a parallel computing-enabled framework for large-scale network biology analysis
Source: Gigascience. 2020 Oct 21;9(10):giaa115. doi: 10.1093/gigascience/giaa115 (PMC7576925; doi:10.1093/gigascience/giaa115)
Supplement: giaa115_Response_to_Reviewer_Comments_Revision_3 [file giaa115_response_to_reviewer_comments_revision_3.pdf]

Author's Response To Reviewer Comments

Close

Sorry, I've erroneously deleted the license terms. Now I've added the item again and uploaded both the latex and bbl files.
regards, tommaso mazza

Close
