# Supplementary material for: Pyntacle: a parallel computing-enabled framework for large-scale network biology analysis
Source: Gigascience. 2020 Oct 21;9(10):giaa115. doi: 10.1093/gigascience/giaa115 (PMC7576925; doi:10.1093/gigascience/giaa115)
Supplement: giaa115_Reviewer_1_Report_Original_Submission — Alexander Stivala, PhD -- 4/12/2020 Reviewed [file giaa115_reviewer_1_report_original_submission.pdf]

Reviewer Report

Title: Pyntacle: a parallel computing-enabled framework for large-scale network biology analysis

Version: Original Submission **Date:** 4/12/2020

Reviewer name: Alexander Stivala, PhD

Reviewer Comments to Author:

"Pyntacle: a parallel computing-enabled framework for large-scale network biology analysis"
GigaScience manuscript (Technical Note) GIGA-D-20-00087
This manuscript describes a software library for graph computations usable as both a Python library and command line interface. In addition, it describes an interactive viewer output (Pyntacle-Ink) module for visualizing the results. Although Pyntacle is a general-purpose graph library, it has a particular focus (as does the manuscript) on group centrality metrics and the "key player problem" (KPP), which is the computationally intractable problem of finding sets of (rather than individual) nodes in a network which maximize (or minimize) a group centrality metric.
This distinguishes it from other general-purpose network packages, which usually do not include algorithms for the KPP, while those packages that do address it tend to be specific to that problem, and not general purpose libraries (see section C below).
Another distinguishing feature of Pyntacle is that it is targeted particularly at large-scale network biology analysis, and emphasizes parallel computing. Although the KPP is well-known in social network analysis (from which it originated), it is not so well-known in biology (although not unknown, see A.1.1 below) and so this manuscript makes a contribution in introducing it (and making available usable multi-platform software in a platform widely used in bioinformatics, i.e. Python) to the field.
A. Notes on the manuscript

The manuscript is generally clear, giving a (quite lengthy) introduction and exposition of the KPP, description of the software and algorithms implemented, benchmark results, and two case studies.
The software (both source code and installation packages), documentation, and data sets required for reproducibility are all referenced and freely available from the project website (although see A.1.3 below for an exception).
A.1. Major issues
These are points I think must be addressed before the manuscript can be accepted.
A.1.1. Insufficient references to existing research literature.
The KPP originated in social network research with the "key player problem" so named by Borgatti (2006). However in other fields, and specifically computer science and applied mathematics (operations research for example), the KPP and variants are known as "critical element detection" or the "critical node problem" (CNP) (Walteros & Pardalos, 2012), or the "critical node detection problem" (CNDP), of which the CNP is a variant (Lalou et al. (2018)).
This is an active research field, as evidenced by the review paper Lalou et al. (2018), and although a review of this field is clearly outside the scope of this manuscript (technical note), at least a brief

mention of the relationship of the KPP to the CNDP, and a citation of (for example) the Lalou et al. (2018) review paper would be of value to readers and not leave them with the misleading impression that the KPP has only been studied in a couple of papers and only the brute-force and greedy heuristic algorithms have been studied to solve it.
As well as this general issue, I think some references to the prior use of the KPP (or related CNDP variants) in biology would be welcome, for example in protein-protein interaction (PPI) networks (Boginsnki & Commander, 2009; Tomaino et al., 2012), and genetic regulatory networks (Celestini et al., 2019). For a more general perspective on network methods in drug discovery, the extensive review of Csermely et al. (2013) could be informative.
In the concluding remarks (p. 8) where extension to weighted and directed networks is mentioned, it might be noted that some work exists on the CNDP in directed graphs (Paudel et al., 2018) and KPP on weighted graphs (McGuire et al., 2015; Yang, 2015; Jiang & Liu, 2019). Indeed the R keyplayer package is claimed to work with both directed and weighted networks (An & Liu, 2016).
Although reproducing results from the many algorithms for solving the KPP or CNDP variants (either exactly i.e. to optimality, or to potentially suboptimal solutions with heuristics) is clearly impractical, rough comparisons to other methods could be facilitated by running the benchmarks on the Critical Node Detection Problem Benchmark Instances (Ventresca 2012), available from http://individual.utoronto.ca/mventresca/cnd.html, which are used in many CNDP papers.
A.1.2. Insufficient references to existing software.
Although a review of existing network libraries is beyond the scope of the manuscript, it could be useful for readers to at least mention some other general purpose graph libraries usable from Python such as graph-tool (Peixoto, 2014), igraph (Csardi & Nepusz, 2006), NetworkX (Hagberg et al., 2008), and SNAP (Leskovec & SosiÄ�, 2016). (See Section C below for more).
Most particularly, it is noted that Pyntacle uses igraph modules, yet the igraph paper (Csardi & Nepusz, 2006) is not cited.
In addition, although the Analytic Technologies KeyPlayer program and the R keyplayer package (An & Liu, 2016) are mentioned, the influenceR R package (Jacobs et al., 2015) is not mentioned. Although this package only solves KPP-Pos with the DF metric, as I show in Section B below (on a single example) it does so much faster than Pyntacle.
A.1.3. Reproducibility (data availability) of case studies.
For Case Study 1, I was easily able to reproduce the 3D protein cartoon in Fig. 6 by downloading the 5XTD structure from the RCSB PDB into PyMOL (SchrÃ¶dinger, LLC), selecting the appropriate two chains, and locating the key player residues as shown. However to obtain the network of "The 138 residues forming the interaction interface" I would (according to the Methods) have to conduct several operations in FoldX and then extract the network according to a distance threshold described in the Methods. Although it seems there is sufficient detail to reproduce this, it requires a registration to use FoldX and some further work construct the network.
I think it would assist reproducibility (an important consideration in the GigaScience journal) to supply the network as constructed by the authors as a Supplementary Information file (as for example the results of the Pyntacle analysis are already supplied in an Excel file).
Similarly, the network constructed for Case Study 2 could also be made available as Supplementary Information.

A.1.4. Use of "brute-force" algorithm and "large-scale".
The manuscript is described (including in the title) as being for "large-scale" network analysis, and parallelism is emphasized. However (related to point A.1.1 above) it seems to me that the emphasis is perhaps misplaced. To be specific, two algorithms are implemented and benchmarked: the "brute-force" algorithm, and the greedy heuristic algorithm of Borgatti (2006). Parallelism then mainly seems to be discussed in relation to the brute-force algorithm.
However, as the KPP is known to be computationally intractable and indeed NP-complete (Arulselvan et al., 2009), a brute-force algorithm is never going to be practical, and certainly not for large-scale instances. For this reason there are two practical approaches: (a) exact solution by more sophisticated methods (for example integer linear programming (ILP), semidefinite programming (Jiang et al., 2017), branch-and-cut methods, etc.), and (b) heuristic algorithms to find a potentially suboptimal solution, but much faster. Note that the latter are also vital as input into the former (as bounds on the solution). For a review of these approaches see Lalou et al. (2018).
Hence my concern is that implementing and applying parallel computation to the brute-force approach is misplaced, as it can never be competitive with sophisticated exact solution algorithms (particularly where these could also be parallelized) on large-scale problems.
Regarding the heuristic (greedy) algorithm, it also seems that better results could be obtained with a more sophisticated heuristic, as already foreshadowed by Borgatti (2006). For example I demonstrate in Section B below, that the influenceR program is two orders of magnitude faster (albeit on a single example) than Pyntacle for KPP-Pos, as it uses a different heuristic algorithm. In addition there are many publications showing superior results with different heuristic algorithms for CNDP variants (see Lalou et al., 2018 for a review).
A.1.5. Details of parallelism and benchmarks.
From my understanding of the manuscript (and some experiments I tried with it detailed in Section B below), there are two types of parallelism used in Pyntacle. First, the "fine grained" parallelism it obtains simply from using the Numba library, and second, explicit "coarse grained" parallelism. The first type uses multithreading in lower level numeric libraries, similar to Microsoft R/Open using multithreaded linear algebra routines. The second type is used only for the brute-force algorithm however (and on Linux seems to work by forking new processes rather than using threads).
It is not entirely clear, but it seems to me that Figure 4 shows results for using the greedy heuristic with a single thread (no parallelism). Thus it compares the same heuristic algorithm implemented in Pyntacle and R/keyplayer, showing that Pyntacle is faster (due to implementation details, being in Python not R, etc.).
This is a fair comparison if both implementations are really using only a single thread. However I found that Pyntacle used multithreading (up to the number of cores on the node it is run on) automatically, unless the NUMBA_NUM_THREADS environment variable is set (I am not familiar with the Numba library and had to find this in its online documentation). I could not find any documentation in the Pyntacle online manuals about parallelism or adjusting the number of threads, except for the -T/--threads option which applies to the brute-force algorithm only (not the greedy heuristic).
The keyplayer R package, however, uses a single thread unless explicitly requested to use parallelism by a function argument.
Hence I think this should be clarified in the manuscript, and some documentation of how parallelism

works and how to adjust the number of threads added to the Pyntacle documentation.
Fig. 5, however, shows the speedup obtained by Pyntacle using the brute-force algorithm with explicit (coarse-grained) parallelism as described, of computing the metric on the independent possible node sets in parallel. This is a valid comparison, however as per A.1.4 above, I question the practicality of the brute-force method at all, given the availability of many more efficient (albeit more complicated, and perhaps requiring a sophisticated ILP solver for example) algorithms for exact solution.
A.1.6. Graphics processing unit (GPU).
The manuscript mentions graphics processing units (GPU) for parallelism, but I found no further details on this. When I run Pyntacle on a node with no GPU, I get a warning message that "NVIDIA: no NVIDIA devices found" which would seem to indicate that NVIDIA (CUDA) GPU support is available, and when I run the pyntacle keyplayer module on a cluster node which does have a GPU, I do not get the warning message, but the GPU does not seem to be used (checking with the nvidia-smi program).
Note that I installed the 'miniconda' package on a cluster head node which does not have a GPU (details in Section B below).

A.2. Minor issues
A.2.1. Some notational confusion with 'F'.
The variable F is first mentioned in Case Study 1 (p. 7 "... the best negative key-players in the network (according to both F and DF metrics), ...") but not defined, although it presumably refers to the fragmentation centrality of Borgatti (2006, equation (4)). However it is then used as a variable representing any centrality function (key-player metric) in Algorithms 1 and 2 (p. 9), which could be confusing.
A.2.2. Some potential confusion for readers between KeyPlayer (Analytic Technologies) and R "keyplayer" package.
At the bottom of p. 5 (column 1): "... keyplayer is not rigorously testable here since it is a Windows-only GUI-based application." This could be confusing as the R keyplayer package (An & Liu, 2016) is previously styled as "keyplayer" (lowercase) while the Analytic Technologies KeyPlayer program (Borgatti, 2019) is styled as "KeyPlayer" ("camel case"), and it is the Analytic Technologies KeyPlayer program that is Windows-only.

B. Notes on the Pyntacle software

The Pyntacle website referenced in the manuscript (http://pyntacle.css-mendel.it) is easy to navigate and read, and contains extensive documentation on installation and usage, as well as, importantly, examples and case studies which can be followed and reproduced easily.
I installed the Pyntacle software on a Linux server (the master [head] node of the cluster described below, which has 2 x Intel E5-2630 v3, 16 (2 x 8) cores CPU,64GB DDR4 @ 2133MHz RAM) using the 'miniconda' system as recommended on the Pyntacle home page. This worked without problem, and I ran the tests which passed. I was also easily able to reproduce Case Study 3 (C. elegans connectome) linked from the software home page.
I then ran some of my own tests (using the command line interface, running as batch jobs on cluster compute nodes) to compare results between Pyntacle, the keyplayer (v1.0.3) R package (An & Liu,

2016) and (for KPP-Pos only) the influenceR R package (v0.l.0) (Jacobs et al., 2015).
I came across one bug in the command line interface: the --type m-reach option does not seem to work. But I found this already reported in the GitHub issue tracker as issue #23.
Note I ran the tests once only, so the times (which are elapsed times of the key player finding module only, as reported by Pyntacle itself, or system.time() for R packages) are merely indicative. For parallel tests I reserved all 20 cores on a compute node. For serial tests I reserved a single core on a compute node. I set environment variable NUMBA_NUM_THREADS for Pyntacle and OMP_NUM_THREADS for R packages using OpenMP, and the parallel flag and cluster option for the keyplayer R package, to the appropriate number of cores (20 or 1) to ensure the correct number of cores is (potentially) used.
All tests I conducted were on a Linux (CentOS 7.5 x86_64) cluster with 42 compute nodes and Intel 40Gbps QDR Infiniband. The compute nodes used have 2 x Intel Xeon E5-2650 v3 @ 2.30GHz, 20 (2 x 10) cores CPU, 64GB DDR4 @ 2133MHz RAM. The node with a GPU is 2 x Intel Xeon E5-2650 v3 @ 2.30GHz, 20 (2 x 10) cores CPU, 128GB DDR4 @ 2133MHz RAM, GPU:1 x NVIDIA GeForce GTX 1080 Founders Edition 8GB GDDR5X 2560 CUDA cores. The cluster scheduling system is slurm and the R version I used is Microsoft R Open v3.2.5.
Test 1: Key players DF (KPP-Neg) of size k = 2 in the C. elegans connectome (279 nodes), computed with greedy heuristic.
The network data set used is the CAEEL_Connectome.sif file as used in "Case Study 3" from the Pyntacle home page. I used the "pyntacle convert" command to convert it from SIF format to edgelist format for use in the R keyplayers package. Note that the influenceR package only does KPP-Pos so it is not included in this test.

Method	DF value	Solutions	Elapsed time (s)
Pyntacle serial	0.58991	AVAL, AVAR	18.73
Pyntacle parallel	0.58991	AVAL, AVAR	18.17
R/keyplayer serial	0.5899131	AVAL, AVAR	1940.055
R/keyplayer parallel	0.5899131	AVAL, AVAR	9576.714

So this shows Pyntacle computing the KPP-Neg (DF metric) correctly on this test, two orders of magnitude faster than the R keyplayer software. There is no real difference in the time for Pyntacle on 1 core versus 20 cores, perhaps unimportant when it takes less than 20 seconds anyway. I'm not sure why the keyplayer R package is so much slower with 20 cores than 1 core, but I suspect it is because the parallel mode in that package is for trying to avoid local minima by running the algorithm with different randomization seeds in parallel (An & Liu 2016, p. 264) rather than speeding the computation of a single run.
In running these tests I found a bug in Pyntacle-Ink (the HTML graphical output): for KPP-Neg metric dF it reports both "Initial Value" and "Value" but they appear to be swapped (they are the right way around in the stdout and the Report TSV file however).
Test 2: Key players DR (KPP-Pos) of size k = 2 in the C. elegans PPI network (3303 nodes), computed with greedy heuristic. (This should be the same test as the benchmark shown in Fig. 4 of the manuscript for (D)).
The network data used here is the APID_CAEEL_Level2_maincomponent.adjm adjacency matrix file as used in "Case Study 2" linked from the Pyntacle software home page, and referred to as "APID" in the manuscript.

Method	DR value	Solutions	Elapsed time (s)
Pyntacle serial	0.37486	Q22174, Q95QA6	416.70
Pyntacle parallel	0.37486	Q22174, Q95QA6	353.84
influenceR serial	[not shown]	O45521, Q95QA6	1.208
influenceR parallel	[not shown]	Q22174, Q95QA6	2.600
R/keyplayer serial	[did not complete within 20 hour elapsed time limit]		
R/keyplayer parallel	[did not complete within 20 hour elapsed time limit]		

So it seems to be working correctly, and on my test took about 400 seconds, compared to about 200 seconds in the manuscript Fig. 4, so this seems reasonable. And again, much faster than the keyplayer R package (which could not find a solution within 20 hours).

Again, however, using Pyntacle key player module using the greedy heuristic with 20 cores has only a relatively small advantage over a single core, even though the elapsed time is now several minutes. The Linux (tcsh) time builtin shows 99.8% CPU for the serial run and only 109.0% CPU for the parallel run, showing that little parallelism was used.

However, note that the influenceR R package, which uses a different heuristic algorithm, "stochastic gradient descent" (Jacobs et al., 2015, "keyplayer") is able to find the same solution as Pyntacle, but two orders of magnitude faster (note it also finds a different solution in the serial run - since it is a stochastic algorithm we could get different values each run - but unfortunately does not seem to report the objective value so we cannot see if it is a better or worse solution).

Note also that influenceR (despite the short elapsed time) also seems to be making good use of multicore (20 cores): the (Linux) time command shows similar user and elapsed time for the serial run (as expected), but the parallel run shows 26.137 user CPU seconds and only 1.208 elapsed seconds (and only 0.016 system).

Note I also tried to find the key player set size k = 2 for the DF metric (KPP-Neg) with the greedy heuristic in Pyntacle in this data set, but it was unable to complete with the time limit of 20 hours (serial or parallel).

C. Network software libraries/packages referenced

This list is not necessarily exhaustive, but are some packages that I have used or are aware of, particularly those that are usable from Python. They are all general-purpose graph libraries, with the exception of influenceR and keyplayer, which are specifically for the key players problem. Some have many more features than others though; for example SNAP (Leskovec) handles very large networks efficiently, but has very few algorithms implemented compared to igraph or NetworkX).

Name	Website	Citation	Interface	Implementation	Parallel	KeyPlayers
graph-tool	https://graph-tool.skewed.de/	Peixoto (2014)	Python	C++, Boost	OpenMP	No
igraph	https://igraph.org/	Csardi & Nepusz (2006)	Python,R,C/C++,Mathematica	C	No	No
influenceR	https://cran.r-project.org/web/packages/influenceR/index.html	Jacobs et al. (2015)	R	R, igraph, SNAP(Bader)	OpenMP	KPP-Pos(DR)
keyplayer	https://cran.r-project.org/web/packages/keyplayer/index.html	An & Liu (2016)				

R R, igraph, sna R/parallel Yes

NetworkX http://networkx.github.io/ Hagberg et al. (2008)

Python Python, SciPy No No

sna (statnet) https://cran.r-project.org/web/packages/sna/index.html Butts (2008)

R C No No

SNAP (Bader) http://snap-graph.sourceforge.net/ Bader & Madduri (2008);

Madduri & Bader (2009) C C OpenMP No

SNAP (Leskovec) http://snap.stanford.edu/ Leskovec & SosiÄ(2016)

Python, C++, NodeXL C++ Maybe? No

D. References (in addition to those in manuscript)

Arulselvan, A., Commander, C. W., Elefteriadou, L., & Pardalos, P. M. (2009). Detecting critical nodes in sparse graphs. Computers & Operations Research, 36(7), 2193-2200.

Bader, D. A., & Madduri, K. (2008, April). Snap, small-world network analysis and partitioning: An open-source parallel graph framework for the exploration of large-scale networks. In 2008 IEEE international symposium on parallel and distributed processing (pp. 1-12). IEEE.

Boginski, V., & Commander, C. W. (2009). Identifying critical nodes in protein-protein interaction networks. In Clustering challenges in biological networks (pp. 153-167).

Butts, C. T. (2008). Social network analysis with sna. Journal of statistical software, 24(6), 1-51.

Celestini, A., Cianfriglia, M., Mastrostefano, E., Palma, A., Castiglione, F., & Tieri, P. (2019). Critical nodes reveal peculiar features of human essential genes and protein interactome. bioRxiv, 831750.

Csardi, G., & Nepusz, T. (2006). The igraph software package for complex network research. InterJournal, complex systems, 1695(5), 1-9.

Csermely, P., KorcsmÃ¡ros, T., Kiss, H. J., London, G., & Nussinov, R. (2013). Structure and dynamics of molecular networks: a novel paradigm of drug discovery: a comprehensive review. Pharmacology & therapeutics, 138(3), 333-408.

Hagberg, A., Swart, P., & S Chult, D. (2008). Exploring network structure, dynamics, and function using NetworkX (No. LA-UR-08-05495; LA-UR-08-5495). Los Alamos National Lab.(LANL), Los Alamos, NM (United States).

Jacobs, S., Khanna, A., & Madduri, K. Bader D. influenceR: Software Tools to Quantify Structural Importance of Nodes in a Network. 2015. https://cran.r-project.org/package=influenceR [accessed 7 April 2020]. R package version 0.1.0.

Jiang, C., & Liu, Z. (2019). Detecting multiple key players under the positive effect by using a distance-based connectivity approach. Physica A: Statistical Mechanics and its Applications, 534, 122322.

Jiang, C., Liu, Z., Wang, J., Yu, H., & Guo, X. (2017). An optimal approach for the critical node problem using semidefinite programming. Physica A: Statistical Mechanics and its Applications, 471, 315-324.

Lalou, M., Tahraoui, M. A., & Kheddouci, H. (2018). The critical node detection problem in networks: A survey. Computer Science Review, 28, 92-117.

Leskovec, J., & SosiÄ, R. (2016). Snap: A general-purpose network analysis and graph-mining library. ACM Transactions on Intelligent Systems and Technology (TIST), 8(1), 1-20.

Madduri, K., & Bader, D. A. (2009, May). Compact graph representations and parallel connectivity algorithms for massive dynamic network analysis. In 2009 IEEE International Symposium on Parallel & Distributed Processing (pp. 1-11). IEEE.
McGuire, R. M., Deckro, R. F., & Ahner, D. K. (2015). The weighted key player problem for social network analysis. Military Operations Research, 20(2), 35-53.
Paudel, N., Georgiadis, L., & Italiano, G. F. (2018). Computing critical nodes in directed graphs. Journal of Experimental Algorithmics, 23(2):2.2.
Peixoto, T. (2014). The graph-tool python library. figshare. DOI: 10.6084/m9.figshare.1164194
SchrÃ¶dinger, LLC. The PyMOL Molecular Graphics System, Version 2.3.3.
Tomaino, V., Arulselvan, A., Veltri, P., & Pardalos, P. M. (2012). Studying connectivity properties in human protein-protein interaction network in cancer pathway. In Data Mining for Biomarker Discovery (pp. 187-197). Springer, Boston, MA.
Ventresca, M. (2012). Global search algorithms using a combinatorial unranking-based problem representation for the critical node detection problem. Computers & Operations Research, 39(11), 2763-2775.
Walteros, J. L., & Pardalos, P. M. (2012). Selected topics in critical element detection. In Applications of mathematics and informatics in military science (pp. 9-26). Springer, New York, NY.

Level of Interest

Please indicate how interesting you found the manuscript: Choose an item.

Quality of Written English

Please indicate the quality of language in the manuscript: Choose an item.

Declaration of Competing Interests

Please complete a declaration of competing interests, considering the following questions:

- Have you in the past five years received reimbursements, fees, funding, or salary from an organisation that may in any way gain or lose financially from the publication of this manuscript, either now or in the future?
- Do you hold any stocks or shares in an organisation that may in any way gain or lose financially from the publication of this manuscript, either now or in the future?
- Do you hold or are you currently applying for any patents relating to the content of the manuscript?
- Have you received reimbursements, fees, funding, or salary from an organization that holds or has applied for patents relating to the content of the manuscript?
- Do you have any other financial competing interests?
- Do you have any non-financial competing interests in relation to this paper?

If you can answer no to all of the above, write 'I declare that I have no competing interests' below. If your reply is yes to any, please give details below.

I declare that I have no competing interests.

I agree to the open peer review policy of the journal. I understand that my name will be included on my report to the authors and, if the manuscript is accepted for publication, my named report including any attachments I upload will be posted on the website along with the authors' responses. I agree for my report to be made available under an Open Access Creative Commons CC-BY license (http://creativecommons.org/licenses/by/4.0/). I understand that any comments which I do not wish to be included in my named report can be included as confidential comments to the editors, which will not be published.

Choose an item.

To further support our reviewers, we have joined with Publons, where you can gain additional credit to further highlight your hard work (see: https://publons.com/journal/530/gigascience). On publication of this paper, your review will be automatically added to Publons, you can then choose whether or not to claim your Publons credit. I understand this statement.

Yes Choose an item.
