# Supplementary material for: Pyntacle: a parallel computing-enabled framework for large-scale network biology analysis
Source: Gigascience. 2020 Oct 21;9(10):giaa115. doi: 10.1093/gigascience/giaa115 (PMC7576925; doi:10.1093/gigascience/giaa115)
Supplement: giaa115_Reviewer_1_Report_Revision_1 — Alexander Stivala, PhD -- 7/14/2020 Reviewed [file giaa115_reviewer_1_report_revision_1.pdf]

Reviewer Report

Title: Pyntacle: a parallel computing-enabled framework for large-scale network biology analysis

Version: Revision 1 **Date:** 7/14/2020

Reviewer name: Alexander Stivala, PhD

Reviewer Comments to Author:

I thank the authors for taking the time to consider the points in my review. As detailed below in points A.1.1 and A.1.4 [and a minor point in A.2.1], I believe the manuscript requires only minor changes now.
A.1.1. Insufficient references to existing research literature.
There were really two separate issues under this point: first, the actual mention of this work work and relevant references, and second, some benchmarking exercise against (some) other published algorithms/software.
The first issue has been sufficiently addressed by adding several relevant references.
The second issue, however, involves much more work, and the authors choose to defer it until a later software release, in which more heuristic methods will be implemented, with which comparisons can be made.
I accept that this can be addressed by noting this as future (ongoing) work, as long as my point about putting more context or description of the newly cited works [A.1.4 below] is addressed in another (minor) revision of this manuscript.
A.1.2. Insufficient references to existing software.
References to appropriate related software have been added to address this comment satisfactorily.
A.1.3. Reproducibility (data availability) of case studies.
This has been addressed by supplying the relevant adjacency matrices to GigaDB (although due to apparent technical problems I was unable to download them via FTP from their temporary location, however I trust that they will be available).
Moreover the note that the interface residues can in fact be obtained directly from the PDB structure data simply by a distance threshold (easily done in the Python environment with Bio.PDB or PyMol for example) without actually having to use FoldX means they should be easily reproducible anyway.
A.1.4. Use of "brute-force" algorithm and "large-scale".
It seems I misunderstood, and parallelism is indeed applied to both the brute-force (coarse- and fine-grained) and heuristic algorithms (only fine-grained), as the authors explain in their reply.
The brute-force method is indeed appropriate, at least as a baseline for comparison, for small networks (maybe a few hundred or smaller) for which it is possible, so it should be included.
However is it not a given that the only way to handle larger networks (for which brute-force is infeasible) is a heuristic (such as the greedy algorithm in the manuscript, or the newly implemented stochastic gradient descent). It may indeed be feasible, up to some size limit, to solve some larger problems to optimality using more sophisticated exact algorithms such as those based on integer linear programming (ILP). For example the Lalou et al. (2018) review paper shows an example where an ILP method can find the optimal result for the CNP on a network with 5000 nodes from the Ventresca

(2012) benchmark set.
However as the networks get larger still, heuristics will become necessary.
I think the authors could still make it clearer in the manuscript that they have chosen to focus on the heuristic method(s) for scalability to large-scale networks, providing the brute-force method for exact solution only on the small (and yet biologically relevant) networks where it is practical, and cite some of the exact methods (and/or the review paper Lalou et al. 2018) in this context, noting that there exist methods to solve to optimality more efficiently (and for larger instances than the small ones possible with brute-force) than the brute-force algorithm, rather than just the "in passing" citation added in the Background section to address point A.1.1 (".. specific search strategies [13,14] were implemented.").
A.1.5. Details of parallelism and benchmarks.
Due to the detailed and technical nature of this discussion which is perhaps not suitable to include in detail in a paper, I am satisfied that this has been addressed in the reply to my comments and the improvements to the software (online) documentation.
A.1.6. Graphics processing unit (GPU).
This has been sufficiently addressed by adding a note in the manuscript that these experimental features are only accessible via the API for now.
A.2.1. Some notational confusion with 'F'.
This point has been addressed by replacing 'F' with 'FUNC', however there was an omission in not doing so for the procedure signatures (line 1 in Algorithm 1 and also Algorithm 2) where it still has "F: lambda" rather than "FUNC: lambda), which is easily fixed.
Tbe other minor points have all been addressed satisfactorily.

Level of Interest

Please indicate how interesting you found the manuscript: Choose an item.

Quality of Written English

Please indicate the quality of language in the manuscript: Choose an item.

Declaration of Competing Interests

Please complete a declaration of competing interests, considering the following questions:

- Have you in the past five years received reimbursements, fees, funding, or salary from an organisation that may in any way gain or lose financially from the publication of this manuscript, either now or in the future?
- Do you hold any stocks or shares in an organisation that may in any way gain or lose financially from the publication of this manuscript, either now or in the future?
- Do you hold or are you currently applying for any patents relating to the content of the manuscript?

- Have you received reimbursements, fees, funding, or salary from an organization that holds or has applied for patents relating to the content of the manuscript?
- Do you have any other financial competing interests?
- Do you have any non-financial competing interests in relation to this paper?

If you can answer no to all of the above, write 'I declare that I have no competing interests' below. If your reply is yes to any, please give details below.

I declare that I have no competing interests.

I agree to the open peer review policy of the journal. I understand that my name will be included on my report to the authors and, if the manuscript is accepted for publication, my named report including any attachments I upload will be posted on the website along with the authors' responses. I agree for my report to be made available under an Open Access Creative Commons CC-BY license (http://creativecommons.org/licenses/by/4.0/). I understand that any comments which I do not wish to be included in my named report can be included as confidential comments to the editors, which will not be published.

Choose an item.

To further support our reviewers, we have joined with Publons, where you can gain additional credit to further highlight your hard work (see: https://publons.com/journal/530/gigascience). On publication of this paper, your review will be automatically added to Publons, you can then choose whether or not to claim your Publons credit. I understand this statement.

Yes Choose an item.
