# Supplementary material for: Pyntacle: a parallel computing-enabled framework for large-scale network biology analysis
Source: Gigascience. 2020 Oct 21;9(10):giaa115. doi: 10.1093/gigascience/giaa115 (PMC7576925; doi:10.1093/gigascience/giaa115)
Supplement: giaa115_Reviewer_2_Report_Original_Submission — Karthik Raman -- 4/28/2020 Reviewed [file giaa115_reviewer_2_report_original_submission.pdf]

Reviewer Report

Title: Pyntacle: a parallel computing-enabled framework for large-scale network biology analysis

Version: Original Submission **Date:** 4/28/2020

Reviewer name: Karthik Raman

Reviewer Comments to Author:

In this article, the authors describe Pyntacle, a Python-based software tool for the analysis of networks. Specifically, they focus on identifying groups of nodes by performing a combinatorial evaluation, of various parameters/indices/centrality measures. The manuscript provides a good outline of the package and its capabilities. Their approach is explained well; I found Figure 1 to be a good toy example to illustrate the capabilities of the network. I have a few concerns for the authors to comment upon/implement to further improve their manuscript.

Specific comments:

1. I find the networks in Figure 5 to be very small and not representative of biological networks in complexity, size and density. It would be nice if the examples are changed to 10^3 and 10^4 nodes, discarding 100 nodes. Or, will the combinatorial complexity become a major problem for larger networks?
2. Perhaps, it would be nice if another example, of a protein-protein functional association network from the STRING database is considered. It would be a biologically motivating example, given the opening discussions about protein interaction networks.
3. Could any heuristics be used to cut down the combinatorial search, especially for larger networks. For instance, could nodes with a degree of 1 be ignored? Can the authors suggest some heuristics to simplify computations for larger networks, based on their observations of what nodes typically figure in the "groups".
4. `pyntacle test` works very well. Will it be possible to have `pyntacle demo` similarly, which can showcase some of the capabilities? Also, can some example networks be included?
5. An example case study with a larger network, such as E. coli from STRING would also add a lot of value to the paper.

Optional features:

1. Lastly, it would be nice if there is a way to pull networks from commonly used databases such as networkrepository.com or STRING. This would enable a lot of network scientists and biologists to easily perform various network analyses.

Typographical errors:

Page 1, Para 1: Protein interaction networks, ... represents -> represent

Page 1, Para 1: These, -> unclear antecedent -- could we re-written

Page 1, Para 3: "having relatively few"" -> "having a few relatively"

Level of Interest

Please indicate how interesting you found the manuscript: Choose an item.

Quality of Written English

Please indicate the quality of language in the manuscript: Choose an item.

Declaration of Competing Interests

Please complete a declaration of competing interests, considering the following questions:

- Have you in the past five years received reimbursements, fees, funding, or salary from an organisation that may in any way gain or lose financially from the publication of this manuscript, either now or in the future?
- Do you hold any stocks or shares in an organisation that may in any way gain or lose financially from the publication of this manuscript, either now or in the future?
- Do you hold or are you currently applying for any patents relating to the content of the manuscript?
- Have you received reimbursements, fees, funding, or salary from an organization that holds or has applied for patents relating to the content of the manuscript?
- Do you have any other financial competing interests?
- Do you have any non-financial competing interests in relation to this paper?

If you can answer no to all of the above, write 'I declare that I have no competing interests' below. If your reply is yes to any, please give details below.

I declare that I have no competing interests

I agree to the open peer review policy of the journal. I understand that my name will be included on my report to the authors and, if the manuscript is accepted for publication, my named report including any attachments I upload will be posted on the website along with the authors' responses. I agree for my report to be made available under an Open Access Creative Commons CC-BY license (http://creativecommons.org/licenses/by/4.0/). I understand that any comments which I do not wish to be included in my named report can be included as confidential comments to the editors, which will not be published.

Choose an item.

To further support our reviewers, we have joined with Publons, where you can gain additional credit to further highlight your hard work (see: https://publons.com/journal/530/gigascience). On publication of this paper, your review will be automatically added to Publons, you can then choose whether or not to claim your Publons credit. I understand this statement.

Yes Choose an item.
